# Comprehensive review of the evidence regarding the effectiveness of community–based primary health care in improving maternal, neonatal and child health: 1. rationale, methods and database description

**DOI:** 10.7189/jogh.07.010901

**Published:** 2017-06

**Authors:** Henry B Perry, Bahie M Rassekh, Sundeep Gupta, Jess Wilhelm, Paul A Freeman

**Affiliations:** 1 Department of International Health, Johns Hopkins Bloomberg School of Public Health, Baltimore, Maryland, USA; 2 The World Bank, Washington DC, USA; 3 Medical Epidemiologist, Lusaka, Zambia; 4 Independent consultant, Seattle, Washington, USA; 5 Department of Global Health, University of Washington, Seattle, Washington, USA

## Abstract

**Background:**

Community–based primary health care (CBPHC) is an approach used by health programs to extend preventive and curative health services beyond health facilities into communities and even down to households. Evidence of the effectiveness of CBPHC in improving maternal, neonatal and child health (MNCH) has been summarized by others, but our review gives particular attention to not only the effectiveness of specific interventions but also their delivery strategies at the community level along with their equity effects. This is the first article in a series that summarizes and analyzes the assessments of programs, projects, and research studies (referred to collectively as projects) that used CBPHC to improve MNCH in low– and middle–income countries. The review addresses the following questions: (1) What kinds of projects were implemented? (2) What were the outcomes of these projects? (3) What kinds of implementation strategies were used? (4) What are the implications of these findings?

**Methods:**

12 166 reports were identified through a search of articles in the National Library of Medicine database (PubMed). In addition, reports in the gray literature (available online but not published in a peer–reviewed journal) were also reviewed. Reports that describe the implementation of one or more community–based interventions or an integrated project in which an assessment of the effectiveness of the project was carried out qualified for inclusion in the review. Outcome measures that qualified for inclusion in the review were population–based indicators that defined some aspect of health status: changes in population coverage of evidence–based interventions or changes in serious morbidity, in nutritional status, or in mortality.

**Results:**

700 assessments qualified for inclusion in the review. Two independent reviewers completed a data extraction form for each assessment. A third reviewer compared the two data extraction forms and resolved any differences. The maternal interventions assessed concerned education about warning signs of pregnancy and safe delivery; promotion and/or provision of antenatal care; promotion and/or provision of safe delivery by a trained birth attendant, screening and treatment for HIV infection and other maternal infections; family planning, and; HIV prevention and treatment. The neonatal and child health interventions that were assessed concerned promotion or provision of good nutrition and immunizations; promotion of healthy household behaviors and appropriate utilization of health services, diagnosis and treatment of acute neonatal and child illness; and provision and/or promotion of safe water, sanitation and hygiene. Two–thirds of assessments (63.0%) were for projects implementing three or fewer interventions in relatively small populations for relatively brief periods; half of the assessments involved fewer than 5000 women or children, and 62.9% of the assessments were for projects lasting less than 3 years. One–quarter (26.6%) of the projects were from three countries in South Asia: India, Bangladesh and Nepal. The number of reports has grown markedly during the past decade. A small number of funders supported most of the assessments, led by the United States Agency for International Development. The reviewers judged the methodology for 90% of the assessments to be adequate.

**Conclusions:**

The evidence regarding the effectiveness of community–based interventions to improve the health of mothers, neonates, and children younger than 5 years of age is growing rapidly. The database created for this review serves as the basis for a series of articles that follow this one on the effectiveness of CBPHC in improving MNCH published in the Journal of Global Health. These findings, together with recommendations provided by an Expert Panel which has guided this review, that are included as the last paper in this series, will help to provide the rationale for building stronger community–based platforms for delivering evidence–based interventions in high–mortality, resource–constrained settings.

The evidence that community–based interventions can improve maternal, neonatal and child health (MNCH) has been steadily growing over the past several decades [1–3]. Nonetheless, community–based primary health care (CBPHC) as an approach for engaging communities and delivering health interventions to communities and even down to each household remains an underdeveloped component of health systems in most resource–constrained settings. Except for immunizations and vitamin A supplementation, population coverage levels of evidence–based MNCH interventions in the countries with 97% of the world’s maternal, neonatal and child deaths remains around 50% or less [4]. The evidence regarding the effectiveness of individual interventions provided at the community level continues to grow. We now stand in a moment of time in which the era of the United Nations’ Millennium Development Goals has ended (2000–2015) and the era of the Sustainable Development Goals has begun (2015–2030). Thus, now is an opportune time to take stock of the evidence regarding the effectiveness of community–based approaches in improving MNCH and the approaches that have been used to achieve effectiveness.

Even though major gains have been made around the world in reducing maternal, neonatal, and child mortality (MNCH), 8.8 million maternal deaths, stillbirths, neonatal deaths, and deaths of children 1–59 months of age occur each year, mostly from readily preventable or treatable conditions [5]. Only four of the 75 countries with 97% of the world’s maternal, perinatal, neonatal and child deaths were able to achieve both Millennium Development Goal (MDG) 4 (which called for a two–thirds reduction in under–5 mortality by the year 2015 compared to 1990 levels) and MDG 5 (which called for a three–quarters reduction of maternal mortality) [6]. One of the important reasons for this disappointing result was the failure to implement and scale up evidence–based community–based interventions.

To date, there has been limited attention given to systematically accumulating and analyzing the broad range of evidence regarding the effectiveness of CBPHC in improving MNCH, although excellent summaries of portions of this evidence do exist [1–3,7–17]. In addition, there appears to be a rebirth of global primary health care more generally, especially in light of the upcoming 40^th^ anniversary of the signing of the Declaration of Alma–Ata at the International Conference on Primary Health Care at Alma–Ata, Kazakhstan in 1978, sponsored by the World Health Organization and UNICEF [18]. This article is the first of a series that highlights the findings of a comprehensive review and analysis of this evidence in low– and middle–income countries (LMICs).

## The context

The global primary health care movement began in the 1960s following the recognition that hospitals were not improving the health of the populations they were serving. At that time, a series of surveys of populations served by hospital–oriented Christian medical mission programs around the world demonstrated that the people who had easy access to and used the hospital regularly were no healthier than people who did not [19]. This led to the formation of the Christian Medical Commission (CMC) of the World Council of Churches, which provided a framework and a forum for new thinking about how programs can best improve the health of people in high–mortality, resource–constrained settings. In the 1970s, these discussions involved global health visionaries of their time, including Dame Nita Barrow, Jack Bryant, Carl Taylor, and William Foege, all of whom were members of the CMC, and high–level officials at the World Health Organization (WHO), including Halfdan Mahler, then Director–General, and Ken Newell, Director of Strengthening of Health Services at WHO [20,21]. One of the fruits of these discussions was the seminal WHO publication, *Health by the People* [22]. This book described a number of successful pioneering CBPHC projects around the world and laid the groundwork for the 1978 International Conference on Primary Health Care at Alma–Ata, Kazakhstan and the now renowned Declaration of Alma–Ata, which called for Health for All by the Year 2000 through primary health care [21,23].

Article V of the 1978 Declaration of Alma–Ata states the following [24]:

“Governments have a responsibility for the health of their people that can be fulfilled only by the provision of adequate health and social measures. A main social target of governments, international organizations and the whole world community in the coming decades should be the attainment by all peoples of the world by the year 2000 of a level of health that will permit them to lead a socially and economically productive life. Primary health care is the key to attaining this target as part of development in the spirit of social justice.”

The broad concept of primary health care articulated in this Declaration was much more than the delivery of medical services at primary health care centers. Primary health care, as defined by the Declaration of Alma–Ata, involves providing preventive, promotive, curative, and rehabilitative health care services as close to the community as possible by members of a health team, including community health workers and traditional practitioners, and it broadened the concept even further by calling for primary health care to also address the primary causes of ill–health through inter–sectoral collaboration, community participation, and reduction of inequities.

Over the past three decades since the Declaration of Alma–Ata, major progress has been made in reducing child and maternal mortality throughout the world. The number of children dying before 5 years of age has declined from 18.9 million in 1960 [25] to 5.9 million in 2015 [26] despite the fact that the number of births each year has increased from 96 million in 1960 [25] to 139 million in 2015 [27]. The global under–5 mortality rate has declined from 148 per 1000 live births in 1970 [25] to 43 in 2015 [26]. Over the past 25 years, the global under–5 mortality rate globally has fallen by 53% [26], far less than the 67% required to reach the Millennium Development Goal for 2015. Reductions in maternal mortality have also been important but more gradual. The number of maternal deaths declined from 532 000 in 1990 to 303 000 in 2015 [28], and the global maternal mortality ratio fell by 44% during this period [28], far less than the 75% required to achieve the Millennium Development Goal.

Although evidence about the effectiveness of specific community–based interventions is generally well–documented, evidence about the total range of CBPHC interventions for MNCH, their effectiveness, how these interventions are actually delivered in practice (particularly in combination with other interventions), and the conditions that appear to be important for achieving success are less summarized. This is the heart of what our review is about.

Our review begins with the premises that (1) further strengthening CBPHC by expanding the population coverage of evidence–based interventions has the potential to accelerate progress in ending preventable child and maternal deaths, and (2) CBPHC has the potential for providing an entry point for establishing a more comprehensive primary health care system in resource–constrained settings that can enable health systems to more effectively improve population health and, at the same time, more effectively meet the needs and expectations of local people for medical care.

There is now, more than ever, a need for evaluation of what works and for “systematic sharing of good practices and greater sharing of new information” [29]. As an editorial in *The Lancet* [30] observed:

“Evaluation must now become the top priority in global health. Currently, it is only an afterthought. A massive scale–up in global health investments during the past decade has not been matched by an equal commitment to evaluation…. [Evaluation] will not only sustain interest in global health. It will improve quality of decision making, enhance efficiency, and build capacity for understanding why some programmes work and others do not. Evaluation matters. Evaluation is science.”

This series provides an opportunity to summarize, review and analyze the evidence regarding the effectiveness of CBPHC in improving the health of mothers and their children, to draw conclusions regarding the findings from this review, and to suggest next steps in research, policy and program implementation.

## Background of the review

In the early 1990s, Dr John Wyon (now deceased) and Dr Henry Perry organized panels at the annual meetings of the American Public Health Association (APHA) to highlight the contributions of CBPHC to improving the health of geographically–defined populations. As a result of support and encouragement from the International Health Section at APHA and from APHA staff, a Working Group on CBPHC within the International Health Section was established in 1997. For two decades now, the Working Group has been holding day–long annual workshops on themes related to CBPHC. One of these workshops led to the publication of a book on CBPHC [31]. As the evidence continued to mount regarding the effectiveness of CBPHC in improving health, the Working Group decided that a comprehensive review was needed.

Thus, beginning in 2005, the Working Group created a Task Force for the Review of the Evidence of CBPHC in Improving Child Health, with Henry Perry and Paul Freeman serving as Co–Chairs. What began as a small volunteer effort by Perry and Freeman and others has now, more than a decade later, involved over 150 people and not only APHA but also the World Health Organization, UNICEF, the World Bank, the US Agency for International Development, Future Generations (the NGO where Dr Perry was employed at the outset of the review), and most recently the Gates Foundation.

Following an initial small grant from the World Health Organization in 2006, an Expert Panel was created under the chairmanship of Dr Carl Taylor, then Professor Emeritus of International Health at the Johns Hopkins University (**Table 1**). This group participated in the initial design of the review and then later met face to face at UNICEF Headquarters in 2008 to discuss preliminary findings of the review. After Dr Taylor’s death in 2010, the Panel reconvened under the leadership of Dr Robert Black, Professor of International Health at Johns Hopkins, and has participated in the final set of recommendations that constitute the final article in this series [32].

**Table 1:** Members of the Expert Panel for the Review of the Effectiveness of Community–Based Primary Health Care in Improving Maternal, Neonatal and Child Health

Name	Organizational affiliation	Title	Location	Participated in formalization of guidelines for review 2006	Participated in face–to–face meeting of Panel in 2008	Participated in review of final findings (2016)
Raj Arole	Jamkhed Comprehensive Rural Health Project	Director (now deceased)	Jamkhed, India	X		
Shobha Arole	Jamkhed Comprehensive Rural Health Project	Director	Jamkhed, India			X
Rajiv Bahl	World Health Organization	Medical Officer, Child and Adolescent Health and Development Unit	Geneva, Switzerland	X		
Abhay Bang	Society for Education, Action and Research in Community Health (SEARCH)	Director	Gadchiroli, India	X	X	X
Al Bartlett	United States Agency for International Development	Formerly Senior Advisor for Child Survival, USAID; now retired	Washington, DC, USA	X		
Zulfiqar Bhutta	Centre for Global Child Health, Hospital for Sick Children, Toronto, Canada and Center of Excellence in Women and Child Health, the Aga Khan University, Karachi, Pakistan	Professor	Toronto, Canada and Karachi, Pakistan			X
Robert Black*	Bloomberg School of Public Health, Johns Hopkins University	Professor, Department of International Health	Baltimore, MD, USA	X	X	X
Mushtaque Chowdhury	BRAC	Formerly Dean of the James Grant School of Public Health; currently Deputy Director	Dhaka, Bangladesh			X
Anthony Costello	World Health Organization	Formerly Professor, International Perinatal Care Unit, Institute of Child Health, University College, London; currently Director, Department of Maternal, Newborn, Child and Adolescent Health	Geneva, Switzerland	X		
Dan Kaseje	Tropical Institute of Community Health and Development	Director	Kisumu, Kenya	X	X	X
Betty Kirkwood	London School of Hygiene and Tropical Medicine	Public Health Intervention Research Unit, Professor of Epidemiology and International Health	London, England	X		X
Rudolph Knippenberg	UNICEF	Senior Advisor for Health	New York, NY, USA	X	X	
Nazo Kureshy	United States Agency for International Development	Team Leader, Child Survival and Health Grants Program, Bureau for Global Health	Washington, DC, USA		X	X
Claudio Lanata	Instituto de Investigation Nutricional	Senior Researcher	Lima, Peru	X	X	X
Adetokunbo Lucas	Harvard University	Adjunct Professor of International Health	Ibidan, Nigeria	X	X	
James Phillips	Mailman School of Public Health, Columbia University	Professor	New York, NY, USA	X	X	X
Pang Ruyan	School of Public Health, Peking University	Visiting Professor and formerly National Coordinator for China, WHO Global Survey on Maternal and Perinatal Health	Beijing, China	X	X	
David Sanders	School of Public Health, University of Western Cape	Professor and Dean emeritus	Cape Town, South Africa	X	X	
Agnes Soucat	World Health Organization	Formerly Lead Economist, Human Development, Africa Region of the World Bank and currently Director of Health Systems, Governance and Financing of the World Health Organization	Geneva, Switzerland	X		
Carl Taylor†	Bloomberg School of Public Health, Johns Hopkins University	Professor Emeritus, Department of International Health (now deceased)	Baltimore, MD, USA	X	X	
Mary Taylor	Independent consultant	Formerly Senior Program Officer, Community Health Solutions, the Gates Foundation and currently Independent Senior Technical Expert	South Royalton, Vermont, USA	X	X	X
Cesar Victora	Federal University of Pelotas	Professor of Epidemiology	Pelotas, Brazil	X		X
Zonghan Zhu	Capital Institute of Pediatrics and China Advisory Center for Child Health, Beijing; Chinese Preventive Medicine Association	Professor, Capital Institute of Pediatrics and China Advisory Center for Child Health, Beijing, and Chairman of Child Health, Chinese Preventive Medicine Association	Beijing, China	X	X	X

*Chair of the Panel, 2010 to present.

†Chair of the Panel, 2006–2010.

When the review began in 2006, the focus was exclusively on child health (that is, the health of children in their first 5 years of life). With support from USAID and the Gates Foundation between 2013 and 2016, it became possible to expand the scope of the review to maternal health. Thus, we have now renamed the overall effort a review of the effectiveness of CBPHC in improving MNCH.

## Goals of the review

The goal of this review is to summarize the evidence regarding what can be achieved through community–based approaches to improve MNCH. The health of mothers, neonates and children as a measurable outcome is defined here for our purposes as the level of mortality, serious morbidity, nutritional status, or coverage of proven interventions for mothers, neonates and children in a geographically defined population. The review focuses on interventions and approaches that are carried out beyond the walls of health facilities that serve populations of mothers, neonates and children living in geographically defined areas.

The review consists of an analysis of documents describing research studies, field projects, and programs (collectively referred to in this series as projects) that have assessed the impact of CBPHC on MNCH. Altogether, the findings comprise a comprehensive overview of the global evidence in using CBPHC to improve MNCH. In addition, the review describes the strategies used to deliver community–based interventions and the role of the community and community health workers in implementing these interventions. In addition, the review seeks to understand the context of the projects – where they were implemented and by whom, where the funding came from, for how long, what size of population was served by the project, and what additional contextual factors might have influenced the project outcomes – as well as the methodological quality of the assessment.

The questions which the review seeks to answer are:

- How strong is the evidence that CBPHC can improve MNCH in geographically defined populations and sustain that improvement?
- What specific CBPHC activities improve MNCH?
- What conditions (including those within the local health system) facilitate the effectiveness of CBPHC and what community–based approaches appear to be most effective?
- What characteristics do effective CBPHC activities share?
- What program elements are correlated with improvements in child and maternal health?
- How strong is the evidence that partnerships between communities and health systems are required in order to improve child and maternal health?
- How strong is the evidence that CBPHC can promote equity?
- What general lessons can be drawn from the findings of this review?
- What additional research is needed?
- How can successful community–based approaches for improving MNCH be scaled up to regional and national levels within the context of serious financial and human resource constraints?
- What are the implications for local, national and global health policy, for program implementation, and for donors?

## METHODS

The Task Force and the Expert Panel agreed on the following definition of CBPHC:

CBPHC is a process through which health programs and communities work together to improve health and control disease. CBPHC includes the promotion of key behaviors at the household level as well as the provision of health care and health services outside of health facilities at the community level. CBPHC can (and of course should) connect to existing health services, health programs, and health care provided at static facilities (including health centers and hospitals) and be closely integrated with them.

CBPHC involves improving the health of a geographically defined population through outreach outside of health facilities. CBPHC does not include health care provided at a health facility unless there is community involvement and associated services beyond the facility.

CBPHC also includes multi–sectoral approaches to health improvement beyond the provision of health services per se, including programs that seek to improve (directly or indirectly) education, income, nutrition, living standards, and empowerment.

CBPHC programs may or may not collaborate with governmental or private health care programs; they may be comprehensive in scope, highly selective, or somewhere in between; and they may or may not be part of a program which includes the provision of services at health facilities.

CBPHC includes the following three different types of interventions:

- Health communication with individuals, families and communities;
- Social mobilization and community involvement for planning, delivering, evaluating and using health services; and
- Provision of health care in the community, including preventive services (eg, immunizations) or curative services (eg, community–based treatment of pneumonia).

### Types of assessments of maternal, neonatal and child health interventions qualifying for review

The Task Force sought documents that described community–based programs, projects and research studies that carried out assessments of changes in MNCH indicators in such a way that any changes observed could reasonably be attributed to CBPHC program interventions. At least one of the following outcome indicators was required to be present in order for the assessment to be included in the review.

#### Maternal health

- Change in the population coverage of one or more evidence–based interventions (utilization of antenatal care, delivery by a trained attendant, delivery in a health facility, clean delivery, and postpartum care)
- Change in nutritional status
- Change in the incidence or in the outcome of serious, life–threatening morbidity (such as pre–eclampsia, eclampsia, sepsis, hemorrhage); or,
- Change in mortality.

#### Neonatal and child health

- Change in the population coverage of one or more evidence–based interventions (clean delivery; appropriate care during the neonatal period; appropriate infant and young child feeding, including appropriate breastfeeding; immunizations; vitamin A supplementation; appropriate prevention of malaria with insecticide–treated bed nets and intermittent preventive therapy; appropriate hand washing; appropriate treatment of drinking water, appropriate sanitation; appropriate treatment of pneumonia, diarrhea and malaria;
- Change in nutritional status (as measured by anthropometry, anemia, or assessment of micro–nutrient deficiency);
- Change in the incidence or in the outcome of serious but non–life–threatening morbidity (such as trachoma, which can result in blindness);
- Change in the incidence or in the outcome of serious, life–threatening morbidity (such as pneumonia, diarrhea, malaria, and low–birth weight); or,
- Change in mortality (perinatal, neonatal, infant, 1–4–year, and under–5 mortality);

In addition, the review included an analysis of available documentation concerning the degree to which improvements in child health obtained by CBPHC approaches were equitable.

### Document retrieval

The principal inclusion criteria for the literature review were: (1) a report describing the CBPHC program for a defined geographic population and (2) a description of the findings of an assessment of the project’s effect on maternal, neonatal or child health as defined above. The focus was on the effectiveness of program interventions on the health of all mothers and/or children in a geographically defined area, although in some cases (eg, in studies of maternal–to–child HIV transmission), the focus was on a subset of mothers and their children in a geographically defined area.

**Figure 1:**
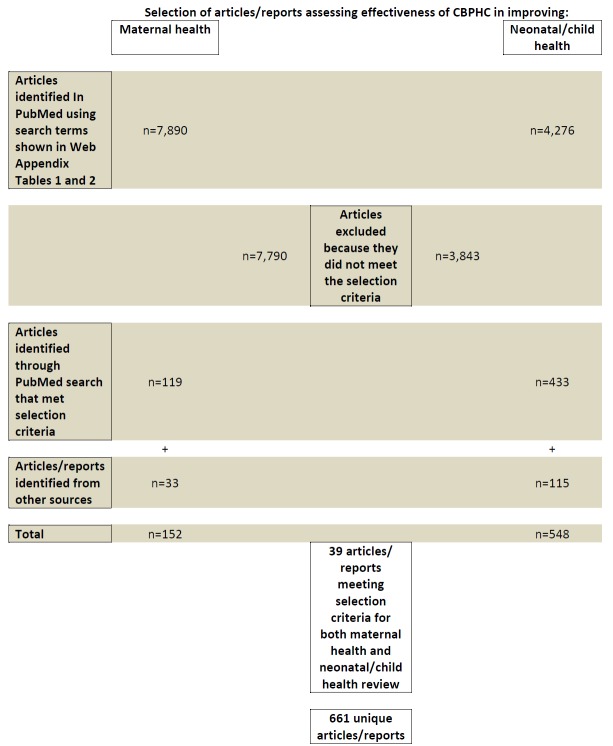
Selection process of assessments of the effectiveness of community-based primary health care (CBPHC).

Key terms for “maternal health,” “child health,” “community health,” and “developing countries” and related terms were identified to create a search query (see Tables S1 and S2 in **Online Supplementary Document(Online Supplementary Document)**). The United States National Library of Medicine’s PubMed database was searched periodically up until 31 December 2015 using these two queries, yielding 7890 articles on maternal health and 4276 articles on neonatal or child health (**Figure 1**). The articles were screened separately by two members of the study team. Assessments of the effectiveness of CBPHC in which the outcomes were improvements in neurological, emotional or psychological development of children were not included unless the reports also included one or more of the other neonatal or child health outcome measures mentioned above.

In addition to the PubMed search, broadcasts were sent out on widely used global health listservs, including those of the Global Health Council, the American Public Health Association, the Collaboration and Resources Group for Child Health (the CORE Group), the World Federation of Public Health Associations, and the Association of Schools of Public Health asking for information about documents, reports, and published articles which might qualify for the review. Finally, the Task Force contacted knowledgeable persons in the field for their suggestions for documents to be included, including members of the Expert Panel. Documents not published in peer–reviewed scientific journals were included if they met the criteria for review, if they provided an adequate description of the intervention, and if they had a satisfactory form of evaluation. A total of 152 assessments met the criteria for the maternal health review and 548 for the neonatal/child health review (**Figure 1**).

Table S3 in **Online Supplementary Document(Online Supplementary Document)** contains a bibliography with the references associated with these 700 assessments. The bibliography also indicates which references were in the maternal health review, in the child health review (and which of these were included in the analyses for neonatal health and child health), and the equity review. There are a number of cases in which a single assessment in our database is derived from more than one document. All of these references are included in the bibliography. Thus, when in **Figure 1** above we refer to the number of articles/reports, there are a small number of cases in which we have combined the various articles/reports associated with a single assessment and counted this as only one assessment.

Of the 33 maternal health assessments and the 115 neonatal/child health assessments included in the review that were not identified through PubMed, most (16 and 80, respectively) were project evaluations of child survival projects funded by the USAID Child Survival and Health Grants Program and implemented by US–based non–governmental organizations. These are listed separately in Table S4 in **Online Supplementary Document(Online Supplementary Document).** Other assessments that were not identified through PubMed were evaluations from other sources, books, or book chapters.

### The document review process

Two data extraction forms were prepared through an iterative process. The extraction form to be used for child health assessments and the form for maternal health assessments were identical except for the interventions carried out. These forms are contained in Appendices S5 and S6 in **Online Supplementary Document(Online Supplementary Document)**. Both forms were developed with the purpose of extracting all possible information available regarding how the interventions were implemented at the community level and what the role of the community was in implementation.

Two independent reviewers each completed a Data Extraction Form for each assessment that qualified for the review. A third reviewer provided quality control and resolved any difference observed in the two reviews, and the final summative review was transferred to an EPI INFO database (version 3.5.4) (Epi Info, US Centers for Disease Control and Prevention, Atlanta, Georgia, USA). The names of the reviewers, many of whom worked on a volunteer basis, are shown in the acknowledgment section; their names and professional titles are contained in Table S7 in **Online Supplementary Document(Online Supplementary Document)**.

### Comment on terminology used

The assessments included in our review were carried out for field studies, projects, and programs that employed one or more CBPHC interventions for improving maternal, neonatal and/or child health. This is a heterogeneous group of assessments in the sense that they range from (1) research reports describing the efficacy of single interventions over a short period of time in a highly supervised and well–supported field setting to (2) assessments of programs which provided a comprehensive array of health and development programs over a long period of time in more typical field setting. When referring to this group of community–level activities as a whole, they should properly be referred to as “research studies/field projects/programs” but for practicality’s sake we will refer to them throughout this series simply as “projects,” and the evaluations of their effectiveness as “assessments.”

### Database description

An electronic database describing 700 assessments of the effectiveness of CBPHC in improving MNCH was queried using EPI INFO version 3.5.4 and STATA version 14 (StatCorp LLC, College Station, Texas, USA). For the purpose of this review, the 39 assessments with both maternal and child health outcomes have been counted as separate assessments in our analysis. Overall, 78.8% of assessments are scientific articles published in peer–reviewed journals, 4.0% are some other type of publication (mostly books or reports not available on the internet), and 12.7% are either from the gray literature (available on the internet) or unpublished project evaluations.

Over three–fourths (78.4%) of the assessments included in our review were carried out in rural settings at least in part, while 16.9% and 11.1% were carried out exclusively in an urban or peri–urban setting, respectively.

Among the 700 assessments in our data set, a small proportion contained data from more than one country. Thus, altogether, 786 country–specific assessments were identified. India, Bangladesh, and Nepal had the largest number of assessments (86, 77, and 47, respectively). 49.0% of the country–specific assessments came from Africa WHO Region, 28.5% from the South–East Asia Region, and 9.7% from the Americas (**Table 2** and Table S8 in **Online Supplementary Document(Online Supplementary Document)**). 8.6% of reports assessed interventions in a single community, 38.1% in a set of communities not encompassing an entire health district (or sub–province), 37.5% at the district (or sub–province) level, 7.5% at the provincial/state level, 3.7% at a national level, and 3.2% at a multinational level.

**Table 2:** Number of assessments of the effectiveness of community–based primary health care in improving maternal, neonatal and child health by region and the countries with the greatest number of assessments

WHO Region	Number	% (n = 786)*	Country	Number	% (n = 786)*
Africa	385	49.0%	India	86	10.9
South–East Asia	224	28.5%	Bangladesh	77	9.8
Americas	76	9.7%	Nepal	47	6.0
Eastern Mediterranean	61	7.8%	Ghana	36	4.6
Western Pacific	37	4.7%	Pakistan	35	4.5
Europe	4	0.5%	Uganda	34	4.3
Total	786*	100.0%	Tanzania	30	3.8
			Ethiopia	28	3.6
			Kenya	27	3.4
			Malawi	19	2.4

*The total number of countries listed here exceeds the number of assessments because some assessments were conducted in multiple countries.

The implementing and facilitating organizations for these projects were primarily private entities (NGOs, universities and research organizations), often working with governments at the national, provincial, or local level (**Table 3**). While communities were — by definition — involved in all of these projects, in only 4.3% of assessments were local communities the only identified implementers. Those who actually implemented projects at the local level were community health workers (CHWs), local community members, research workers, and government health staff.

**Table 3:** Implementers of projects for improving MNCH

	Number	% (n = 700)
Facilitating and/or stakeholder organization:		
State or national government	424	60.6
International NGO	281	40.1
Private organization/university/research organization	254	36.3
Local government	243	34.7
Local NGO	125	17.9
National NGO	85	12.1
Faith–based organization	27	3.9
Implementers at the community level:		
Community health workers (either paid or volunteer)	519	74.1
Research workers only for the project	238	34.0
Ministry of health worker or other government–paid health workers/professionals	304	43.4
Local community members (not trained as a CHW)	200	28.6
Expatriates	33	4.7

*Percentages add up to more than 100% because projects often utilized more than one Implementer.

Half (49.3%) of the assessments are of projects serving 5000 or fewer women and children. 18.2% of the assessments are based on data derived from projects reaching more than 25 000 women and children. 61.9% of the projects had begun since 2000. Almost half (46.3%) of projects were less than 2 years in duration and almost two–thirds (62.9%) were implemented for less than 3 years. Among the neonatal and child health assessments, 51.6% were of only one intervention, and 87.4% were of four or fewer interventions. On the other hand, among the maternal health assessments three–quarters (75.7%) included five or more interventions.

Our review includes 16 assessments of projects that were completed before 1980. The earliest report describes the health impact of an integrated primary health care project in South Africa led by Sidney Kark in the 1940s and published in 1952 [33]. The next earliest report concerns the effectiveness of tetanus toxoid immunization in Columbia, South America, published in 1966 [34].

### Number of assessments completed over time

**Figure 2:**
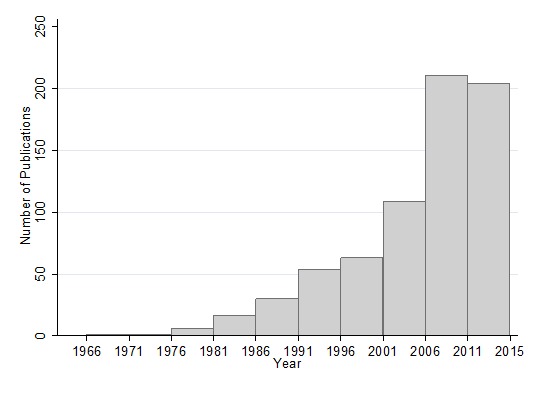
Number of assessments in data set by year of publication (in 5-year intervals).

There has been a rapid growth in the number of assessments published between 1980 and 2015, but particularly in the period 2001–2011, the decade following the establishment of the Millennium Development Goals (MDGs) (**Figure 2**). The surge in publications is present both for maternal and for child/neonatal health studies (data not shown). In the 5 years from 2011 until the end of 2015 when the assessment retrieval ended, there was a slight decline in the number of publications.

### Types of outcomes assessed

We identified a total of 239 outcomes measured in the 700 assessments included in the review: 56 maternal outcomes and 183 neonatal/child outcomes (see Tables S7 and S8 in **Online Supplementary Document(Online Supplementary Document)**). Common maternal health outcomes were changes in: mortality, receipt of antenatal care, attendance at delivery by a skilled provider, facility delivery, care for obstetric emergencies, receipt of nutritional supplements, receipt of tetanus toxoid vaccination, receipt of post–partum family planning, knowledge of safe birth practices, and screening for HIV and other sexually transmitted infections during pregnancy. Common neonatal and child health outcomes were: changes in mortality, serious morbidity, nutritional status, population coverage of healthy behaviors, and changes in the appropriate utilization of health services. In addition, some assessments contained outcome measures that did not qualify for the review but were included with other indicators that did qualify for the review. These include progress in psychomotor development, changes in health–related knowledge among parents and caretakers, quality of community case management of acute childhood illness provided by CHWs, and measures of improvements in health system capacity.

### Types of research methodologies used to assess effectiveness

In the majority (61.0%) of the assessments, a control or comparison group was present. In almost three–fourths (72.5%), pre– and post–intervention data were collected. In 44.6% of the assessments, both data from a comparison group as well as pre– and post–intervention data were present. Randomized controlled assessment designs were present in 33.7% of the assessments. 27.4% of the assessments were uncontrolled before–after assessment designs. Reviewers considered the methodology to be adequate in 89.8% of the assessments, and they considered the assessment quality to be good, high, or exceptional for 88.4% of the assessments.

### Source of financial support for assessments

The United States Agency for International Development (USAID) was far and away the largest source of financial support for the assessments included in our review, contributing to the financial support of one–third (33.4%) of the assessments included in the review. UNICEF supported the next largest number of assessments (15.8%), followed by the World Health Organization (14.2%), the Gates Foundation (10.7%), other UN agencies (7.7%), and the World Bank (6.2%) (**Table 4**). There were numerous other donors that funded a smaller number of assessments. In most (but not all) cases, the donor funded the project as well as the assessment.

**Table 4:** Leading sources of financial support for projects whose assessments were included in the database

Donor	Number of projects/assessments supported	% (n = 700)*
US Agency for International Development	233	33.3
UNICEF	110	15.7
World Health Organization (including the Pan American Health Organization)	99	14.1
The Bill and Melinda Gates Foundation	75	10.7
Other UN agency (eg, UNDP, UNFPA, UNHCR, WFP)	54	7.7
World Bank	43	6.1
Department for International Development (UK)	28	4.0
Canadian International Development Agency (CIDA)	23	3.3
Wellcome Trust	18	2.6

*Multiple funders may have supported a single project/assessment.

### Availability of the database for further analyses and potential further development of the database

We are not aware of any other similar database in existence. It serves as the basis for the subsequent articles in this series [32,35–40]. However, there is an opportunity for more analyses of the database than is reported in this series. Any of the project assessments included in this review are available to be shared with anyone who is interested (contact Henry Perry at hperry2@jhu.edu).

The potential exists for maintaining this as a dynamic database that is regularly updated and publicly available. And, the potential also exists for expanding this database beyond MNCH to include community–based approaches to other global health priorities such as HIV, tuberculosis, malaria, and chronic diseases.

### Limitations of the review

Our review is a comprehensive one, but we make no claim that it is a complete or systematic review. Resources and time constraints prevented screening other electronic databases beyond PubMed for reports that met the inclusion criteria. In addition, the USAID Child Survival and Health Grants program has an archive of more than 400 unpublished child survival project evaluations that meet the criteria for inclusion and are publicly available, but resource and time constraints were such that only one–fifth (80) of these could be included in our review. Since the data analysis and write up portion of this study began, we have identified several additional articles that would have qualified for the review. However, none of these would have changed the overall findings of our review.

This review is limited to documents that describe the impact of project interventions. As is well–known, program failures and serious challenges encountered in program implementation are rarely described in open–access documents or in the scientific literature. This means that a serious publication bias is present and should be recognized. Nonetheless, the inability to document these experiences does not detract from the value of the numerous assessments that have been included in our review that demonstrate effectiveness of CBPHC in improving MNCH.

The degree to which the assessments included in our review represent efficacy assessments as compared to effectiveness assessments is an important issue which we are not able to adequately explore. Efficacy assessments, of course, are carried out for projects that have been implemented under ideal circumstances, when field staff members have optimal training, supervision, resources, and logistical support, and when optimal community engagement has been established. These are conditions that often do not occur in routine settings. Effectiveness assessments, in contrast to efficacy assessments, are carried out under “real world” conditions. Our data extraction form did not collect information on this issue and, in fact, it is often difficult to determine exactly where a project might lie on a continuum between these end points. But it is the case that very few of the assessments in our database were of projects that were implemented without some type of international donor support or technical assistance. Thus, the database is not representative of the effectiveness of current day–to–day practice of CBPHC but rather of what has been achieved in special circumstances in which documentation of effectiveness was undertaken and in which presumably extra efforts had been made to assure the highest quality of implementation possible under the circumstances.

The degree to which these projects improved MNCH depended on many factors: the type(s) and number of interventions implemented, the quality of implementation, and myriad contextual factors. And, of course, the type of outcome indicator(s) employed is important as well. Given the heterogeneity of (1) the types of interventions implemented, (2) the manner in which they were implemented, and (3) the outcome measures used to assess outcomes, it is difficult to make definitive statements about the strength of the evidence, about the magnitude of effect for any specific intervention, or about the effectiveness of one specific approach to implementation compared to another. Rather, the aim of our study is to review the broad scope of evidence related to the effectiveness of CBPHC in improving MNCH and to draw conclusions about the overall effectiveness of CBPHC, the most common strategies used in implementation, and the potential for further strengthening of CBPHC to improve MNCH globally.

It is well–known that the use of family planning, birth spacing, and the reduction of unmet need for family planning all have favorable benefits for MNCH. Furthermore, the evidence on the effectiveness of CBPHC in increasing the coverage of family planning services is extensive. Thus, inclusion of this literature would have made our review more complete, but time and resources were not sufficient to carry this out.

Finally, our review has not included the effectiveness of CBPHC in reducing miscarriages and stillbirths. This topic is an important one but time and resources were not sufficient to carry this out either.

### Subsequent articles in this series

Seven subsequent articles are being published in this series that answer the questions posed by the review. These include: (i) an analysis of the effectiveness of CBPHC in improving maternal health [35], (ii) an analysis of the effectiveness of CBPHC in improving neonatal health [36], (iii) an analysis of the effectiveness of CBPHC in improving child health [37], (iv) an analysis of the effectiveness of CBPHC in promoting equitable improvements in child health [40], (v) the strategies employed by effective CBPHC programs for achieving improvements in MNCH [38], (vi) an analysis of the common characteristics of integrated projects with long–term evidence of effectiveness in improving MNCH [39], and (vii) summary and recommendations of the Expert Panel [32].

## CONCLUSIONS

An extensive database of the evidence regarding the effectiveness of CBPHC in improving MNCH has been assembled. Special attention has been given to how projects were implemented at the community level. The articles that follow in this series describe the findings of analyses of this database along with conclusions and recommendations of an Expert Panel. The aim of this series is to contribute to the formulation of policies and programs that will be useful for ending preventable maternal, neonatal and child deaths and for achieving universal access to care for women and their children by the year 2030 by strengthening CBPHC.
