# Supplementary material for: Comprehensive review of the evidence regarding the effectiveness of community–based primary health care in improving maternal, neonatal and child health: 1. rationale, methods and database description
Source: J Glob Health. 2017 Jun 29;7(1):010901. doi: 10.7189/jogh.07.010901 (PMC5491943; doi:10.7189/jogh.07.010901)
Supplement: Online Supplementary Document [file jogh-07-010901-s001.pdf]

# Online Supplementary Document

**Perry et al. Comprehensive review of the evidence regarding the effectiveness of community–based primary health care in improving maternal, neonatal and child health: 1. rationale, methods and database description**

J Glob Health 2017;7:010901

**Table S1. Maternal health search query used in PUBMED for reports of the effectiveness of community-based primary health care**

| |
|---|
| ((("mortality"[Subheading] OR "mortality"[tiab] OR "mortality"[MeSH Terms] OR "epidemiology"[Subheading] OR "epidemiology"[tiab] OR "morbidity"[tiab] OR "morbidity"[MeSH Terms] OR ("Mortality/prevention and control"[Mesh]) AND "Maternal Mortality"[Mesh] OR "malaria"[MeSH Terms] "hiv infections"[MeSH Terms] OR "hiv infections"[tiab]) AND "Mothers/mortality"[Mesh] OR "Community Networks"[Mesh] OR "Community Health Aides"[Mesh] OR "Community Health Nursing"[Mesh] OR "Community Medicine"[Mesh] OR community network[tiab] OR community networks[tiab] OR community health aide[tiab] OR community health aides[tiab] OR community health nursing[tiab] OR community medicine[tiab] OR community health nurses[tiab] OR community health nurse[tiab] OR community health officers[tiab] OR community health volunteer[tiab] OR community health volunteers[tiab] OR community health worker[tiab] OR community health workers[tiab] OR community IMCI[tiab] OR community-oriented primary care[tiab] OR community oriented primary care[tiab] OR community volunteer[tiab] OR community volunteers[tiab] OR health extension workers[ti ab] OR Paramedical worker[tiab] OR paramedical workers[tiab] OR village development committee[tiab] OR village development committees[tiab] OR village health worker[tiab] OR village health workers[tiab] OR village health volunteer[tiab] OR village health volunteers[tiab] OR community-based nutrition programs[tiab] OR community based nutrition programs[tiab] OR community-based program[tiab] OR community-based programs[tiab] OR community-based programme[tiab] OR community-based programmes[tiab] OR community based programme[tiab] OR community based programmes[tiab] OR community involvement[tiab] OR community participation[tiab] OR community program[tiab] OR community programs[tiab] OR community programme[tiab] OR community programmes[tiab]) AND ("Cote d'Ivoire"[All Fields] OR ("eritrea"[MeSH Terms] OR Eritrea[Text Word]) OR Ethiopia[Text Word] OR ("gambia"[MeSH Terms] OR Gambia[Text Word]) OR ("ghana"[MeSH Terms] OR Ghana[Text Word ]) OR ("guinea"[MeSH Terms] OR Guinea[Text Word]) OR ("guinea-bissau"[MeSH Terms] OR Guinea-Bissau[Text Word]) OR ("haiti"[MeSH Terms] OR Haiti[Text Word]) OR ("india"[MeSH Terms] OR India[Text Word]) OR ("kenya"[MeSH Terms] OR Kenya[Text Word]) OR (Democratic[All Fields] AND ("korea"[TIAB] OR "korea"[MeSH Terms] OR Republic of Korea[Text Word]) OR "Kyrgyz Republic"[All Fields] OR "Lao PDR"[All Fields] OR ("lesotho"[MeSH Terms] OR Lesotho[Text Word]) OR ("liberia"[MeSH Terms] OR Liberia[Text Word]) OR ("madagascar"[MeSH Terms] OR Madagascar[Text Word]) OR ("malawi"[MeSH Terms] OR Malawi[Text Word]) OR ("mali"[MeSH Terms] OR Mali[Text Word]) OR ("mauritania"[MeSH Terms] OR Mauritania[Text Word]) OR ("moldova"[MeSH Terms] OR Moldova[Text Word]) OR ("mongolia"[MeSH Terms] OR Mongolia[Text Word]) OR ("mozambique"[MeSH Terms] OR Mozambique[Text Word]) OR |

("myanmar"[MeSH Terms] OR Myanmar[Text Word]) OR ("nepal"[MeSH Terms] OR Nepal[Text Word]) OR ("nicaragua"[MeSH Terms] OR Nicaragua[Text Word]) OR ("niger"[MeSH Terms] OR Niger[Text Word]) OR ("nigeria"[MeSH Terms] OR Nigeria[Text Word]) OR "North Korea"[All Fields] OR "DPRK"[All Fields] OR ("pakistan"[MeSH Terms] OR Pakistan[Text Word]) OR "Papua New Guinea"[All Fields] OR ("rwanda"[MeSH Terms] OR Rwanda[Text Word]) OR "Sao Tome and Principe"[All Fields] OR ("senegal"[MeSH Terms] OR Senegal[Text Word]) OR "Sierra Leone"[All Fields] OR "Solomon Islands"[All Fields] OR ("somalia"[MeSH Terms] OR Somalia[Text Word]) OR ("sudan"[MeSH Terms] OR Sudan[Text Word]) OR ("tajikistan"[MeSH Terms] OR Tajikistan[Text Word]) OR ("tanzania"[MeSH Terms] OR Tanzania[Text Word]) OR ("east timor"[TIAB] OR "east timor"[MeSH Terms] OR Timor-Leste[Text Word]) OR ("togo"[MeSH Terms] OR Togo[Text Word]) OR ("uganda"[MeSH Terms] OR Uganda[Text Word]) OR ("uzbekistan"[MeSH Terms] OR Uzbekistan[Text Word]) OR ("vietnam"[MeSH Terms] OR Vietnam[Text Word]) OR ("yemen"[MeSH Terms] OR Yemen[Text Word]) OR "Republic of Yemen"[All Fields] OR (("democratic republic of the congo"[TIAB] NOT Medline[SB]) OR "democratic republic of the congo"[MeSH Terms] OR Zaire[Text Word]) OR ("zambia"[MeSH Terms] OR Zambia[Text Word]) OR ("zimbabwe"[MeSH Terms] OR Zimbabwe[Text Word]) OR ("albania"[MeSH Terms] OR Albania[Text Word]) OR ("algeria"[MeSH Terms] OR Algeria[Text Word]) OR ("angola"[MeSH Terms] OR Angola[Text Word]) OR ("armenia"[MeSH Terms] OR Armenia[Text Word]) OR ("azerbaijan"[MeSH Terms] OR Azerbaijan[Text Word]) OR ("belarus"[MeSH Terms] OR Belarus[Text Word]) OR ("bolivia"[MeSH Terms] OR Bolivia[Text Word]) OR "Bosnia and Herzegovina"[All Fields] OR ("brazil"[MeSH Terms] OR Brazil[Text Word]) OR ("bulgaria"[MeSH Terms] OR Bulgaria[Text Word]) OR "Cape Verde"[All Fields] OR ("china"[MeSH Terms] OR China[Text Word]) OR ("colombia"[MeSH Terms] OR Colombia[Text Word]) OR ("cuba"[MeSH Terms] OR Cuba[Text Word]) OR ("djibouti"[MeSH Terms] OR Djibouti[Text Word]) OR "Dominican Republic"[All Fields] OR ("ecuador"[MeSH Terms] OR Ecuador[Text Word]) OR ("egypt"[MeSH Terms] OR Egypt[Text Word]) OR "Arab Republic of Egypt"[All Fields] OR "El Salvador"[All Fields] OR ("fiji"[MeSH Terms] OR Fiji[Text Word]) OR ("georgia"[MeSH Terms] OR "georgia (republic)"[MeSH Terms] OR Georgia[Text Word]) OR ("guatemala"[MeSH Terms] OR Guatemala[Text Word]) OR ("guyana"[MeSH Terms] OR Guyana[Text Word]) OR ("honduras"[MeSH Terms] OR Honduras[Text Word]) OR ("indonesia"[MeSH Terms] OR Indonesia[Text Word]) OR ("iran"[MeSH Terms] OR Iran[Text Word]) OR "Islamic Republic of Iran"[All Fields] OR ("iraq"[MeSH Terms] OR Iraq[Text Word]) OR ("jamaica"[MeSH Terms] OR Jamaica[Text Word]) OR ("jordan"[MeSH Terms] OR Jordan[Text Word]) OR ("kazakhstan"[MeSH Terms] OR Kazakhstan[Text Word]) OR (("micronesia"[TIAB] NOT Medline[SB]) OR "micronesia"[MeSH Terms] OR Kiribati[Text Word]) OR ("macedonia (republic)"[MeSH Terms] OR Macedonia[Text Word]) OR "FYR of Macedonia"[All Fields] OR "Former Yugoslav Republic of Macedonia"[All Fields] OR (("indian ocean islands"[TIAB] NOT Medline[SB]) OR "indian ocean islands"[MeSH Terms] OR Maldives[Text Word]) OR "Marshall Islands"[All Fields] OR ("micronesia"[MeSH Terms] OR Micronesia[Text Word]) OR ("morocco"[MeSH Terms] OR Morocco[Text Word]) OR ("namibia"[MeSH Terms] OR Namibia[Text Word]) OR ("paraguay"[MeSH Terms] OR Paraguay[Text Word]) OR ("peru"[MeSH Terms] OR Peru[Text Word]) OR ("philippines"[MeSH Terms] OR Philippines[Text Word]) OR ("romania"[MeSH Terms] OR Romania[Text Word]) OR ("samoa"[MeSH Terms] OR Samoa[Text Word]) OR "Serbia and Montenegro"[All Fields] OR "Sri Lanka"[All Fields] OR ("suriname"[MeSH Terms] OR Suriname[Text Word]) OR ("swaziland"[MeSH Terms] OR Swaziland[Text Word]) OR "Syrian Arab Republic"[All Fields] OR ("syria"[MeSH Terms] OR Syria[Text Word]) OR ("thailand"[MeSH Terms] OR Thailand[Text Word]) OR ("tonga"[MeSH Terms] OR Tonga[Text Word]) OR ("tunisia"[MeSH Terms] OR Tunisia[Text Word]) OR ("turkmenistan"[MeSH Terms] OR Turkmenistan[Text Word]) OR ("ukraine"[MeSH Terms] OR Ukraine[Text Word]) OR ("vanuatu"[MeSH Terms] OR Vanuatu[Text Word]) OR "West Bank and Gaza"[All Fields] OR "American Samoa"[All Fields] OR "Antigua

and Barbuda"[All Fields] OR ("argentina"[MeSH Terms] OR Argentina[Text Word]) OR ("barbados"[MeSH Terms] OR Barbados[Text Word]) OR ("belize"[MeSH Terms] OR Belize[Text Word]) OR ("botswana"[MeSH Terms] OR Botswana[Text Word]) OR ("chile"[MeSH Terms] OR Chile[Text Word]) OR "Costa Rica"[All Fields] OR ("croatia"[MeSH Terms] OR Croatia[Text Word]) OR "Czech Republic"[All Fields] OR ("dominica"[MeSH Terms] OR Dominica[Text Word]) OR "Equatorial Guinea"[All Fields] OR ("estonia"[MeSH Terms] OR Estonia[Text Word]) OR ("gabon"[MeSH Terms] OR Gabon[Text Word]) OR ("grenada"[MeSH Terms] OR Grenada[Text Word]) OR ("hungary"[MeSH Terms] OR Hungary[Text Word]) OR ("latvia"[MeSH Terms] OR Latvia[Text Word]) OR ("lebanon"[MeSH Terms] OR Lebanon[Text Word]) OR ("libya"[MeSH Terms] OR Libya[Text Word]) OR ("lithuania "[MeSH Terms] OR Lithuania[Text Word]) OR ("malaysia"[MeSH Terms] OR Malaysia[Text Word]) OR ("mauritius"[MeSH Terms] OR Mauritius[Text Word]) OR (("comoros"[TIAB] NOT Medline[SB] ) OR "comoros"[MeSH Terms] OR Mayotte[Text Word]) OR ("mexico"[MeSH Terms] OR Mexico[Text Word]) OR "Northern Mariana Islands"[All Fields] OR ("oman"[MeSH Terms] OR Oman[Text Word]) OR ("palau"[MeSH Terms] OR Palau[Text Word]) OR ("panama"[MeSH Terms] OR Panama[Text Word]) OR ("poland"[MeSH Terms] OR Poland[Text Word]) OR "Russian Federation"[All Fields] OR ("seychelles"[MeSH Terms] OR Seychelles[Text Word]) OR "Slovak Republic"[All Fields] OR "South Africa"[All Fields] OR "St. Kitts and Nevis"[All Fields] OR "St. Lucia"[All Fields] OR "St. Vincent and the Grenadines"[All Fields] OR "Trinidad and Tobago"[All Fields] OR ("turkey"[MeSH Terms] OR Turkey[Text Word]) OR ("uruguay"[MeSH Terms] OR Uruguay[Text Word]) OR ("venezuela"[MeSH Terms] OR Venezuela[Text Word]) OR "developing countries"[All Fields] OR "less developed countries"[All Fields] OR "third-world countries"[All Fields] OR "under-developed countries"[All Fields] OR "poOR countries"[All Fields] OR "less developed countries"[All Fields] OR "under developed countries"[All Fields] OR "less developed nations"[All Fields] OR "third world nations"[All Fields] OR "under developed nations"[All Fields] OR "developing nations"[All Fields] OR "poOR nations"[All Fields] OR "poor economies"[All Fields] OR (third[All Fields] AND world[All Fields] AND economies[All Fields]) OR "developing economies"[All Fields] OR (under[All Fields] AND developed[All] AND economies[All Fields]) OR "less developed economies"[All Fields] OR "myanmar"[MeSH Terms] OR Burma[Text Word]) OR "Czechoslovakia"[All Fields] OR "Democratic Republic of Congo"[All Fields] OR "French Guiana"[All Fields] OR "East Timor"[All Fields] OR ("laos"[MeSH Terms] OR Laos[Text Word]) OR "North Korea"[All Fields] OR "Ivory Coast"[All Fields] OR "Republic of Georgia"[All Fields] OR "Republic of Yemen"[All Fields] OR "Republic of Zaire"[All Fields] OR ("slovakia"[MeSH Terms] OR Slovakia[Text Word]) OR "Soviet Union"[All Fields] OR "suriname"[MeSH Terms] OR Surinam[Text Word] OR ("ussr"[MeSH Terms] OR USSR[Text Word]) OR (West[All Fields] AND ("samoa"[MeSH Terms] OR Samoa[Text Word])) OR ("yugoslavia"[MeSH Terms] OR Yugoslavia[Text Word]) OR ("democratic republic of the congo"[MeSH Terms] OR Zaire[Text Word]) OR ("asia"[MeSH Terms] OR Asia[Text Word]) OR "West Indies"[All Fields] OR ("polynesia"[MeSH Terms] OR Polynesia[Text Word]) OR ("micronesia"[MeSH Terms] OR Micronesia[Text Word]) OR "Middle East"[All Fields] OR ("africa"[MeSH Terms] OR Africa[Text Word]) OR "Latin America"[All Fields] OR "Central America"[All Fields] OR "South America"[All Fields] OR (west indies[tiab] OR "west indies"[MeSH Terms] OR ("caribbean region"[TIAB] NOT Medline[SB]) OR "caribbean region"[MeSH Terms] OR Caribbean[Text Word]) OR (("caribbean region"[TIAB] NOT Medline[SB]) OR "caribbean region"[MeSH Terms]) OR Hispanola[All Fields] OR "Southeast Asia"[All Fields] OR "Sub-Saharan Africa"[All Fields] OR "Eastern Europe"[All Fields] OR Balkans[All Fields] OR "Bangladesh"[MeSH Terms] OR Bangladesh[Text Word]))

**Table S2. Neonatal and child health search query used in PUBMED for reports of the effectiveness of community-based primary health care**

| |
|---|
| ((("mortality"[Subheading] OR "mortality"[tiab] OR "mortality"[MeSH Terms] OR "epidemiology"[Subheading] OR "epidemiology"[tiab] OR "morbidity"[tiab] OR "morbidity"[MeSH Terms] OR child survival[text word] OR "diarrhea"[MeSH Terms] OR Diarrheas[tiab] OR Diarrheas[tiab] OR diarrhoea[tiab] OR diarrhoea[tiab] OR malnutrition[tiab] OR "Malnutrition"[Mesh] OR stunting[tiab] OR wasting[tiab] OR malaria[tiab] OR "malaria"[MeSH Terms] OR "measles"[MeSH Terms] OR measles[tiab] OR "pneumonia"[MeSH Terms] OR pneumonia[tiab] OR "trachoma"[MeSH Terms] OR trachoma[tiab] OR "hiv infections"[MeSH Terms] OR "hiv infections"[tiab]) AND ("child"[MeSH Terms] OR "child"[tiab] OR "child, preschool"[MeSH Terms] OR "preschool child"[tiab] OR "infant"[MeSH Terms] OR infant[tiab] OR "infant, newborn"[MeSH Terms] OR infantile[tiab] OR "newborn"[tiab] OR neonate[tiab] OR neonatal[tiab])) OR ("Infant Nutrition Disorders"[Mesh] OR "Child Nutrition Disorders"[Mesh] OR "Fetal Nutrition Disorders"[Mesh])) AND (("child welfare"[MeSH Terms] OR "Community Networks"[Mesh] OR "Community Health Aides"[Mesh] OR "Community Health Nursing"[Mesh] OR "Community Medicine"[Mesh] OR community network[tiab] OR community networks[tiab] OR community health aide[tiab] OR community health aides[tiab] OR community health nursing[tiab] OR community medicine[tiab] OR community health nurses[tiab] OR community health nurse[tiab] OR community health officers[tiab] OR community health volunteer[tiab] OR community health volunteers[tiab] OR community health worker[tiab] OR community health workers[tiab] OR community IMCI[tiab] OR community-oriented primary care[tiab] OR community oriented primary care[tiab] OR community volunteer[tiab] OR community volunteers[tiab] OR health extension workers[tiab] OR integrated child development services[tiab] OR Paramedical worker[tiab] OR paramedical workers[tiab] OR village development committee[tiab] OR village development committees[tiab] OR village health worker[tiab] OR village health workers[tiab] OR village health volunteer[tiab] OR village health volunteers[tiab] OR community-based nutrition programs[tiab] OR community based nutrition programs[tiab] OR community-based program[tiab] OR community-based programs[tiab] OR community-based programme[tiab] OR community-based programmes[tiab] OR community based programme[tiab] OR community based programmes[tiab] OR community involvement[tiab] OR community participation[tiab] OR community program[tiab] OR community programs[tiab] OR community programme[tiab] OR community programmes[tiab]) AND ("Bangladesh"[MeSH] OR "Bangladesh"[All Fields] OR "Cote d'Ivoire"[All Fields] OR ("eritrea"[MeSH Terms] OR Eritrea[Text Word]) OR Ethiopia[Text Word] OR ("gambia"[MeSH Terms] OR Gambia[Text Word]) OR ("ghana"[MeSH Terms] OR Ghana[Text Word]) OR ("guinea"[MeSH Terms] OR Guinea[Text Word]) OR ("guinea-bissau"[MeSH Terms] OR Guinea-Bissau[Text Word]) OR ("haiti"[MeSH Terms] OR Haiti[Text Word]) OR ("india"[MeSH Terms] OR India[Text Word]) OR ("kenya"[MeSH Terms] OR Kenya[Text Word]) OR (Democratic[All Fields] AND ("korea"[TIAB] OR "korea"[MeSH Terms] OR Republic of Korea[Text Word]) OR "Kyrgyz Republic"[All Fields] OR "Lao PDR"[All Fields] OR ("lesotho"[MeSH Terms] OR Lesotho[Text Word]) OR ("liberia"[MeSH Terms] OR Liberia[Text Word]) OR ("madagascar"[MeSH Terms] OR Madagascar[Text Word]) OR ("malawi"[MeSH Terms] OR Malawi[Text Word]) OR ("mali"[MeSH Terms] OR Mali[Text Word]) OR ("mauritania"[MeSH Terms] OR Mauritania[Text Word]) OR ("moldova"[MeSH Terms] OR Moldova[Text Word]) OR ("mongolia"[MeSH Terms] OR Mongolia[Text Word]) OR ("mozambique"[MeSH Terms] OR Mozambique[Text Word]) OR ("myanmar"[MeSH Terms] OR Myanmar[Text Word]) OR ("nepal"[MeSH Terms] OR Nepal[Text Word]) OR ("nicaragua"[MeSH Terms] OR Nicaragua[Text Word]) OR ("niger"[MeSH Terms] OR Niger[Text Word]) OR ("nigeria"[MeSH Terms] OR Nigeria[Text Word]) OR "North Korea"[All Fields] OR "DPRK"[All Fields] OR ("pakistan"[MeSH Terms] OR Pakistan[Text Word]) OR "Papua New Guinea"[All Fields] OR |

("rwanda"[MeSH Terms] OR Rwanda[Text Word]) OR "Sao Tome and Principe"[All Fields] OR ("senegal"[MeSH Terms] OR Senegal[Text Word]) OR "Sierra Leone"[All Fields] OR "Solomon Islands"[All Fields] OR ("somalia"[MeSH Terms] OR Somalia[Text Word]) OR ("sudan"[MeSH Terms] OR Sudan[Text Word]) OR ("tajikistan"[MeSH Terms] OR Tajikistan[Text Word]) OR ("tanzania"[MeSH Terms] OR Tanzania[Text Word]) OR ("east timor"[TIAB] OR "east timor"[MeSH Terms] OR Timor-Leste[Text Word]) OR ("togo"[MeSH Terms] OR Togo[Text Word]) OR ("uganda"[MeSH Terms] OR Uganda[Text Word]) OR ("uzbekistan"[MeSH Terms] OR Uzbekistan[Text Word]) OR ("vietnam"[MeSH Terms] OR Vietnam[Text Word]) OR ("yemen"[MeSH Terms] OR Yemen[Text Word]) OR "Republic of Yemen"[All Fields] OR (("democratic republic of the congo"[TIAB] NOT Medline[SB]) OR "democratic republic of the congo"[MeSH Terms] OR Zaire[Text Word]) OR ("zambia"[MeSH Terms] OR Zambia[Text Word]) OR ("zimbabwe"[MeSH Terms] OR Zimbabwe[Text Word]) OR ("albania"[MeSH Terms] OR Albania[Text Word]) OR ("algeria"[MeSH Terms] OR Algeria[Text Word]) OR ("angola"[MeSH Terms] OR Angola[Text Word]) OR ("armenia"[MeSH Terms] OR Armenia[Text Word]) OR ("azerbaijan"[MeSH Terms] OR Azerbaijan[Text Word]) OR ("byelarus"[MeSH Terms] OR Belarus[Text Word]) OR ("bolivia"[MeSH Terms] OR Bolivia[Text Word]) OR "Bosnia and Herzegovina"[All Fields] OR ("brazil"[MeSH Terms] OR Brazil[Text Word]) OR ("bulgaria"[MeSH Terms] OR Bulgaria[Text Word]) OR "Cape Verde"[All Fields] OR ("china"[MeSH Terms] OR China[Text Word]) OR ("colombia"[MeSH Terms] OR Colombia[Text Word]) OR ("cuba"[MeSH Terms] OR Cuba[Text Word]) OR ("djibouti"[MeSH Terms] OR Djibouti[Text Word]) OR "Dominican Republic"[All Fields] OR ("ecuador"[MeSH Terms] OR Ecuador[Text Word]) OR ("egypt"[MeSH Terms] OR Egypt[Text Word]) OR "Arab Republic of Egypt"[All Fields] OR "El Salvador"[All Fields] OR ("fiji"[MeSH Terms] OR Fiji[Text Word]) OR ("georgia"[MeSH Terms] OR "georgia (republic)"[MeSH Terms] OR Georgia[Text Word]) OR ("guatemala"[MeSH Terms] OR Guatemala[Text Word]) OR ("guyana"[MeSH Terms] OR Guyana[Text Word]) OR ("honduras"[MeSH Terms] OR Honduras[Text Word]) OR ("indonesia"[MeSH Terms] OR Indonesia[Text Word]) OR ("iran"[MeSH Terms] OR Iran[Text Word]) OR "Islamic Republic of Iran"[All Fields] OR ("iraq"[MeSH Terms] OR Iraq[Text Word]) OR ("jamaica"[MeSH Terms] OR Jamaica[Text Word]) OR ("jordan"[MeSH Terms] OR Jordan[Text Word]) OR ("kazakhstan"[MeSH Terms] OR Kazakhstan[Text Word]) OR (("micronesia"[TIAB] NOT Medline[SB]) OR "micronesia"[MeSH Terms] OR Kiribati[Text Word]) OR ("macedonia (republic)"[MeSH Terms] OR Macedonia[Text Word]) OR "FYR of Macedonia"[All Fields] OR "Former Yugoslav Republic of Macedonia"[All Fields] OR (("indian ocean islands"[TIAB] NOT Medline[SB]) OR "indian ocean islands"[MeSH Terms] OR Maldives[Text Word]) OR "Marshall Islands"[All Fields] OR ("micronesia"[MeSH Terms] OR Micronesia[Text Word]) OR ("morocco"[MeSH Terms] OR Morocco[Text Word]) OR ("namibia"[MeSH Terms] OR Namibia[Text Word]) OR ("paraguay"[MeSH Terms] OR Paraguay[Text Word]) OR ("peru"[MeSH Terms] OR Peru[Text Word]) OR ("philippines"[MeSH Terms] OR Philippines[Text Word]) OR ("romania"[MeSH Terms] OR Romania[Text Word]) OR ("samoa"[MeSH Terms] OR Samoa[Text Word]) OR "Serbia and Montenegro"[All Fields] OR "Sri Lanka"[All Fields] OR ("suriname"[MeSH Terms] OR Suriname[Text Word]) OR ("swaziland"[MeSH Terms] OR Swaziland[Text Word]) OR "Syrian Arab Republic"[All Fields] OR ("syria"[MeSH Terms] OR Syria[Text Word]) OR ("thailand"[MeSH Terms] OR Thailand[Text Word]) OR ("tonga"[MeSH Terms] OR Tonga[Text Word]) OR ("tunisia"[MeSH Terms] OR Tunisia[Text Word]) OR ("turkmenistan"[MeSH Terms] OR Turkmenistan[Text Word]) OR ("ukraine"[MeSH Terms] OR Ukraine[Text Word]) OR ("vanuatu"[MeSH Terms] OR Vanuatu[Text Word]) OR "West Bank and Gaza"[All Fields] OR "American Samoa"[All Fields] OR "Antigua and Barbuda"[All Fields] OR ("argentina"[MeSH Terms] OR Argentina[Text Word]) OR ("barbados"[MeSH Terms] OR Barbados[Text Word]) OR ("belize"[MeSH Terms] OR Belize[Text Word]) OR ("botswana"[MeSH Terms] OR Botswana[Text Word]) OR ("chile"[MeSH Terms] OR Chile[Text Word]) OR "Costa Rica"[All Fields] OR ("croatia"[MeSH Terms] OR Croatia[Text Word]) OR "Czech Republic"[All Fields] OR

("dominica"[MeSH Terms] OR Dominica[Text Word]) OR "Equatorial Guinea"[All Fields] OR ("estonia"[MeSH Terms] OR Estonia[Text Word]) OR ("gabon"[MeSH Terms] OR Gabon[Text Word]) OR ("grenada"[MeSH Terms] OR Grenada[Text Word]) OR ("hungary"[MeSH Terms] OR Hungary[Text Word]) OR ("latvia"[MeSH Terms] OR Latvia[Text Word]) OR ("lebanon"[MeSH Terms] OR Lebanon[Text Word]) OR ("libya"[MeSH Terms] OR Libya[Text Word]) OR ("lithuania "[MeSH Terms] OR Lithuania[Text Word]) OR ("malaysia"[MeSH Terms] OR Malaysia[Text Word]) OR ("mauritius"[MeSH Terms] OR Mauritius[Text Word]) OR (("comoros"[TIAB] NOT Medline[SB]) OR "comoros"[MeSH Terms] OR Mayotte[Text Word]) OR ("mexico"[MeSH Terms] OR Mexico[Text Word]) OR "Northern Mariana Islands"[All Fields] OR ("oman"[MeSH Terms] OR Oman[Text Word]) OR ("palau"[MeSH Terms] OR Palau[Text Word]) OR ("panama"[MeSH Terms] OR Panama[Text Word]) OR ("poland"[MeSH Terms] OR Poland[Text Word]) OR "Russian Federation"[All Fields] OR ("seychelles"[MeSH Terms] OR Seychelles[Text Word]) OR "Slovak Republic"[All Fields] OR "South Africa"[All Fields] OR "St. Kitts and Nevis"[All Fields] OR "St. Lucia"[All Fields] OR "St. Vincent and the Grenadines"[All Fields] OR "Trinidad and Tobago"[All Fields] OR ("turkey"[MeSH Terms] OR Turkey[Text Word]) OR ("uruguay"[MeSH Terms] OR Uruguay[Text Word]) OR ("venezuela"[MeSH Terms] OR Venezuela[Text Word]) OR "developing countries"[All Fields] OR "less developed countries"[All Fields] OR "third-world countries"[All Fields] OR "under-developed countries"[All Fields] OR "poOR countries"[All Fields] OR "less developed countries"[All Fields] OR "under developed countries"[All Fields] OR "less developed nations"[All Fields] OR "third world nations"[All Fields] OR "under developed nations"[All Fields] OR "developing nations"[All Fields] OR "poOR nations"[All Fields] OR "poor economies"[All Fields] OR (third[All Fields] AND world[All Fields] AND economies[All Fields]) OR "developing economies"[All Fields] OR (under[All Fields] AND developed[All] AND economies[All Fields]) OR "less developed economies"[All Fields] OR "myanmar"[MeSH Terms] OR Burma[Text Word]) OR "Czechoslovakia"[All Fields] OR "Democratic Republic of Congo"[All Fields] OR "French Guiana"[All Fields] OR "East Timor"[All Fields] OR ("laos"[MeSH Terms] OR Laos[Text Word]) OR "North Korea"[All Fields] OR "Ivory Coast"[All Fields] OR "Republic of Georgia"[All Fields] OR "Republic of Yemen"[All Fields] OR "Republic of Zaire"[All Fields] OR ("slovakia"[MeSH Terms] OR Slovakia[Text Word]) OR "Soviet Union"[All Fields] OR "suriname"[MeSH Terms] OR Surinam[Text Word] OR ("ussr"[MeSH Terms] OR USSR[Text Word]) OR (West[All Fields] AND ("samoa"[MeSH Terms] OR Samoa[Text Word])) OR ("yugoslavia"[MeSH Terms] OR Yugoslavia[Text Word]) OR ("democratic republic of the congo"[MeSH Terms] OR Zaire[Text Word]) OR ("asia"[MeSH Terms] OR Asia[Text Word]) OR "West Indies"[All Fields] OR ("polynesia"[MeSH Terms] OR Polynesia[Text Word]) OR ("micronesia"[MeSH Terms] OR Micronesia[Text Word]) OR "Middle East"[All Fields] OR ("africa"[MeSH Terms] OR Africa[Text Word]) OR "Latin America"[All Fields] OR "Central America"[All Fields] OR "South America"[All Fields] OR (west indies[tiab] OR "west indies"[MeSH Terms] OR ("caribbean region"[TIAB] NOT Medline[SB]) OR "caribbean region"[MeSH Terms] OR Caribbean[Text Word]) OR (("caribbean region"[TIAB] NOT Medline[SB]) OR "caribbean region"[MeSH Terms]) OR Hispanola[All Fields] OR "Southeast Asia"[All Fields] OR "Sub-Saharan Africa"[All Fields] OR "Eastern Europe"[All Fields] OR Balkans[All Fields]))

## Table S3. Bibliography for the 698 assessments included in the review

| Chronological reference list: Assessments included in the CBPHC review | | | | | | | |
|---|---|---|---|---|---|---|---|
| | | | Type of assessment carried out | | | | |
| | | | | Child health | | | |
| Reference | Year | Reference type | Mater-nal health | Neo-natal health | Health of children 1-59m | Health of 0-59-m-old children | Equity effect |
| Kark, S. L., & Cassel, J. (1952). The Pholela Health Centre; a progress report. S Afr Med J, 26(7), 131-136; concl. doi:http://dx.doi.org/10.2105/ajph.92.11.1743 | 1952 | Journal article | | | | ✓ | |
| Newell, K. W., Duenas Lehmann, A., LeBlanc, D. R., & Garces Osorio, N. (1966). The use of toxoid for the prevention of tetanus neonatorum. Final report of a double-blind controlled field trial. Bull World Health Organ, 35(6), 863-871. | 1966 | Journal article | | ✓ | | ✓ | ✓ |
| Berggren, W. L. (1974). [Control of neonatal tetanus in the rural region of Haiti by using medical auxiliaries]. Bol Oficina Sanit Panam, 77(1), 6-12. | 1974 | Journal article | | | | ✓ | |
| Ram, E. R. (1977). [Integrated health services, the Miraj project in India]. Carnets Enfance, 39, 15-32. | 1977 | Journal article | | | | ✓ | |
| Cunningham, N. (1978). The under fives clinic--what difference does it make? J Trop Pediatr Environ Child Health, 24(6), 239-334. doi:http://dx.doi.org/10.1093/tropej/24.6.237 | 1978 | Journal article | | | | ✓ | |
| Kielmann, A. A., Taylor, C. E., DeSweemer, C., Uberoi, I. S., Takulia, H. S., Masih, N., & Vohra, S. (1978). The Narangwal experiment on interactions of nutrition and infections : II. Morbidity and mortality effects. Indian J Med Res, 68 Suppl, 21-41. | 1978 | Journal article | | | | ✓ | |
| McCord, C., & Kielmann, A. A. (1978). A successful programme for medical auxiliaries treating childhood diarrhoea and pneumonia. Trop Doct, 8(4), 220-225. | 1978 | Journal article | | | | ✓ | |
| Mathur, H. N., Damodar, Sharma, P. N., & Jain, T. P. (1979). The impact of training traditional birth attendants on the utilisation of maternal health services. J Epidemiol Community Health, 33(2), 142-144. doi:http://dx.doi.org/10.1136/jech.33.2.142 | 1979 | Journal article | ✓ | | | | |
| Rahaman, M. M., Aziz, K. M., Patwari, Y., & Munshi, M. H. (1979). Diarrhoeal mortality in two Bangladeshi villages with and without community-based oral rehydration therapy. Lancet, 2(8147), 809-812. doi:http://dx.doi.org/10.1016/s0140-6736(79)92172-x | 1979 | Journal article | | | | ✓ | |
| Berggren, W. L., Ewbank, D. C., & Berggren, G. G. (1981). Reduction of mortality in rural Haiti through a primary-health-care program. N Engl J Med, 304(22), 1324-1330. doi:10.1056/NEJM198105283042203 | 1981 | Journal article | | | | ✓ | |
| Khan, M. U. (1982). Interruption of shigellosis by hand washing. Trans R Soc Trop Med Hyg, 76(2), 164-168. doi:http://dx.doi.org/10.1016/0035-9203(82)90266-8 | 1982 | Journal article | | | ✓ | | |
| Rahman, M., Chen, L. C., Chakraborty, J., Yunus, M., Faruque, A. S., & Chowdhury, A. I. (1982). Use of tetanus toxoid for the prevention of neonatal tetanus. 2. Immunization acceptance among pregnant women in rural Bangladesh. Bull World Health Organ, 60(2), 269-277. | 1982 | Journal article | | ✓ | 4 | | |
| Rahman, S. (1982). The effect of traditional birth attendants and tetanus toxoid in reduction of neo-natal mortality. J Trop Pediatr, 28(4), 163-165. doi:http://dx.doi.org/10.1093/tropej/28.4.163-a | 1982 | Journal article | | | | ✓ | |

| Chronological reference list: Assessments included in the CBPHC review | | | | | | | |
|---|---|---|---|---|---|---|---|
| | | | Type of assessment carried out | | | | |
| | | | | Child health | | | |
| Reference | Year | Reference type | Mater-nal health | Neo-natal health | Health of children 1-59m | Health of 0-59-m-old children | Equity effect |
| Tekce, B. (1982). Oral rehydration therapy: an assessment of mortality effects in rural Egypt. Stud Fam Plann, 13(11), 315-327. doi:http://dx.doi.org/10.2307/1965803 | 1982 | Journal aticle | | | | ✓ | |
| Williamson, N. E. (1982). An attempt to reduce infant and child mortality in Bohol, Philippines. Stud Fam Plann, 13(4), 106-117. doi:http://dx.doi.org/10.2307/1965706 | 1982 | Journal article | | | | ✓ | |
| Berggren, G. G., Berggren, W., Verly, A., Garnier, N., Peterson, W., Ewbank, D., & Dieudonne, W. (1983). Traditional midwives, tetanus immunization, and infant mortality in rural Haiti. Trop Doct, 13(2), 79-87. | 1983 | Journal article | | | | ✓ | |
| Chen, L. C., Rahman, M., D'Souza, S., Chakraborty, J., Sardar, A. M., & Yunus, M. (1983). Mortality impact of an MCH-FP program in Matlab, Bangladesh. Stud Fam Plann, 14(8-9), 199-209. doi:http://dx.doi.org/10.2307/1966412 | 1983 | Journal article | ✓ | | | ✓ | |
| Williamson, N. E., Parado, J. P., & Maturan, E. G. (1983). Providing maternal and child health-family planning services to a large rural population: results of the Bohol Project, Philippines. Am J Public Health, 73(1), 62-71. doi:http://dx.doi.org/10.2105/ajph.73.1.62 | 1983 | Journal article | ✓ | | | | |
| Lamb, W. H., Foord, F. A., Lamb, C. M., & Whitehead, R. G. (1984). Changes in maternal and child mortality rates in three isolated Gambian villages over ten years. Lancet, 2(8408), 912-914. doi:http://dx.doi.org/10.1016/s0140-6736(84)90664-0 | 1984 | Journal article | ✓ | | | ✓ | |
| Berggren, G. G., Hebert, J. R., & Waternaux, C. M. (1985). Comparison of Haitian children in a nutrition intervention programme with children in the Haitian national nutrition survey. Bull World Health Organ, 63(6), 1141-1150. | 1985 | Journal article | | | | ✓ | |
| Kielmann, A. A., Mobarak, A. B., Hammamy, M. T., Gomaa, A. I., Abou-el-Saad, S., Lotfi, R. K., . . . Nagaty, A. (1985). Control of deaths from diarrheal disease in rural communities. I. Design of an intervention study and effects on child mortality. Trop Med Parasitol, 36(4), 191-198. | 1985 | Journal article | | | | ✓ | |
| Kirchhoff, L. V., McClelland, K. E., Do Carmo Pinho, M., Araujo, J. G., De Sousa, M. A., & Guerrant, R. L. (1985). Feasibility and efficacy of in-home water chlorination in rural North-eastern Brazil. J Hyg (Lond), 94(2), 173-180. doi:http://dx.doi.org/10.1017/s0022172400061374 | 1985 | Journal article | | | | ✓ | |
| Moir, J. S., Tulloch, J. L., Vrbova, H., Jolley, D. J., Heywood, P. F., & Alpers, M. P. (1985). The role of voluntary village aides in the control of malaria by presumptive treatment of fever. 1. Selection, training and practice. P N G Med J, 28(4), 257-266. | 1985 | Journal article | | | ✓ | | |
| Mtango, F. D., & Neuvians, D. (1986). Acute respiratory infections in children under five years. Control project in Bagamoyo District, Tanzania. Trans R Soc Trop Med Hyg, 80(6), 851-858. doi:http://dx.doi.org/10.1016/0035-9203(86)90241-5 | 1986 | Journal article | | | ✓ | | |

| Chronological reference list: Assessments included in the CBPHC review | | | | | | | |
|---|---|---|---|---|---|---|---|
| | | | Type of assessment carried out | | | | |
| | | | | Child health | | | |
| Reference | Year | Reference type | Maternal health | Neonatal health | Health of children 1-59m | Health of 0-59-m-old children | Equity effect |
| Sing, K., Mathew, M., & Bhalerao, V. R. (1986). Impact of community-based immunization services. J Postgrad Med, 32(3), 131-133. | 1986 | Journal article | | | | ✓ | |
| Sommer, A., Tarwotjo, I., Djunaedi, E., West, K. P., Jr., Loeden, A. A., Tilden, R., & Mele, L. (1986). Impact of vitamin A supplementation on childhood mortality. A randomised controlled community trial. Lancet, 1(8491), 1169-1173. | 1986 | Journal article | | | | ✓ | |
| Datta, N., Kumar, V., Kumar, L., & Singhi, S. (1987). Application of case management to the control of acute respiratory infections in low-birth-weight infants: a feasibility study. Bull World Health Organ, 65(1), 77-82. | 1987 | Journal article | | ✓ | | | |
| Sharma, V. P. (1987). Community-based malaria control in India. Parasitol Today, 3(7), 222-226. doi:http://dx.doi.org/10.1016/0169-4758(87)90066-4 | 1987 | Journal article | | | | ✓ | |
| Sircar, B. K., Sengupta, P. G., Mondal, S. K., Gupta, D. N., Saha, N. C., Ghosh, S., . . . Pal, S. C. (1987). Effect of handwashing on the incidence of diarrhoea in a Calcutta slum. J Diarrhoeal Dis Res, 5(2), 112-114. | 1987 | Journal article | | | | ✓ | |
| Spencer, H. C., Kaseje, D. C., Mosley, W. H., Sempebwa, E. K., Huong, A. Y., & Roberts, J. M. (1987). Impact on mortality and fertility of a community-based malaria control programme in Saradidi, Kenya. Ann Trop Med Parasitol, 81 Suppl 1, 36-45. | 1987 | Journal article | | ✓ | | | |
| Stanton, B. F., & Clemens, J. D. (1987). An educational intervention for altering water-sanitation behaviors to reduce childhood diarrhea in urban Bangladesh. II. A randomized trial to assess the impact of the intervention on hygienic behaviors and rates of diarrhea. Am J Epidemiol, 125(2), 292-301. doi:http://dx.doi.org/10.1016/0277-9536(87)90054-2 | 1987 | Journal article | | | ✓ | | |
| Yach, D., Hoogendoorn, L., & Von Schirnding, Y. E. (1987). Village health workers are able to teach mothers how to safely prepare sugar/salt solutions. Paediatr Perinat Epidemiol, 1(2), 153-161. doi:http://dx.doi.org/10.1111/j.1365-3016.1987.tb00105.x | 1987 | Journal article | | | ✓ | | |
| Koenig, M. A., Fauveau, V., Chowdhury, A. I., Chakraborty, J., & Khan, M. A. (1988). Maternal mortality in Matlab, Bangladesh: 1976-85. Stud Fam Plann, 19(2), 69-80. doi:http://dx.doi.org/10.2307/1966492 | 1988 | Journal article | ✓ | | | | |
| Muhilal, Permeisih, D., Idjradinata, Y. R., Muherdiyantiningsih, & Karyadi, D. (1988). Vitamin A-fortified monosodium glutamate and health, growth, and survival of children: a controlled field trial. Am J Clin Nutr, 48(5), 1271-1276. | 1988 | Journal article | | | | ✓ | |
| Snow, R. W., Lindsay, S. W., Hayes, R. J., & Greenwood, B. M. (1988). Permethrin-treated bed nets (mosquito nets) prevent malaria in Gambian children. Trans R Soc Trop Med Hyg, 82(6), 838-842. doi:http://dx.doi.org/10.1016/0035-9203(88)90011-9 | 1988 | Journal article | | | ✓ | | |
| Snow, R. W., Rowan, K. M., Lindsay, S. W., & Greenwood, B. M. (1988). A trial of bed nets | 1988 | Journal article | | | ✓ | | |

| Chronological reference list: Assessments included in the CBPHC review | | | | | | | |
|---|---|---|---|---|---|---|---|
| | | | Type of assessment carried out | | | | |
| | | | | Child health | | | |
| Reference | Year | Reference type | Mater-nal health | Neo-natal health | Health of children 1-59m | Health of 0-59-m-old children | Equity effect |
| (mosquito nets) as a malaria control strategy in a rural area of The Gambia, West Africa. Trans R Soc Trop Med Hyg, 82(2), 212-215. doi:http://dx.doi.org/10.1016/0035-9203(88)90414-2 | | | | | | | |
| Bentley, C. (1989). Primary health care in northwestern Somalia: a case study. Soc Sci Med, 28(10), 1019-1030. doi:http://dx.doi.org/10.1016/0277-9536(89)90384-5 | 1989 | Journal article | | | | ✓ | |
| Deming, M. S., Gayibor, A., Murphy, K., Jones, T. S., & Karsa, T. (1989). Home treatment of febrile children with antimalarial drugs in Togo. Bull World Health Organ, 67(6), 695-700. | 1989 | Journal article | | | | ✓ | |
| Doi, H., Kaneko, A., Panjaitan, W., & Ishii, A. (1989). Chemotherapeutic malaria control operation by single dose of Fansidar plus primaquine in North Sumatra, Indonesia. Southeast Asian J Trop Med Public Health, 20(3), 341-349. | 1989 | Journal article | | | | ✓ | |
| Han, A. M., & Hlaing, T. (1989). Prevention of diarrhoea and dysentery by hand washing. Trans R Soc Trop Med Hyg, 83, 128-131. doi:http://dx.doi.org/10.1016/0035-9203(89)90737-2 | 1989 | Journal article | | | ✓ | | |
| Matomora, M. K. (1989). A people-centered approach to primary health care implementation in Mvumi, Tanzania. Soc Sci Med, 28(10), 1031-1037. doi:http://dx.doi.org/10.1016/0277-9536(89)90385-7 | 1989 | Journal article | | | | ✓ | |
| Pandey, M. R., Sharma, P. R., Gubhaju, B. B., Shakya, G. M., Neupane, R. P., Gautam, A., & Shrestha, I. B. (1989). Impact of a pilot acute respiratory infection (ARI) control programme in a rural community of the hill region of Nepal. Ann Trop Paediatr, 9(4), 212-220. doi:http://dx.doi.org/10.1080/02724936.1989.11748635 | 1989 | Journal article | | | ✓ | | |
| Tandon, B. N. (1989). Nutritional interventions through primary health care: impact of the ICDS projects in India. Bull World Health Organ, 67(1), 77-80. | 1989 | Journal article | | | | ✓ | |
| Allen, S. J., Snow, R. W., Menon, A., & Greenwood, B. M. (1990). Compliance with malaria chemoprophylaxis over a five-year period among children in a rural area of The Gambia. J Trop Med Hyg, 93(5), 313-322. | 1990 | Journal article | | | | ✓ | |
| Aziz, K. M., Hoque, B. A., Hasan, K. Z., Patwary, M. Y., Huttly, S. R., Rahaman, M. M., & Feachem, R. G. (1990). Reduction in diarrhoeal diseases in children in rural Bangladesh by environmental and behavioural modifications. Trans R Soc Trop Med Hyg, 84(3), 433-438. doi:http://dx.doi.org/10.1016/0035-9203(90)90353-g | 1990 | Journal article | | | | ✓ | |
| Bang, A. T., Bang, R. A., Tale, O., Sontakke, P., Solanki, J., Wargantiwar, R., & Kelzarkar, P. (1990). Reduction in pneumonia mortality and total childhood mortality by means of community-based intervention trial in Gadchiroli, India. Lancet, 336(8709), 201-206. doi:http://dx.doi.org/10.1016/0140-6736(90)91733-q | 1990 | Journal article | | | | ✓ | |

## Chronological reference list: Assessments included in the CBPHC review

| | | | Type of assessment carried out | | | | |
|---|---|---|---|---|---|---|---|
| | | | | Child health | | | |
| Reference | Year | Reference type | Mater-nal health | Neo-natal health | Health of children 1-59m | Health of 0-59-m-old children | Equity effect |
| el-Rafie, M., Hassouna, W. A., Hirschhorn, N., Loza, S., Miller, P., Nagaty, A., . . . Riyad, S. (1990). Effect of diarrhoeal disease control on infant and childhood mortality in Egypt. Report from the National Control of Diarrheal Diseases Project. Lancet, 335(8685), 334-338. doi:http://dx.doi.org/10.1016/0140-6736(90)90616-d | 1990 | Journal article | | ✓ | | | |
| Fauveau, V., Wojtyniak, B., Chakraborty, J., Sarder, A. M., & Briend, A. (1990). The effect of maternal and child health and family planning services on mortality: is prevention enough? BMJ, 301(6743), 103-107. doi:http://dx.doi.org/10.1136/bmj.301.6743.103 | 1990 | Journal article | ✓ | | | ✓ | |
| Greenwood, B. M., Bradley, A. K., Byass, P., Greenwood, A. M., Menon, A., Snow, R. W., . . . Hatib-N'Jie, A. B. (1990). Evaluation of a primary health care programme in The Gambia. II. Its impact on mortality and morbidity in young children. J Trop Med Hyg, 93(2), 87-97. | 1990 | Journal article | | | | ✓ | |
| Holt, E. A., Boulos, R., Halsey, N. A., Boulos, L. M., & Boulos, C. (1990). Childhood survival in Haiti: protective effect of measles vaccination. Pediatrics, 85(2), 188-194. | 1990 | Journal article | | | | ✓ | |
| Khan, A. J., Khan, J. A., Akbar, M., & Addiss, D. G. (1990). Acute respiratory infections in children: a case management intervention in Abbottabad District, Pakistan. Bull World Health Organ, 68(5), 577-585. | 1990 | Journal article | | | ✓ | | |
| Rahmathullah, L., Underwood, B. A., Thulasiraj, R. D., Milton, R. C., Ramaswamy, K., Rahmathullah, R., & Babu, G. (1990). Reduced mortality among children in southern India receiving a small weekly dose of vitamin A. N Engl J Med, 323(14), 929-935. doi:10.1056/NEJM199010043231401 | 1990 | Journal article | | | | ✓ | |
| Vijayaraghavan, K., Radhaiah, G., Prakasam, B. S., Sarma, K. V., & Reddy, V. (1990). Effect of massive dose vitamin A on morbidity and mortality in Indian children. Lancet, 336(8727), 1342-1345. doi:http://dx.doi.org/10.1016/0140-6736(90)92895-o | 1990 | Journal article | | | | ✓ | |
| Alonso, P. L., Lindsay, S. W., Armstrong, J. R., Conteh, M., Hill, A. G., David, P. H., . . . et al. (1991). The effect of insecticide-treated bed nets on mortality of Gambian children. Lancet, 337(8756), 1499-1502. doi:http://dx.doi.org/10.1016/0140-6736(91)93194-e | 1991 | Journal Article | | | ✓ | | |
| Alto, W. A., Albu, R. E., & Irabo, G. (1991). An alternative to unattended delivery--a training programme for village midwives in Papua New Guinea. Soc Sci Med, 32(5), 613-618. doi:http://dx.doi.org/10.1016/0277-9536(91)90296-o | 1991 | Journal article | ✓ | | | | |
| Fauveau, V., Stewart, K., Khan, S. A., & Chakraborty, J. (1991). Effect on mortality of community-based maternity-care programme in rural Bangladesh. Lancet, 338(8776), 1183-1186. doi:http://dx.doi.org/10.1016/0140-6736(91)92041-y | 1991 | Journal article | ✓ | | | | |
| Koenig, M. A., Fauveau, V., & Wojtyniak, B. (1991). Mortality reductions from health interventions: The case of immunization in Bangladesh. Population and | 1991 | Journal article | | | | ✓ | |

| Chronological reference list: Assessments included in the CBPHC review | | | | | | | | |
|---|---|---|---|---|---|---|---|---|
| | | | Type of assessment carried out | | | | | |
| | | | | Child health | | | | |
| Reference | Year | Reference type | Mater-nal health | Neo-natal health | Health of children 1-59m | Health of 0-59-m-old children | Equity effect |
| Development Review, 17(1), 87-104. doi:http://dx.doi.org/10.2307/1972353 | | | | | | | |
| Pandey, M. R., Daulaire, N. M., Starbuck, E. S., Houston, R. M., & McPherson, K. (1991). Reduction in total under-five mortality in western Nepal through community-based antimicrobial treatment of pneumonia. Lancet, 338(8773), 993-997. doi:http://dx.doi.org/10.1016/0140-6736(91)91847-n | 1991 | Journal article | | | ✓ | | |
| Velema, J. P., Alihonou, E. M., Gandaho, T., & Hounye, F. H. (1991). Childhood mortality among users and non-users of primary health care in a rural west African community. Int J Epidemiol, 20(2), 474-479. doi:http://dx.doi.org/10.1093/ije/20.2.474 | 1991 | Journal article | | | | ✓ | |
| West, K. P., Jr., Pokhrel, R. P., Katz, J., LeClerq, S. C., Khatry, S. K., Shrestha, S. R., . . . Sommer, A. (1991). Efficacy of vitamin A in reducing preschool child mortality in Nepal. Lancet, 338(8759), 67-71. doi:http://dx.doi.org/10.1016/0140-6736(91)90070-6 | 1991 | Journal article | | | | ✓ | |
| Daulaire, N. M., Starbuck, E. S., Houston, R. M., Church, M. S., Stukel, T. A., & Pandey, M. R. (1992). Childhood mortality after a high dose of vitamin A in a high risk population. BMJ, 304(6821), 207-210. doi:http://dx.doi.org/10.1136/bmj.304.6821.207 | 1992 | Journal article | | ✓ | ✓ | | |
| Fauveau, V., Stewart, M. K., Chakraborty, J., & Khan, S. A. (1992). Impact on mortality of a community-based programme to control acute lower respiratory tract infections. Bull World Health Organ, 70(1), 109-116. | 1992 | Journal article | | | ✓ | | |
| Herrera, M. G., Nestel, P., el Amin, A., Fawzi, W. W., Mohamed, K. A., & Weld, L. (1992). Vitamin A supplementation and child survival. Lancet, 340(8814), 267-271. doi:http://dx.doi.org/10.1016/0140-6736(92)92357-l | 1992 | Journal article | | | | ✓ | |
| Ibrahim, S. A., Omer, M. I., Amin, I. K., Babiker, A. G., & Rushwan, H. (1992). The role of the village midwife in detection of high risk pregnancies and newborns. Int J Gynaecol Obstet, 39(2), 117-122. doi:http://dx.doi.org/10.1016/0020-7292(92)90907-z | 1992 | Journal article | ✓ | | | | |
| Tumwine, J. K., & Mackenzie, S. (1992). Child survival in a rural area in Zimbabwe: are we winning? Cent Afr J Med, 38(1), 30-36. | 1992 | Journal article | | | | ✓ | |
| Agarwal, D. K., Bhatia, B. D., & Agarwal, K. N. (1993). Simple approach to acute respiratory infection in rural under five children. Indian Pediatr, 30(5), 629-635. | 1993 | Journal article | | | ✓ | | |
| Ahmed, N. U., Zeitlin, M. F., Beiser, A. S., Super, C. M., & Gershoff, S. N. (1993). A longitudinal study of the impact of behavioural change intervention on cleanliness, diarrhoeal morbidity and growth of children in rural Bangladesh. Soc Sci Med, 37(2), 159-171. doi:http://dx.doi.org/10.1016/0277-9536(93)90452-a | 1993 | Journal article | | | | ✓ | ✓ |
| Bang, A. T., Bang, R. A., Morankar, V. P., Sontakke, P. G., & Solanki, J. M. (1993). Pneumonia in neonates: can it be managed in the community? (Vol. 68). | 1993 | Journal article | | | | ✓ | |

| Chronological reference list: Assessments included in the CBPHC review | | | | | | | | |
|---|---|---|---|---|---|---|---|---|
| | | | Type of assessment carried out | | | | | |
| | | | | Child health | | | | |
| Reference | Year | Reference type | Mater-nal health | Neo-natal health | Health of children 1-59m | Health of 0-59-m-old children | Equity effect |
| Becker, S. R., Diop, F., & Thornton, J. N. (1993). Infant and child mortality in two counties of Liberia: results of a survey in 1988 and trends since 1984. Int J Epidemiol, 22 Suppl 1, S56-63. doi:http://dx.doi.org/10.1093/ije/22.supplement_1.s56 | 1993 | Journal article | | | | ✓ | |
| Chahnazarian, A., Ewbank, D. C., Makani, B., & Ekouevi, K. (1993). Impact of selective primary care on childhood mortality in a rural health zone of Zaire. Int J Epidemiol, 22 Suppl 1, S32-41. doi:http://dx.doi.org/10.1093/ije/22.supplement_1.s32 | 1993 | Journal article | | | | ✓ | |
| Ewbank, D. C. (1993). Impact of health programmes on child mortality in Africa: evidence from Zaire and Liberia. Int J Epidemiol, 22 Suppl 1, S64-72. doi:http://dx.doi.org/10.1093/ije/22.supplement_1.s64 | 1993 | Journal article | | | | ✓ | |
| Foster, S. O., Spiegel, R. A., Mokdad, A., Yeanon, S., Becker, S. R., Thornton, J. N., & Galakpai, M. K. (1993). Immunization, oral rehydration therapy and malaria chemotherapy among children under 5 in Bomi and Grand Cape Mount counties, Liberia, 1984 and 1988. Int J Epidemiol, 22 Suppl 1, S50-55. doi:http://dx.doi.org/10.1093/ije/22.supplement_1.s50 | 1993 | Journal article | | | | ✓ | |
| Husein, K., Adeyi, O., Bryant, J., & Cara, N. B. (1993). Developing a primary health care management information system that supports the pursuit of equity, effectiveness and affordability. Soc Sci Med, 36(5), 585-596. doi:http://dx.doi.org/10.1016/0277-9536(93)90055-9 | 1993 | Journal article | | | | ✓ | |
| Neutzling, M. B., Vieira, M. F., Cesar, J. A., Gigante, D. P., Martins, E. B., & Facchini, L. A. (1993). [Measuring the impact of promoting breastfeeding in primary health care services in Pelotas, Rio Grande do Sul, Brazil]. Cad Saude Publica, 9(2), 149-154. doi:/S0102-311X1993000200005 | 1993 | Journal article | | ✓ | | | |
| Shah, P. M., Selwyn, B. J., Shah, K., & Kumar, V. (1993). Evaluation of the home-based maternal record: a WHO collaborative study. Bull World Health Organ, 71(5), 535-548. | 1993 | Journal article | ✓ | | | | |
| Taylor, W. R., Chahnazarian, A., Weinman, J., Wernette, M., Roy, J., Pebley, A. R., . . . Ma-Disu, M. (1993). Mortality and use of health services surveys in rural Zaire. Int J Epidemiol, 22 Suppl 1, S15-19. doi:http://dx.doi.org/10.1093/ije/22.supplement_1.s15 | 1993 | Journal article | | | | ✓ | |
| Team, G. V. S. (1993). Vitamin A supplementation in northern Ghana: effects on clinic attendances, hospital admissions, and child mortality. Ghana VAST Study Team. Lancet, 342(8862), 7-12. doi:http://dx.doi.org/10.1016/0140-6736(93)91879-q | 1993 | Journal article | | | | ✓ | |
| Vernon, A. A., Taylor, W. R., Biey, A., Mundeke, K. M., Chahnazarian, A., Habicht, H., . . . Makani, B. (1993). Changes in use of health services in a rural health zone | 1993 | Journal article | ✓ | | | | |

| Chronological reference list: Assessments included in the CBPHC review | | | | | | | |
|---|---|---|---|---|---|---|---|
| | | | Type of assessment carried out | | | | |
| | | | | Child health | | | |
| Reference | Year | Reference type | Mater-nal health | Neo-natal health | Health of children 1-59m | Health of 0-59-m-old children | Equity effect |
| in Zaire. Int J Epidemiol, 22 Suppl 1, S20-31. doi:http://dx.doi.org/10.1093/ije/22.supplement_1.s20 | | | | | | | |
| Wilson, J. M., & Chandler, G. N. (1993). Sustained improvements in hygiene behaviour amongst village women in Lombok, Indonesia. Trans R Soc Trop Med Hyg, 87(6), 615-616. doi:http://dx.doi.org/10.1016/0035-9203(93)90260-w | 1993 | Journal article | | | | ✓ | |
| Arole, M., & Arole, R. (1994). Jamkhed: A comprehensive rural health project. Hong Kong: The Macmillan Press. | 1994 | Book | | | | ✓ | |
| Bang, A. T., Bang, R. A., & Sontakke, P. G. (1994). Management of childhood pneumonia by traditional birth attendants. The SEARCH Team. Bull World Health Organ, 72(6), 897-905. | 1994 | Journal article | | ✓ | ✓ | | |
| Bohler, E. (1994). Has primary health care reduced infant mortality in east Bhutan? The effects of primary health care and birth spacing on infant and child mortality patterns in east Bhutan. J Trop Pediatr, 40(5), 256-260. doi:http://dx.doi.org/10.1093/tropej/40.5.256 | 1994 | Journal article | | | | ✓ | |
| Canner, J., Brown, J., Barrows, J., & International Eye Foundation/Malawi. (1994, October 2-7, 1994). Reduction in Diarrheal mortality due to Vitamin A supplementation in Chikwawa district, Malawi. Paper presented at the Community Impact of PVO Child Survival Efforts: 1985-1994, Bangalore, Karnataka, India. | 1994 | Conference proceedings | | | | ✓ | |
| Dearden, K., Khan, N., & Save the Children/ Bangladesh. (1994, October 2-7, 1994). Do women's saving and credit programs affect fertility and health?: A case study from Bangladesh. Paper presented at the Community Impact of PVO Child Survival Efforts: 1985-1994, Bangalore, Karnataka, India. | 1994 | Conference proceedings | | | | ✓ | |
| Dubuisson, S. E., Ludzen, S., Zayan, A., Swedberg, E., & Save the Children/Hait. (1994). Impact of sustainable behavior change on the nutritional status of children. Paper presented at the Community Impact of PVO Child Survival Efforts: 1985-1994, Bangalore, Karnataka, India. | 1994 | Conference proceedings | | | | ✓ | |
| Edwards, L., & World Vision/India. (1994, Oct 2-7, 1994). Impact of PVO child survival interventions on the health of mother and child in 54 trival villages of India. Paper presented at the Community Impact of PVO Child Survival Efforts: 1985-1994, Bangalore, Karnataka, India. | 1994 | Conference proceedings | | | | ✓ | |
| Haggerty, P. A., Muladi, K., Kirkwood, B. R., Ashworth, A., & Manunebo, M. (1994). Community-based hygiene education to reduce diarrhoeal disease in rural Zaire: impact of the intervention on diarrhoeal morbidity. Int J Epidemiol, 23(5), 1050-1059. doi:http://dx.doi.org/10.1093/ije/23.5.1050 | 1994 | Journal article | | | ✓ | | |
| Kaye, K., Khan, N. H., Hossain, A., & Save the Children/Bangladesh. (1994, Oct 2-7, 1994). Effect of a nutrition education program on the weight of younger | 1994 | Conference proceedings | | | | ✓ | |

| Chronological reference list: Assessments included in the CBPHC review | | | | | | | |
|---|---|---|---|---|---|---|---|
| | | | Type of assessment carried out | | | | |
| | | | | Child health | | | |
| Reference | Year | Reference type | Mater-nal health | Neo-natal health | Health of children 1-59m | Health of 0-59-m-old children | Equity effect |
| siblings of malnourished children in Bangladesh. Paper presented at the Community Impact of PVO Child Survival Efforts: 1985-1994, Bangalore, Karnataka, India. | | | | | | | |
| Marfin, A. A., Moore, J., Collins, C., Biellik, R., Kattel, U., Toole, M. J., & Moore, P. S. (1994). Infectious disease surveillance during emergency relief to Bhutanese refugees in Nepal. JAMA, 272(5), 377-381. doi:http://dx.doi.org/10.1001/jama.272.5.377 | 1994 | Journal article | | | | ✓ | |
| Olupona, O. G., & World Vision/Nigeria. (1994, Oct 2 - 7, 1994). The impact of a child survival project on the health and nutrition of mothers and children in a rural nigerian local government area. Paper presented at the Community Impact of PVO Child Survival Efforts: 1985 - 1994, Bangalore, Karnataka, India. | 1994 | Conference proceedings | | | | ✓ | |
| Robbins, A., & Food for the Hungry International/Bolivia. (1994). Community impact of PVO child survival project in Bolivian Altipiano. Paper presented at the Community impact of PVO child survival efforts: 1985-1994, Bangalore, Karnataka, India. | 1994 | Conference proceedings | | | | ✓ | |
| Robinson, S. J., & Project Concern International/ Indonesia, P. C. (1994). Innovations for Increasing Immunization Coverage. Paper presented at the Community Impact of PVO Child Survial Efforts: 1985 - 1994, Bangalore, Karnataka, India, October 2 -7, 1994. | 1994 | Conference proceedings | | | | ✓ | |
| Thiam, L., & World Vision/Senegal (1994, Oct 2-7, 1994). Impact of the Thies CSP on the Health Knowledge and Practices of Mothers Living in the Sub-district of Niakhene (Thies region). Paper presented at the Community Impact of PVO Child Survival Efforts: 1995 - 1994, Bangalore, Karnataka, India. | 1994 | Conference proceedings | ✓ | ✓ | | | |
| Afari, E. A., Nkrumah, F. K., Nakana, T., Sakatoku, H., Hori, H., & Binka, F. (1995). Impact of primary health care on child morbidiy and mortality. Central African Journal of Medicine, 41(5), 148-153. | 1995 | Journal article | ✓ | | | ✓ | |
| Alisjahbana, A., Williams, C., Dharmayanti, R., Hermawan, D., Kwast, B. E., & Koblinsky, M. (1995). An integrated village maternity service to improve referral patterns in a rural area in West-Java. Int J Gynaecol Obstet, 48 Suppl, S83-94. doi:http://dx.doi.org/10.1016/0020-7292(95)02323-5 | 1995 | Journal article | ✓ | | | ✓ | |
| Curtale, F., Siwakoti, B., Lagrosa, C., LaRaja, M., & Guerra, R. (1995). Improving skills and utilization of community health volunteers in Nepal. Soc Sci Med, 40(8), 1117-1125. doi:http://dx.doi.org/10.1016/0277-9536(94)00172-p | 1995 | Journal article | | | | ✓ | |
| D'Alessandro, U., Olaleye, B. O., McGuire, W., Langerock, P., Bennett, S., Aikins, M. K., . . . Greenwood, B. M. (1995). Mortality and morbidity from malaria in Gambian children after introduction of an impregnated bednet programme. Lancet, 345(8948), 479-483. doi:http://dx.doi.org/10.1016/s0140-6736(95)90582-0 | 1995 | Journal article | | | ✓ | | |

| Chronological reference list: Assessments included in the CBPHC review | | | | | | | |
|---|---|---|---|---|---|---|---|
| | | | Type of assessment carried out | | | | |
| | | | | Child health | | | |
| Reference | Year | Reference type | Maternal health | Neonatal health | Health of children 1-59m | Health of 0-59-m-old children | Equity effect |
| Foord, F. (1995). Gambia: evaluation of the mobile health care service in West Kiang district. World Health Stat Q, 48(1), 18-22. | 1995 | Journal article | ✓ | | | | |
| Linkins, R. W., Mansour, E., Wassif, O., Hassan, M. H., & Patriarca, P. A. (1995). Evaluation of house-to-house versus fixed-site oral poliovirus vaccine delivery strategies in a mass immunization campaign in Egypt. Bull World Health Organ, 73(5), 589-595. | 1995 | Journal article | | | | ✓ | |
| Magnani, R. J. (1995). Strengthening MCH/FP at the grassroots level: People's Republic of China. United Nations Population Fund. | 1995 | Unpublished work | ✓ | | | | |
| Melville, B., Fidler, T., Mehan, D., Bernard, E., & Mullings, J. (1995). Growth monitoring: the role of community health volunteers. Public Health, 109(2), 111-116. doi:http://dx.doi.org/10.1016/s0033-3506(05)80004-6 | 1995 | Journal article | | | | ✓ | |
| Miller, P., & Hirschhorn, N. (1995). The effect of a national control of diarrheal diseases program on mortality: the case of Egypt. Soc Sci Med, 40(10), S1-S30. doi:http://dx.doi.org/10.1016/0277-9536(95)00001-n | 1995 | Journal article | | | | ✓ | |
| Zhenxuan, X. (1995). China: Lowering maternal mortality in Miyun county, Beijing. World Health Stat Q, 48(1), 11-14. | 1995 | Journal article | ✓ | | | | |
| Binka, F. N., Kubaje, A., Adjuik, M., Williams, L. A., Lengeler, C., Maude, G. H., . . . Smith, P. G. (1996). Impact of permethrin impregnated bednets on child mortality in Kassena-Nankana district, Ghana: a randomized controlled trial. Trop Med Int Health, 1(2), 147-154. doi:http://dx.doi.org/10.1111/j.1365-3156.1996.tb00020.x | 1996 | Journal article | | | ✓ | | |
| Brugha, R. F., & Kevany, J. P. (1996). Maximizing immunization coverage through home visits: a controlled trial in an urban area of Ghana. Bull World Health Organ, 74(5), 517-524. | 1996 | Journal article | | | | ✓ | |
| Conroy, R. M., Elmore-Meegan, M., Joyce, T., McGuigan, K. G., & Barnes, J. (1996). Solar disinfection of drinking water and diarrhoea in Maasai children: a controlled field trial. Lancet, 348(9043), 1695-1697. doi:10.1016/S0140-6736(96)02309-4 | 1996 | Journal article | | | | ✓ | |
| Davies-Adetugbo, A. A. (1996). Promotion of breast feeding in the community: impact of health education programme in rural communities in Nigeria. J Diarrhoeal Dis Res, 14(1), 5-11. | 1996 | Journal article | | | | ✓ | |
| Delacollette, C., Van der Stuyft, P., & Molima, K. (1996). Using community health workers for malaria control: experience in Zaire. Bull World Health Organ, 74(4), 423-430. | 1996 | Journal article | | | | ✓ | |
| Dibley, M. J., Sadjimin, T., Kjolhede, C. L., & Moulton, L. H. (1996). Vitamin A supplementation fails to reduce incidence of acute respiratory illness and diarrhea in preschool-age Indonesian children. J Nutr, 126(2), 434-442. | 1996 | Journal article | | | | ✓ | |
| Fox-Rushby, J. A., & Foord, F. (1996). Costs, effects and cost-effectiveness analysis of a mobile maternal | 1996 | Journal article | ✓ | | | | |

| Chronological reference list: Assessments included in the CBPHC review | | | | | | | |
|---|---|---|---|---|---|---|---|
| | | | Type of assessment carried out | | | | |
| | | | | Child health | | | |
| Reference | Year | Reference type | Maternal health | Neonatal health | Health of children 1-59m | Health of 0-59-m-old children | Equity effect |
| health care service in West Kiang, The Gambia. Health Policy, 35(2), 123-143. doi:http://dx.doi.org/10.1016/0168-8510(95)00774-1 | | | | | | | |
| Ghebreyesus, T. A., Alemayehu, T., Bosman, A., Witten, K. H., & Teklehaimanot, A. (1996). Community participation in malaria control in Tigray region Ethiopia. Acta Trop, 61(2), 145-156. doi:http://dx.doi.org/10.1016/0001-706x(95)00107-p | 1996 | Journal article | | | | ✓ | |
| Lye, M. S., Nair, R. C., Choo, K. E., Kaur, H., & Lai, K. P. (1996). Acute respiratory tract infection: a community-based intervention study in Malaysia. J Trop Pediatr, 42(3), 138-143. doi:http://dx.doi.org/10.1093/tropej/42.3.138 | 1996 | Journal article | | | ✓ | | |
| Magnani, R. J., Rice, J. C., Mock, N. B., Abdoh, A. A., Mercer, D. M., & Tankari, K. (1996). The impact of primary health care services on under-five mortality in rural Niger. Int J Epidemiol, 25(3), 568-577. doi:http://dx.doi.org/10.1093/ije/25.3.568 | 1996 | Journal article | | | | ✓ | |
| Nevill, C. G., Some, E. S., Mung'ala, V. O., Mutemi, W., New, L., Marsh, K., . . . Snow, R. W. (1996). Insecticide-treated bednets reduce mortality and severe morbidity from malaria among children on the Kenyan coast. Trop Med Int Health, 1(2), 139-146. doi:http://dx.doi.org/10.1111/j.1365-3156.1996.tb00019.x | 1996 | Journal article | | | ✓ | | |
| Shahid, N. S., Greenough, W. B., 3rd, Samadi, A. R., Huq, M. I., & Rahman, N. (1996). Hand washing with soap reduces diarrhoea and spread of bacterial pathogens in a Bangladesh village. J Diarrhoeal Dis Res, 14(2), 85-89. | 1996 | Journal article | | | ✓ | | |
| Shiff, C., Checkley, W., Winch, P., Premji, Z., Minjas, J., & Lubega, P. (1996). Changes in weight gain and anaemia attributable to malaria in Tanzanian children living under holoendemic conditions. Trans R Soc Trop Med Hyg, 90(3), 262-265. doi:http://dx.doi.org/10.1016/s0035-9203(96)90240-0 | 1996 | Journal article | | | ✓ | | |
| Sutter, E., & Maphorogo, S. (1996). Integration of community-based trachoma control in primary health care in South Africa. Rev Int Trach Pathol Ocul Trop Subtrop Sante Publique, 73, 19-50. | 1996 | Journal article | | | | ✓ | |
| Berggren, G. (1997). Nutritional education and rehabilitation program: A Save the Children project. In O. Wollinka et al (Eds.), Vietnam Hearth Nutrition Model: Applications in Haiti, Vietnam, and Bangladesh (pp. 43-46). Arlington, VA: World Relief | 1997 | Book section | | | | ✓ | |
| Bilous, J., Maher, C., Tangermann, R. H., Aylward, R. B., Schnur, A., Sanders, R., . . . Omi, S. (1997). The experience of countries in the Western Pacific Region in conducting national immunization days for poliomyelitis eradication. J Infect Dis, 175 Suppl 1, S194-197. doi:http://dx.doi.org/10.1093/infdis/175.supplement_1.s194 | 1997 | Journal article | | | | ✓ | |
| Bosu, W. K., Ahelegbe, D., Edum-Fotwe, E., Bainson, K. A., & Turkson, P. K. (1997). Factors influencing | 1997 | Journal article | | | | ✓ | |

| Chronological reference list: Assessments included in the CBPHC review | | | | | | | |
|---|---|---|---|---|---|---|---|
| | | | Type of assessment carried out | | | | |
| | | | | Child health | | | |
| Reference | Year | Reference type | Mater-nal health | Neo-natal health | Health of children 1-59m | Health of 0-59-m-old children | Equity effect |
| attendance to immunization sessions for children in a rural district of Ghana. Acta Trop, 68(3), 259-267. doi:http://dx.doi.org/10.1016/s0001-706x(97)00094-6 | | | | | | | |
| Burkhalter, B. R., & Northrup, R. S. (1997). Hearth Program at the Hôpital Albert Schweitzer in Haiti Hearth Nutrition Model: Applications in Haiti, Vietnam, and Bangladesh. USAID: Basic Support for Institutionalizing Child Survival (BASICS) Project. Arlington, VA. | 1997 | Book section | | | | ✓ | |
| Chowdhury, A. M., Karim, F., Sarkar, S. K., Cash, R. A., & Bhuiya, A. (1997). The status of ORT (oral rehydration therapy) in Bangladesh: how widely is it used? Health Policy Plan, 12(1), 58-66. doi:http://dx.doi.org/10.1093/heapol/12.1.58 | 1997 | Journal article | | | | ✓ | |
| Dutt, D., & Srinivasa, D. K. (1997). Impact of maternal and child health strategy on child survival in a rural community of Pondicherry. Indian Pediatr, 34(9), 785-792. | 1997 | Journal article | ✓ | | | ✓ | |
| Filoramo, L. (1997). Initiation of the Shishu Kabar Program in Southwestern Bangladesh Hearth Nutrition Model: Applications in Haiti, Vietnam, and Bangladesh. USAID: Basic Support for Institutionalizing Child Survival (BASICS) Project. Arlington, VA. | 1997 | Book section Chapter 6 | | | | ✓ | |
| Habluetzel, A., Diallo, D. A., Esposito, F., Lamizana, L., Pagnoni, F., Lengeler, C., . . . Cousens, S. N. (1997). Do insecticide-treated curtains reduce all-cause child mortality in Burkina Faso? Trop Med Int Health, 2(9), 855-862. doi:http://dx.doi.org/10.1046/j.1365-3156.1997.d01-413.x | 1997 | Journal article | | | ✓ | | |
| Kandeh, H. B., Leigh, B., Kanu, M. S., Kuteh, M., Bangura, J., & Seisay, A. L. (1997). Community motivators promote use of emergency obstetric services in rural Sierra Leone. The Freetown/Makeni PMM Team. Int J Gynaecol Obstet, 59 Suppl 2, S209-218. doi:http://dx.doi.org/10.1016/s0020-7292(97)00167-7 | 1997 | Journal article | ✓ | | | | |
| Knippenberg, R., Alihonou, E., Soucat, A., Oyegbite, K., Calivis, M., Hopwood, I., . . . Ofosu-Amaah, S. (1997). Implementation of the Bamako Initiative: strategies in Benin and Guinea. Int J Health Plann Manage, 12 Suppl 1, S29-47. doi:10.1002/(SICI)1099-1751(199706)12:1+<S29::AID-HPM465>3.0.CO;2-U | 1997 | Journal article | | | | ✓ | |
| Kroeger, A., Meyer, R., Mancheno, M., Gonzalez, M., & Pesse, K. (1997). Operational aspects of bednet impregnation for community-based malaria control in Nicaragua, Ecuador, Peru and Colombia. Trop Med Int Health, 2(6), 589-602. doi:http://dx.doi.org/10.1046/j.1365-3156.1997.d01-319.x | 1997 | Journal article | | | ✓ | | |
| Levy-Bruhl, D., Soucat, A., Osseni, R., Ndiaye, J. M., Dieng, B., De Bethune, X., . . . Knippenberg, R. (1997). The Bamako Initiative in Benin and Guinea: improving the effectiveness of primary health care. Int J Health Plann Manage, 12 Suppl 1, S49-79. doi:10.1002/(SICI)1099-1751(199706)12:1+<S49::AID-HPM466>3.0.CO;2-P | 1997 | Journal article | ✓ | | | ✓ | |

## Chronological reference list: Assessments included in the CBPHC review

| Reference | Year | Reference type | Type of assessment carried out: Mater-nal health | Child health: Neo-natal health | Child health: Health of children 1-59m | Child health: Health of 0-59-m-old children | Equity effect |
|---|---|---|---|---|---|---|---|
| Magbity, E. B., Marbiah, N. T., Maude, G., Curtis, C. F., Bradley, D. J., Greenwood, B. M., . . . Lines, J. D. (1997). Effects of community-wide use of lambdacyhalothrin-impregnated bednets on malaria vectors in rural Sierra Leone. Med Vet Entomol, 11(1), 79-86. doi:http://dx.doi.org/10.1111/j.1365-2915.1997.tb00293.x | 1997 | Journal article | | | ✓ | | |
| Opoku, S. A., Kyei-Faried, S., Twum, S., Djan, J. O., Browne, E. N., & Bonney, J. (1997). Community education to improve utilization of emergency obstetric services in Ghana. The Kumasi PMM Team. Int J Gynaecol Obstet, 59 Suppl 2, S201-207. doi:http://dx.doi.org/10.1016/s0020-7292(97)00166-5 | 1997 | Journal article | ✓ | | | | |
| Pagnoni, F., Convelbo, N., Tiendrebeogo, J., Cousens, S., & Esposito, F. (1997). A community-based programme to provide prompt and adequate treatment of presumptive malaria in children. Trans R Soc Trop Med Hyg, 91(5), 512-517. doi:http://dx.doi.org/10.1016/s0035-9203(97)90006-7 | 1997 | Journal article | | | ✓ | | |
| Rowland, M., Hewitt, S., Durrani, N., Saleh, P., Bouma, M., & Sondorp, E. (1997). Sustainability of pyrethroid-impregnated bednets for malaria control in Afghan communities. Bull World Health Organ, 75(1), 23-29. doi:http://dx.doi.org/10.2458/azu_acku_pamphlet_ra644_m2_r653_1997 | 1997 | Journal article | | | ✓ | | |
| Soucat, A., Levy-Bruhl, D., De Bethune, X., Gbedonou, P., Lamarque, J. P., Bangoura, O., . . . Knippenberg, R. (1997). Affordability, cost-effectiveness and efficiency of primary health care: the Bamako Initiative experience in Benin and Guinea. Int J Health Plann Manage, 12 Suppl 1, S81-108. doi:10.1002/(SICI)1099-1751(199706)12:1+<S81::AID-HPM467>3.0.CO;2-5 | 1997 | Journal article | | | | ✓ | |
| Diallo, I., Ndiaye, B., Pouye, A., Gaye, I. A., Sy, A., Sarr, R., & Tall-Dia, A. (1998). [Community nutrition strategy project: an innovation in community health]. Dakar Med, 43(2), 147-151. | 1998 | Journal article | | | | ✓ | |
| Fraser-Hurt, N., & Lyimo, E. O. (1998). Insecticide-treated nets and treatment service: a trial using public and private sector channels in rural United Republic of Tanzania. Bull World Health Organ, 76(6), 607-615. | 1998 | Journal article | | | ✓ | | |
| Lengeler, C., Armstrong-Schellenberg, J., D'Alessandro, U., Binka, F., & Cattani, J. (1998). Relative versus absolute risk of dying reduction after using insecticide-treated nets for malaria control in Africa. Trop Med Int Health, 3(4), 286-290. doi:http://dx.doi.org/10.1046/j.1365-3156.1998.00236.x | 1998 | Journal article | | | ✓ | | |
| MkNelly, B., & Dunford, C. (1998). Impact of credit with education on mother and their young children's nutrition: Lower Pra rural bank credit with education program in Ghana. Freedom from Hunger Research Paper no. 4 | 1998 | Report | | | | ✓ | ✓ |
| O'Rourke, K., Howard-Grabman, L., & Seoane, G. (1998). Impact of community organization of women on perinatal outcomes in rural Bolivia. Rev Panam | 1998 | Journal article | ✓ | | | ✓ | |

| Chronological reference list: Assessments included in the CBPHC review | | | | | | | |
|---|---|---|---|---|---|---|---|
| | | | Type of assessment carried out | | | | |
| | | | | Child health | | | |
| Reference | Year | Reference type | Mater-nal health | Neo-natal health | Health of children 1-59m | Health of 0-59-m-old children | Equity effect |
| Salud Publica, 3(1), 9-14. doi:http://dx.doi.org/10.1590/s1020-49891998000100002 | | | | | | | |
| Perry, H., Robison, N., Chavez, D., Taja, O., Hilari, C., Shanklin, D., & Wyon, J. (1998). The census-based, impact-oriented approach: its effectiveness in promoting child health in Bolivia. Health Policy Plan, 13(2), 140-151. doi:http://dx.doi.org/10.1093/heapol/13.2.140 | 1998 | Journal article | | | | ✓ | |
| Alvarado, R., Zepeda, A., Rivero, S., Rico, N., Lopez, S., & Diaz, S. (1999). Integrated maternal and infant health care in the postpartum period in a poor neighborhood in Santiago, Chile. Stud Fam Plann, 30(2), 133-141. doi:http://dx.doi.org/10.1111/j.1728-4465.1999.00133.x | 1999 | Journal article | ✓ | | | | |
| Bang, A. T., Bang, R. A., Baitule, S. B., Reddy, M. H., & Deshmukh, M. D. (1999). Effect of home-based neonatal care and management of sepsis on neonatal mortality: field trial in rural India. Lancet, 354(9194), 1955-1961. doi:10.1016/S0140-6736(99)03046-9 | 1999 | Journal article | | ✓ | | | ✓ |
| Care/Kenya. (1999). Community initiatives for child survival Siaya: Final evaluation. | 1999 | Report | | | ✓ | | |
| Conroy, R. M., Meegan, M. E., Joyce, T., McGuigan, K., & Barnes, J. (1999). Solar disinfection of water reduces diarrhoeal disease: an update. Arch Dis Child, 81(4), 337-338. doi:http://dx.doi.org/10.1136/adc.81.4.337 | 1999 | Journal article | | | ✓ | | |
| Fraser-Hurt, N., Felger, I., Edoh, D., Steiger, S., Mashaka, M., Masanja, H., . . . Beck, H. P. (1999). Effect of insecticide-treated bed nets on haemoglobin values, prevalence and multiplicity of infection with Plasmodium falciparum in a randomized controlled trial in Tanzania. Trans R Soc Trop Med Hyg, 93 Suppl 1, 47-51. doi:http://dx.doi.org/10.1016/s0035-9203(99)90327-9 | 1999 | Journal article | | | ✓ | | |
| Kachur, S. P., Phillips-Howard, P. A., Odhacha, A. M., Ruebush, T. K., Oloo, A. J., & Nahlen, B. L. (1999). Maintenance and sustained use of insecticide-treated bednets and curtains three years after a controlled trial in western Kenya. Trop Med Int Health, 4(11), 728-735. doi:http://dx.doi.org/10.1046/j.1365-3156.1999.00481.x | 1999 | Journal article | | | ✓ | | |
| Kapil, U., & Pradhan, R. (1999). Integrated Child Development Services scheme (ICDS) and its impact on nutritional status of children in India and recent initiatives. Indian J Public Health, 43(1), 21-25. | 1999 | Journal article | | | | ✓ | |
| Lartey, A., Manu, A., Brown, K. H., Peerson, J. M., & Dewey, K. G. (1999). A randomized, community-based trial of the effects of improved, centrally processed complementary foods on growth and micronutrient status of Ghanaian infants from 6 to 12 mo of age. Am J Clin Nutr, 70(3), 391-404. | 1999 | Journal article | | | | ✓ | |
| Marek, T., Diallo, I., Ndiaye, B., & Rakotosalama, J. (1999). Successful contracting of prevention services: fighting malnutrition in Senegal and Madagascar. | 1999 | Journal article | | | | ✓ | |

| Chronological reference list: Assessments included in the CBPHC review | | | | | | | |
|---|---|---|---|---|---|---|---|
| | | | Type of assessment carried out | | | | |
| | | | | Child health | | | |
| Reference | Year | Reference type | Mater-nal health | Neo-natal health | Health of children 1-59m | Health of 0-59-m-old children | Equity effect |
| Health Policy Plan, 14(4), 382-389. doi:http://dx.doi.org/10.1093/heapol/14.4.382 | | | | | | | |
| Marsh, V. M., Mutemi, W. M., Muturi, J., Haaland, A., Watkins, W. M., Otieno, G., & Marsh, K. (1999). Changing home treatment of childhood fevers by training shop keepers in rural Kenya. Trop Med Int Health, 4(5), 383-389. doi:http://dx.doi.org/10.1046/j.1365-3156.1999.00403.x | 1999 | Journal article | | | | ✓ | |
| Neumann, N. A., Victora, C. G., Halpern, R., Guimaraes, P. R., & Cesar, J. A. (1999). [Assessment of the performance of Pastoral de Crianca, a health support group, in promoting child survival and health education in Criciuma,a city in southern Brazil]. Rev Panam Salud Publica, 5(6), 400-410. doi:http://dx.doi.org/10.1590/s1020-49891999000500004 | 1999 | Journal article | | ✓ | | | |
| O'Connor, J., Lynch, M., Vitale, S., & West, S. (1999). Characteristics of effective village treatment assistants: the Kongwa Trachoma Project. Ophthalmic Epidemiol, 6(4), 257-265. doi:http://dx.doi.org/10.1076/opep.6.4.257.4186 | 1999 | Journal article | | | | ✓ | |
| Penny, M. E., Peerson, J. M., Marin, R. M., Duran, A., Lanata, C. F., Lonnerdal, B., . . . Brown, K. H. (1999). Randomized, community-based trial of the effect of zinc supplementation, with and without other micronutrients, on the duration of persistent childhood diarrhea in Lima, Peru. J Pediatr, 135(2 Pt 1), 208-217. doi:http://dx.doi.org/10.1016/s0022-3476(99)70024-7 | 1999 | Journal article | | | ✓ | | |
| Perez-Cuevas, R., Reyes, H., Pego, U., Tome, P., Ceja, K., Flores, S., & Gutierrez, G. (1999). Immunization promotion activities: are they effective in encouraging mothers to immunize their children? Soc Sci Med, 49(7), 921-932. doi:http://dx.doi.org/10.1016/s0277-9536(99)00178-1 | 1999 | Journal article | | | | ✓ | |
| Quick, R. E., Venczel, L. V., Mintz, E. D., Soleto, L., Aparicio, J., Gironaz, M., . . . Tauxe, R. V. (1999). Diarrhoea prevention in Bolivia through point-of-use water treatment and safe storage: a promising new strategy. Epidemiol Infect, 122(1), 83-90. doi:http://dx.doi.org/10.1017/s0950268898001782 | 1999 | Journal article | | | ✓ | | |
| Sternin, M., Sternin, J., & Marsh, D. L. (1999). Scaling up a poverty alleviation and nutrition program in Vietnam. In T. Marchione (Ed.), Scaling up, scaling down: Overcoming malnutrition in developing countries (pp. 97-117). Amsterdam, The Netherlands: Gordon and Breach. | 1999 | Book Section | | | | ✓ | |
| Anand, K., Kant, S., Kumar, G., & Kapoor, S. K. (2000). "Development" is not essential to reduce infant mortality rate in India: experience from the Ballabgarh project. J Epidemiol Community Health, 54(4), 247-253. doi:http://dx.doi.org/10.1136/jech.54.4.247 | 2000 | Journal article | | ✓ | | | |
| Awasthi, S., Pande, V. K., & Fletcher, R. H. (2000). Effectiveness and cost-effectiveness of albendazole in | 2000 | Journal article | | | | ✓ | |

| Chronological reference list: Assessments included in the CBPHC review | | | | | | | |
|---|---|---|---|---|---|---|---|
| | | | Type of assessment carried out | | | | |
| | | | | Child health | | | |
| **Reference** | **Year** | **Reference type** | **Mater-nal health** | **Neo-natal health** | **Health of children 1-59m** | **Health of 0-59-m-old children** | **Equity effect** |
| improving nutritional status of pre-school children in urban slums. Indian Pediatr, 37(1), 19-29. | | | | | | | |
| Fiedler, J. L. (2000). The Nepal National Vitamin A Program: prototype to emulate or donor enclave? Health Policy Plan, 15(2), 145-156. doi:http://dx.doi.org/10.1093/heapol/15.2.145 | 2000 | Journal article | | | | ✓ | |
| Haider, R., Ashworth, A., Kabir, I., & Huttly, S. R. (2000). Effect of community-based peer counsellors on exclusive breastfeeding practices in Dhaka, Bangladesh: a randomised controlled trial [see commments]. Lancet, 356(9242), 1643-1647. doi:http://dx.doi.org/10.1016/s0140-6736(00)03159-7 | 2000 | Journal article | | ✓ | | | |
| Kidane, G., & Morrow, R. H. (2000). Teaching mothers to provide home treatment of malaria in Tigray, Ethiopia: a randomised trial. Lancet, 356(9229), 550-555. doi:10.1016/S0140-6736(00)02580-0 | 2000 | Journal article | | | ✓ | | |
| Malekafzali, H., Abdollahi, Z., Mafi, A., & Naghavi, M. (2000). Community-based nutritional intervention for reducing malnutrition among children under 5 years of age in the Islamic Republic of Iran. East Mediterr Health J, 6(2-3), 238-245. | 2000 | Journal article | | ✓ | | | |
| Wafula, E. M., Kinyanjui, M. M., Nyabola, L., & Tenambergen, E. D. (2000). Effect of improved stoves on prevalence of acute respiration infection and conjunctivitis among children and women in a rural community in Kenya. East Afr Med J, 77(1), 37-41. doi:http://dx.doi.org/10.4314/eamj.v77i1.46379 | 2000 | Journal article | | | | ✓ | ✓ |
| Abdulla, S., Schellenberg, J. A., Nathan, R., Mukasa, O., Marchant, T., Smith, T., . . . Lengeler, C. (2001). Impact on malaria morbidity of a programme supplying insecticide treated nets in children aged under 2 years in Tanzania: community cross sectional study. BMJ, 322(7281), 270-273. doi:http://dx.doi.org/10.1136/bmj.322.7281.270 | 2001 | Journal article | | | | ✓ | |
| Ali, M., Emch, M., Tofail, F., & Baqui, A. H. (2001). Implications of health care provision on acute lower respiratory infection mortality in Bangladeshi children. Soc Sci Med, 52(2), 267-277. doi:http://dx.doi.org/10.1016/s0277-9536(00)00120-9 | 2001 | Journal article | | | ✓ | | |
| Babalola, S., Sakolsky, N., Vondrasek, C., Mounlom, D., Brown, J., & Tchupo, J. P. (2001). The impact of a community mobilization project on health-related knowledge and practices in Cameroon. J Community Health, 26(6), 459-477. | 2001 | Journal article | ✓ | | | ✓ | |
| Bhandari, N., Bahl, R., Nayyar, B., Khokhar, P., Rohde, J. E., & Bhan, M. K. (2001). Food supplementation with encouragement to feed it to infants from 4 to 12 months of age has a small impact on weight gain. J Nutr, 131(7), 1946-1951. | 2001 | Journal article | | | | ✓ | |
| Food for the Hungry/Mozambique (2001). Food for the Hungry International/Mozambique Health/Nutrition Program: Final evaluation report. | 2001 | Report | | | | ✓ | |
| Gloyd, S., Floriano, F., Seunda, M., Chadreque, M. A., Nyangezi, J. M., & Platas, A. (2001). Impact of traditional birth attendant training in Mozambique: a | 2001 | Journal article | ✓ | | | | |

| Chronological reference list: Assessments included in the CBPHC review | | | | | | | | |
|---|---|---|---|---|---|---|---|---|
| | | | Type of assessment carried out | | | | | |
| | | | | Child health | | | | |
| Reference | Year | Reference type | Mater-nal health | Neo-natal health | Health of children 1-59m | Health of 0-59-m-old children | Equity effect |
| controlled study. J Midwifery Womens Health, 46(4), 210-216. doi:http://dx.doi.org/10.1016/s1526-9523(01)00142-8 | | | | | | | |
| Osendarp, S. J., van Raaij, J. M., Darmstadt, G. L., Baqui, A. H., Hautvast, J. G., & Fuchs, G. J. (2001). Zinc supplementation during pregnancy and effects on growth and morbidity in low birthweight infants: a randomised placebo controlled trial. Lancet, 357(9262), 1080-1085. doi:http://dx.doi.org/10.1016/s0140-6736(00)04260-4 | 2001 | Journal article | | ✓ | | | |
| Rahman, M. M., Vermund, S. H., Wahed, M. A., Fuchs, G. J., Baqui, A. H., & Alvarez, J. O. (2001). Simultaneous zinc and vitamin A supplementation in Bangladeshi children: randomised double blind controlled trial. BMJ, 323(7308), 314-318. doi:http://dx.doi.org/10.1136/bmj.323.7308.314 | 2001 | Journal article | | | | ✓ | |
| Schellenberg, J. R., Abdulla, S., Nathan, R., Mukasa, O., Marchant, T. J., Kikumbih, N., . . . Lengeler, C. (2001). Effect of large-scale social marketing of insecticide-treated nets on child survival in rural Tanzania. Lancet, 357(9264), 1241-1247. doi:10.1016/S0140-6736(00)04404-4 | 2001 | Journal article | | | ✓ | | |
| Baqui, A. H., Black, R. E., El Arifeen, S., Yunus, M., Chakraborty, J., Ahmed, S., & Vaughan, J. P. (2002). Effect of zinc supplementation started during diarrhoea on morbidity and mortality in Bangladeshi children: community randomised trial. BMJ, 325(7372), 1059. doi:http://dx.doi.org/10.1136/bmj.325.7372.1059 | 2002 | Journal article | | | ✓ | | |
| Bhandari, N., Bahl, R., Taneja, S., Strand, T., Molbak, K., Ulvik, R. J., . . . Bhan, M. K. (2002). Effect of routine zinc supplementation on pneumonia in children aged 6 months to 3 years: randomised controlled trial in an urban slum. BMJ, 324(7350), 1358. doi:http://dx.doi.org/10.1136/bmj.324.7350.1358 | 2002 | Journal article | | | ✓ | | |
| Bhandari, N., Bahl, R., Taneja, S., Strand, T., Molbak, K., Ulvik, R. J., . . . Bhan, M. K. (2002). Substantial reduction in severe diarrheal morbidity by daily zinc supplementation in young north Indian children. Pediatrics, 109(6), e86. doi:http://dx.doi.org/10.1007/bf02725586 | 2002 | Journal article | | | ✓ | | |
| Bhuiya, A., & Chowdhury, M. (2002). Beneficial effects of a woman-focused development programme on child survival: evidence from rural Bangladesh. Soc Sci Med, 55(9), 1553-1560. doi:http://dx.doi.org/10.1016/s0277-9536(01)00287-8 | 2002 | Journal article | | | | ✓ | ✓ |
| Cesar, J. A., Cavaleti, M. A., Holthausen, R. S., & de Lima, L. G. (2002). [Changes in child health indicators in a municipality with community health workers: the case of Itapirapua Paulista, Vale do Ribeira, Sao Paulo State, Brazil]. Cad Saude Publica, 18(6), 1647-1654. doi:http://dx.doi.org/10.1590/s0102-311x2002000600019 | 2002 | Journal article | | | | ✓ | |
| Collins, S., & Sadler, K. (2002). Outpatient care for severely malnourished children in emergency relief programmes: a retrospective cohort study. Lancet, | 2002 | Journal article | | | | ✓ | |

| Chronological reference list: Assessments included in the CBPHC review | | | | | | | |
|---|---|---|---|---|---|---|---|
| | | | Type of assessment carried out | | | | |
| | | | | Child health | | | |
| Reference | Year | Reference type | Maternal health | Neonatal health | Health of children 1-59m | Health of 0-59-m-old children | Equity effect |
| 360(9348), 1824-1830. doi:10.1016/S0140-6736(02)11770-3 | | | | | | | |
| Debpuur, C., Phillips, J. F., Jackson, E. F., Nazzar, A., Ngom, P., & Binka, F. N. (2002). The impact of the Navrongo Project on contraceptive knowledge and use, reproductive preferences, and fertility. Stud Fam Plann, 33(2), 141-164. doi:http://dx.doi.org/10.1111/j.1728-4465.2002.00141.x | 2002 | Journal article | | | | ✓ | ✓ |
| Emond, A., Pollock, J., Da Costa, N., Maranhao, T., & Macedo, A. (2002). The effectiveness of community-based interventions to improve maternal and infant health in the Northeast of Brazil. Rev Panam Salud Publica, 12(2), 101-110. doi:http://dx.doi.org/10.1590/s1020-49892002000800005 | 2002 | Journal article | ✓ | | | ✓ | |
| Gopaldas, T., & Gujral, S. (2002). Empowering a tea-plantation community to improve its micronutrient health. Food and Nutrition Bulletin, 23(2), 143-152. doi:http://dx.doi.org/10.1177/156482650202300203 | 2002 | Journal article | | | | ✓ | |
| Hadi, A. (2002). Integrating prevention of acute respiratory infections with micro-credit programme: experience of BRAC, Bangladesh. Public Health, 116(4), 238-244. doi:10.1038/sj.ph.1900863 | 2002 | Journal article | | | | ✓ | |
| Haider, R., Kabir, I., Huttly, S. R., & Ashworth, A. (2002). Training peer counselors to promote and support exclusive breastfeeding in Bangladesh. J Hum Lact, 18(1), 7-12. doi:http://dx.doi.org/10.1177/089033440201800102 | 2002 | Journal article | | ✓ | | | |
| Hung le, Q., Vries, P. J., Giao, P. T., Nam, N. V., Binh, T. Q., Chong, M. T., . . . Kager, P. A. (2002). Control of malaria: a successful experience from Viet Nam. Bull World Health Organ, 80(8), 660-666. | 2002 | Journal article | | | ✓ | | |
| Khan, N. C., Khoi, H. H., Giay, T., Nhan, N. T., Nhan, T. T., Dung, N. C., . . . Luy, H. T. (2002). Control of Vitamin A deficiency in Vietnam: Achievements and future orientation. Food and Nutrition Bulletin, 23(2). doi:http://dx.doi.org/10.1177/156482650202300202 | 2002 | Journal article | | | | ✓ | |
| Mackintosh, U. A. T., Marsh, D. R., & Schroder, D. G. (2002). Sustained positive deviant child care practices and their effects on child growth in Viet Nam. Food and Nutrition Bulletin, 23(4), 16-24. doi:http://dx.doi.org/10.1177/15648265020234s204 | 2002 | Journal article | | | | ✓ | |
| Quick, R. E., Kimura, A., Thevos, A., Tembo, M., Shamputa, I., Hutwagner, L., & Mintz, E. (2002). Diarrhea prevention through household-level water disinfection and safe storage in Zambia. Am J Trop Med Hyg, 66(5), 584-589. | 2002 | Journal article | | | ✓ | | |
| Rahman, M. M., Tofail, F., Wahed, M. A., Fuchs, G. J., Baqui, A. H., & Alvarez, J. O. (2002). Short-term supplementation with zinc and vitamin A has no significant effect on the growth of undernourished Bangladeshi children. Am J Clin Nutr, 75(1), 87-91. | 2002 | Journal article | | | | ✓ | |
| Schroeder, D. G., Pachon, H., Dearden, K. A., Ha, T. T., Lang, T. T., & Marsh, D. R. (2002). An integrated child | 2002 | Journal article | | | | ✓ | |

| Chronological reference list: Assessments included in the CBPHC review | | | | | | | |
|---|---|---|---|---|---|---|---|
| | | | Type of assessment carried out | | | | |
| | | | | Child health | | | |
| Reference | Year | Reference type | Mater-nal health | Neo-natal health | Health of children 1-59m | Health of 0-59-m-old children | Equity effect |
| nutrition intervention improved growth of younger more malnourished children in northern Viet Nam. Food and Nutrition Bulletin, vol 23 supplement 50-58 | | | | | | | |
| Sripaipan, T., Schroeder, D. G., Marsh, D. R., Pachon, H., Dearden, K. A., Ha, T. T., & Lang, T. T. (2002). Effect of an integrated nutrition program on child morbidity due to respiratory infection and diarrhea in northen Viet Nam. Food and Nutrition Bulletin, vol 23 supplement 67-74 | 2002 | Journal article | | | ✓ | | |
| Ahluwalia, I. B., Schmid, T., Kouletio, M., & Kanenda, O. (2003). An evaluation of a community-based approach to safe motherhood in northwestern Tanzania. Int J Gynaecol Obstet, 82(2), 231-240. doi:http://dx.doi.org/10.1016/s0020-7292(03)00081-x | 2003 | Journal article | ✓ | | | | |
| Baqui, A. H., Zaman, K., Persson, L. A., El Arifeen, S., Yunus, M., Begum, N., & Black, R. E. (2003). Simultaneous weekly supplementation of iron and zinc is associated with lower morbidity due to diarrhea and acute lower respiratory infection in Bangladeshi infants. J Nutr, 133(12), 4150-4157. | 2003 | Journal article | | | | ✓ | |
| BASICS II/Uganda (2003). Increasing immunization coverage in Uganda: The community problem solving and strategy development approach. | 2003 | Report | | | | ✓ | |
| Bhandari, N., Bahl, R., Mazumdar, S., Martines, J., Black, R. E., Bhan, M. K., & Infant Feeding Study, G. (2003). Effect of community-based promotion of exclusive breastfeeding on diarrhoeal illness and growth: a cluster randomised controlled trial. Lancet, 361(9367), 1418-1423. doi:10.1016/S0140-6736(03)13134-0 | 2003 | Journal article | | | | ✓ | |
| Christian, P., Khatry, S. K., Katz, J., Pradhan, E. K., LeClerq, S. C., Shrestha, S. R., . . . West, K. P., Jr. (2003). Effects of alternative maternal micronutrient supplements on low birth weight in rural Nepal: double blind randomised community trial. BMJ, 326(7389), 571. doi:10.1136/bmj.326.7389.571 | 2003 | Journal article | | ✓ | | | |
| Christian, P., West, K. P., Khatry, S. K., Leclerq, S. C., Pradhan, E. K., Katz, J., . . . Sommer, A. (2003). Effects of maternal micronutrient supplementation on fetal loss and infant mortality: a cluster-randomized trial in Nepal. Am J Clin Nutr, 78(6), 1194-1202. | 2003 | Journal article | | | | ✓ | |
| Gupta, D. N., Mondal, S. K., Ghosh, S., Rajendran, K., Sur, D., & Manna, B. (2003). Impact of zinc supplementation on diarrhoeal morbidity in rural children of West Bengal, India. Acta Paediatr, 92(5), 531-536. doi:http://dx.doi.org/10.1111/j.1651-2227.2003.tb02501.x | 2003 | Journal article | | | | ✓ | |
| Hadi, A. (2003). Management of acute respiratory infections by community health volunteers: experience of Bangladesh Rural Advancement Committee (BRAC). Bull World Health Organ, 81(3), 183-189. | 2003 | Journal article | | | | ✓ | |
| Helen Keller International (2003). Community-based Iron+Folic Acid Supplementation and Nutrition | 2003 | Report | ✓ | | | | |

| Chronological reference list: Assessments included in the CBPHC review | | | | | | | |
|---|---|---|---|---|---|---|---|
| | | | Type of assessment carried out | | | | |
| | | | | Child health | | | |
| Reference | Year | Reference type | Mater-nal health | Neo-natal health | Health of children 1-59m | Health of 0-59-m-old children | Equity effect |
| Education for Pregnant Women: Manica Province. Maputo, Mozambique | | | | | | | |
| Lynch, M., West, S., Munoz, B., Frick, K. D., & Mkocha, H. A. (2003). Azithromycin treatment coverage in Tanzanian children using community volunteers. Ophthalmic Epidemiol, 10(3), 167-175. doi:http://dx.doi.org/10.1076/opep.10.3.167.15082 | 2003 | Journal article | | | | ✓ | |
| Nanan, D., White, F., Azam, I., Afsar, H., & Hozhabri, S. (2003). Evaluation of a water, sanitation, and hygiene education intervention on diarrhoea in northern Pakistan. Bull World Health Organ, 81(3), 160-165. | 2003 | Journal article | | | | ✓ | |
| Pasha, O., Del Rosso, J., Mukaka, M., & Marsh, D. (2003). The effect of providing fansidar (sulfadoxine-pyrimethamine) in schools on mortality in school-age children in Malawi. Lancet, 361(9357), 577-578. doi:http://dx.doi.org/10.1016/s0140-6736(03)12511-1 | 2003 | Journal article | | | ✓ | | |
| Perry, H. B., Shanklin, D. S., & Schroeder, D. G. (2003). Impact of a community-based comprehensive primary healthcare programme on infant and child mortality in Bolivia. J Health Popul Nutr, 21(4), 383-395. | 2003 | Journal article | | | | ✓ | |
| Phillips-Howard, P. A., Nahlen, B. L., Alaii, J. A., ter Kuile, F. O., Gimnig, J. E., Terlouw, D. J., . . . Hawley, W. A. (2003). The efficacy of permethrin-treated bed nets on child mortality and morbidity in western Kenya I. Development of infrastructure and description of study site. Am J Trop Med Hyg, 68(4 Suppl), 3-9. | 2003 | Journal article | | | ✓ | | |
| Pitt, M. M., Khandker, S. R., Chowdhury, O. H., & Millimet, D. L. (2003). Credit programs for te poor and the health status of children in rural Bangladesh. International Economic Review, 44(1), 87-113. doi:http://dx.doi.org/10.1111/1468-2354.t01-1-00063 | 2003 | Journal article | | | | ✓ | |
| Plan/Ecuador. (2003). IMCI strategy and its impact on child mortality. | 2003 | Report | | | | ✓ | |
| Rahmathullah, L., Tielsch, J. M., Thulasiraj, R. D., Katz, J., Coles, C., Devi, S., . . . Kamaraj, C. (2003). Impact of supplementing newborn infants with vitamin A on early infant mortality: community based randomised trial in southern India. BMJ, 327(7409), 254. doi:10.1136/bmj.327.7409.254 | 2003 | Journal article | | | | ✓ | |
| Sirima, S. B., Konate, A., Tiono, A. B., Convelbo, N., Cousens, S., & Pagnoni, F. (2003). Early treatment of childhood fevers with pre-packaged antimalarial drugs in the home reduces severe malaria morbidity in Burkina Faso. Trop Med Int Health, 8(2), 133-139. doi: http://dx.doi.org/10.1046/j.1365-3156.2003.00997.x | 2003 | Journal article | | | | ✓ | |
| Sobsey, M. D., Handzel, T., & Venczel, L. (2003). Chlorination and safe storage of household drinking water in developing countries to reduce waterborne disease. Water Sci Technol, 47(3), 221-228. | 2003 | Journal article | | | ✓ | | |
| Sur, D., Gupta, D. N., Mondal, S. K., Ghosh, S., Manna, B., Rajendran, K., & Bhattacharya, S. K. (2003). Impact of zinc supplementation on diarrheal morbidity and growth pattern of low birth weight infants in kolkata, India: a randomized, double-blind, placebo-controlled, | 2003 | Journal article | | | ✓ | | |

| Chronological reference list: Assessments included in the CBPHC review | | | | | | | |
|---|---|---|---|---|---|---|---|
| | | | Type of assessment carried out | | | | |
| | | | | Child health | | | |
| Reference | Year | Reference type | Mater-nal health | Neo-natal health | Health of children 1-59m | Health of 0-59-m-old children | Equity effect |
| community-based study. Pediatrics, 112(6 Pt 1), 1327-1332. doi:http://dx.doi.org/10.1542/peds.112.6.1327 | | | | | | | |
| Turan, J. M., & Say, L. (2003). Community-based antenatal education in Istanbul, Turkey: effects on health behaviours. Health Policy Plan, 18(4), 391-398. doi:http://dx.doi.org/10.1093/heapol/czg047 | 2003 | Journal article | ✓ | | | ✓ | |
| Winch, P. J., Bagayoko, A., Diawara, A., Kane, M., Thiero, F., Gilroy, K., . . . Swedberg, E. (2003). Increases in correct administration of chloroquine in the home and referral of sick children to health facilities through a community-based intervention in Bougouni District, Mali. Trans R Soc Trop Med Hyg, 97(5), 481-490. doi:http://dx.doi.org/10.1016/s0035-9203(03)80001-9 | 2003 | Journal article | | | | ✓ | |
| Arifeen, S. E., Blum, L. S., Hoque, D. M., Chowdhury, E. K., Khan, R., Black, R. E., . . . Bryce, J. (2004). Integrated Management of Childhood Illness (IMCI) in Bangladesh: early findings from a cluster-randomised study. Lancet, 364(9445), 1595-1602. doi:10.1016/S0140-6736(04)17312-1 | 2004 | Journal article | | | | ✓ | |
| Aubel, J., Toure, I., & Diagne, M. (2004). Senegalese grandmothers promote improved maternal and child nutrition practices: the guardians of tradition are not averse to change. Soc Sci Med, 59(5), 945-959. doi:10.1016/j.socscimed.2003.11.044 | 2004 | Journal article | | | | ✓ | |
| Awoonor-Williams, J. K., Feinglass, E. S., Tobey, R., Vaughan-Smith, M. N., Nyonator, F. K., & Jones, T. C. (2004). Bridging the gap between evidence-based innovation and national health-sector reform in Ghana. Stud Fam Plann, 35(3), 161-177. doi:http://dx.doi.org/10.1111/j.1728-4465.2004.00020.x | 2004 | Journal article | ✓ | ✓ | | | ✓ |
| BASICS II/Madagascar (2004). Improving family health using an integrated community-based approach: Madagarcar Case Study. | 2004 | Report | | | | ✓ | |
| BASICS II/Senegal (2004). CHWs in Senegal can appropriately treat pneumonia with cotrimoxazole. | 2004 | Report | | | | ✓ | |
| Bhandari, N., Mazumder, S., Bahl, R., Martines, J., Black, R. E., Bhan, M. K., & Infant Feeding Study, G. (2004). An educational intervention to promote appropriate complementary feeding practices and physical growth in infants and young children in rural Haryana, India. J Nutr, 134(9), 2342-2348. | 2004 | Journal article | | | | ✓ | |
| Christian, P., Khatry, S. K., & West, K. P., Jr. (2004). Antenatal anthelmintic treatment, birthweight, and infant survival in rural Nepal. Lancet, 364(9438), 981-983. doi:10.1016/S0140-6736(04)17023-2 | 2004 | Journal article | | ✓ | | | |
| Diallo, D. A., Cousens, S. N., Cuzin-Ouattara, N., Nebie, I., Ilboudo-Sanogo, E., & Esposito, F. (2004). Child mortality in a West African population protected with insecticide-treated curtains for a period of up to 6 years. Bull World Health Organ, 82(2), 85-91. | 2004 | Journal article | | | ✓ | | |
| Dohn, A. L., Chavez, A., Dohn, M. N., Saturria, L., & Pimentel, C. (2004). Changes in health indicators related to health promotion and microcredit programs | 2004 | Journal article | | | | ✓ | |

| Chronological reference list: Assessments included in the CBPHC review | | | | | | | |
|---|---|---|---|---|---|---|---|
| | | | Type of assessment carried out | | | | |
| | | | | Child health | | | |
| Reference | Year | Reference type | Mater-nal health | Neo-natal health | Health of children 1-59m | Health of 0-59-m-old children | Equity effect |
| in the Dominican Republic. Rev Panam Salud Publica, 15(3), 185-193. doi:http://dx.doi.org/10.1590/s1020-49892004000300007 | | | | | | | |
| Greer, G., Akinpelumi, A., Madueke, L., Plowman, B., Fapohunda, B., Tawfik, Y., . . . Lennox, B. (2004). Improving management of chilhood malaria in Nigeria and Uganda by improving practices of patent medicine vendors. BASICS II/USAID | 2004 | Report | | | ✓ | | |
| Guyon, A., Rambeloson, Z., Hainsworth, M., & Quinn, V. (2004). Assesing a behavior change strategy for The Essential Nutrition Actions, Immunization and Family Planning: Antananarivo and Fianarantsoa Provinces, Madagascar. | 2004 | Report | | | | ✓ | |
| Lindblade, K. A., Eisele, T. P., Gimnig, J. E., Alaii, J. A., Odhiambo, F., ter Kuile, F. O., . . . Slutsker, L. (2004). Sustainability of reductions in malaria transmission and infant mortality in western Kenya with use of insecticide-treated bednets: 4 to 6 years of follow-up. JAMA, 291(21), 2571-2580. doi:10.1001/jama.291.21.2571 | 2004 | Journal article | | | | ✓ | |
| Luby, S. P., Agboatwalla, M., Painter, J., Altaf, A., Billhimer, W. L., & Hoekstra, R. M. (2004). Effect of intensive handwashing promotion on childhood diarrhea in high-risk communities in Pakistan: a randomized controlled trial. JAMA, 291(21), 2547-2554. doi:10.1001/jama.291.21.2547 | 2004 | Journal article | | | ✓ | | |
| Manandhar, D. S., Osrin, D., Shrestha, B. P., Mesko, N., Morrison, J., Tumbahangphe, K. M., . . . Members of the, M. M. t. t. (2004). Effect of a participatory intervention with women's groups on birth outcomes in Nepal: cluster-randomised controlled trial. Lancet, 364(9438), 970-979. doi:10.1016/S0140-6736(04)17021-9 | 2004 | Journal article | ✓ | | | ✓ | |
| Mercer, A., Khan, M. H., Daulatuzzaman, M., & Reid, J. (2004). Effectiveness of an NGO primary health care programme in rural Bangladesh: evidence from the management information system. Health Policy Plan, 19(4), 187-198. doi:http://dx.doi.org/10.1093/heapol/czh024 | 2004 | Journal article | ✓ | | | ✓ | ✓ |
| Minnesota International Health Volunteers/Uganda (2004). Improving malaria case management in Uganda communities: Lessons from the field. | 2004 | Report | | | ✓ | | |
| Minnesota International Health Volunteers/Uganda (2004). Uganda family planning programs: Lessons from the field. | 2004 | Report | | | | ✓ | |
| Morris, S. S., Flores, R., Olinto, P., & Medina, J. M. (2004). Monetary incentives in primary health care and effects on use and coverage of preventive health care interventions in rural Honduras: cluster randomised trial. Lancet, 364(9450), 2030-2037. doi:10.1016/S0140-6736(04)17515-6 | 2004 | Journal article | ✓ | | | ✓ | |
| Nyonator, F., Jones, T. C., Miller, R. A., Phillips, J. F., & Awoonor-Williams, J. K. (2004-2005). Guiding the Ghana community-based health planning and services approach to scaling up with qualitative systems | 2004 | Journal article | | | | ✓ | ✓ |

| Chronological reference list: Assessments included in the CBPHC review | | | | | | | |
|---|---|---|---|---|---|---|---|
| | | | Type of assessment carried out | | | | |
| | | | | Child health | | | |
| Reference | Year | Reference type | Mater-nal health | Neo-natal health | Health of children 1-59m | Health of 0-59-m-old children | Equity effect |
| appraisal. Int Quaterly of Community Health Education, 23(3), 189-213. doi:http://dx.doi.org/10.2190/ngm3-fydt-5827-ml1p | | | | | | | |
| Penny, M. E., Marin, R. M., Duran, A., Peerson, J. M., Lanata, C. F., Lonnerdal, B., . . . Brown, K. H. (2004). Randomized controlled trial of the effect of daily supplementation with zinc or multiple micronutrients on the morbidity, growth, and micronutrient status of young Peruvian children. Am J Clin Nutr, 79(3), 457-465. | 2004 | Journal article | | | | ✓ | |
| Perez, F., Mukotekwa, T., Miller, A., Orne-Gliemann, J., Glenshaw, M., Chitsike, I., & Dabis, F. (2004). Implementing a rural programme of prevention of mother-to-child transmission of HIV in Zimbabwe: first 18 months of experience. Trop Med Int Health, 9(7), 774-783. doi:10.1111/j.1365-3156.2004.01264.x | 2004 | Journal article | ✓ | | | | |
| Plan/Cameroon. (2004). Child survival project: Final evaluation report. | 2004 | Report | | | | ✓ | |
| World Relief/Malawi (2004). Final evaluation of the Tiweko Tose child survival project. | 2004 | Report | | | | ✓ | |
| Save the Children/Australia (2004). Evaluation of Sayaboury primary health care project, Phase IV: Lao People's Democratic Republic. | 2004 | Report | | | | ✓ | |
| Sibley, L., Buffington, S. T., & Haileyesus, D. (2004). The American College of Nurse-Midwives' home-based lifesaving skills program: a review of the Ethiopia field test. J Midwifery Womens Health, 49(4), 320-328. doi:10.1016/j.jmwh.2004.03.013 | 2004 | Journal article | ✓ | | | | |
| Adventist Development Relief Agency/Guinea. (2005). Child survival XVI: Final Evaluation Siguiri Prefecture. | 2005 | Report | ✓ | | | ✓ | |
| Ali, M., Asefaw, T., Byass, P., Beyene, H., & Pedersen, F. K. (2005). Helping northern Ethiopian communities reduce childhood mortality: population-based intervention trial. Bull World Health Organ, 83(1), 27-33. doi:/S0042-96862005000100011 | 2005 | Journal article | | ✓ | | | |
| Bang, A. T., Reddy, H. M., Deshmukh, M. D., Baitule, S. B., & Bang, R. A. (2005). Neonatal and infant mortality in the ten years (1993 to 2003) of the Gadchiroli field trial: effect of home-based neonatal care. J Perinatol, 25 Suppl 1, S92-107. doi:10.1038/sj.jp.7211277 | 2005 | Journal article | | ✓ | | | ✓ |
| Bhandari, N., Mazumder, S., Bahl, R., Martines, J., Black, R. E., Bhan, M. K., & Infant Feeding Study, G. (2005). Use of multiple opportunities for improving feeding practices in under-twos within child health programmes. Health Policy Plan, 20(5), 328-336. doi:10.1093/heapol/czi039 | 2005 | Journal article | | | | ✓ | |
| Bishai, D., Kumar, K. C. S., Waters, H., Koenig, M., Katz, J., Khatry, S. K., & West, K. P., Jr. (2005). The impact of vitamin A supplementation on mortality inequalities among children in Nepal. Health Policy Plan, 20(1), 60-66. doi:10.1093/heapol/czi007 | 2005 | Journal article | | | | ✓ | ✓ |
| Brooks, W. A., Santosham, M., Naheed, A., Goswami, D., Wahed, M. A., Diener-West, M., . . . Black, R. E. (2005). Effect of weekly zinc supplements on incidence of pneumonia and diarrhoea in children | 2005 | Journal article | | | ✓ | | |

| Chronological reference list: Assessments included in the CBPHC review | | | | | | | |
|---|---|---|---|---|---|---|---|
| | | | Type of assessment carried out | | | | |
| | | | | Child health | | | |
| Reference | Year | Reference type | Mater-nal health | Neo-natal health | Health of children 1-59m | Health of 0-59-m-old children | Equity effect |
| younger than 2 years in an urban, low-income population in Bangladesh: randomised controlled trial. Lancet, 366(9490), 999-1004. doi:10.1016/S0140-6736(05)67109-7 | | | | | | | |
| Cesar, J. A., Goncalves, T. S., Neumann, N. A., Oliveira Filho, J. A., & Diziekaniak, A. C. (2005). [Child health in poor areas of North and Northeast Brazil: a comparison of areas covered by the Children's Mission and control areas]. Cad Saude Publica, 21(6), 1845-1855. doi:/S0102-311X2005000600034 | 2005 | Journal article | | | | ✓ | ✓ |
| Ciliberto, M. A., Sandige, H., Ndekha, M. J., Ashorn, P., Briend, A., Ciliberto, H. M., & Manary, M. J. (2005). Comparison of home-based therapy with ready-to-use therapeutic food with standard therapy in the treatment of malnourished Malawian children: a controlled, clinical effectiveness trial. Am J Clin Nutr, 81(4), 864-870. | 2005 | Journal article | | | | ✓ | |
| Coutinho, S. B., de Lira, P. I., de Carvalho Lima, M., & Ashworth, A. (2005). Comparison of the effect of two systems for the promotion of exclusive breastfeeding. Lancet, 366(9491), 1094-1100. doi:10.1016/S0140-6736(05)67421-1 | 2005 | Journal article | | ✓ | | | ✓ |
| Doocy, S., Teferra, S., Norell, D., & Burnham, G. (2005). Credit program outcomes: coping capacity and nutritional status in the food insecure context of Ethiopia. Soc Sci Med, 60(10), 2371-2382. doi:10.1016/j.socscimed.2004.10.025 | 2005 | Journal article | | | | ✓ | |
| Grabowsky, M., Farrell, N., Hawley, W., Chimumbwa, J., Hoyer, S., Wolkon, A., & Selanikio, J. (2005). Integrating insecticide-treated bednets into a measles vaccination campaign achieves high, rapid and equitable coverage with direct and voucher-based methods. Trop Med Int Health, 10(11), 1151-1160. doi:10.1111/j.1365-3156.2005.01502.x | 2005 | Journal article | | | ✓ | | ✓ |
| Hossain, S. M., Duffield, A., & Taylor, A. (2005). An evaluation of the impact of a US$60 million nutrition programme in Bangladesh. Health Policy Plan, 20(1), 35-40. doi:10.1093/heapol/czi004 | 2005 | Journal article | | | | ✓ | |
| Jokhio, A. H., Winter, H. R., & Cheng, K. K. (2005). An intervention involving traditional birth attendants and perinatal and maternal mortality in Pakistan. N Engl J Med, 352(20), 2091-2099. doi:10.1056/NEJMsa042830 | 2005 | Journal article | ✓ | ✓ | | | |
| Kagaayi, J., Dreyfuss, M. L., Kigozi, G., Chen, M. Z., Wabwire-Mangen, F., Serwadda, D., . . . Gray, R. H. (2005). Maternal self-medication and provision of nevirapine to newborns by women in Rakai, Uganda. J Acquir Immune Defic Syndr, 39(1), 121-124. doi:http://dx.doi.org/10.1097/01.qai.0000148530.66587.7c | 2005 | Journal article | | ✓ | ✓ | | |
| Luby, S. P., Agboatwalla, M., Feikin, D. R., Painter, J., Billhimer, W., Altaf, A., & Hoekstra, R. M. (2005). Effect of handwashing on child health: a randomised controlled trial. Lancet, 366(9481), 225-233. doi:10.1016/S0140-6736(05)66912-7 | 2005 | Journal article | | | ✓ | | |

## Chronological reference list: Assessments included in the CBPHC review

| Reference | Year | Reference type | Type of assessment carried out | | | | |
|---|---|---|---|---|---|---|---|
| | | | | Child health | | | |
| | | | Maternal health | Neonatal health | Health of children 1-59m | Health of 0-59-m-old children | Equity effect |
| Morrison, J., Tamang, S., Mesko, N., Osrin, D., Shrestha, B., Manandhar, M., . . . Costello, A. (2005). Women's health groups to improve perinatal care in rural Nepal. BMC Pregnancy Childbirth, 5(1), 6. doi:10.1186/1471-2393-5-6 | 2005 | Journal article | ✓ | | | | |
| Mustaphi, P., & Dobe, M. (2005). Positive deviance--the West Bengal experience. Indian J Public Health, 49(4), 207-213. | 2005 | Journal article | | | | ✓ | ✓ |
| Osrin, D., Vaidya, A., Shrestha, Y., Baniya, R. B., Manandhar, D. S., Adhikari, R. K., . . . Costello, A. M. (2005). Effects of antenatal multiple micronutrient supplementation on birthweight and gestational duration in Nepal: double-blind, randomised controlled trial. Lancet, 365(9463), 955-962. doi:10.1016/S0140-6736(05)71084-9 | 2005 | Journal article | | | | ✓ | |
| Paxman, J. M., Sayeed, A., Buxbaum, A., Huber, S. C., & Stover, C. (2005). The India Local Initiatives Program: a model for expanding reproductive and child health services. Stud Fam Plann, 36(3), 203-220. doi:http://dx.doi.org/10.1111/j.1728-4465.2005.00062.x | 2005 | Journal article | | | | ✓ | |
| Shamsuddin, L., Nahar, K., Nasrin, B., Nahar, S., Tamanna, S., Kabir, R. M., . . . Anwary, S. A. (2005). Use of parenteral magnesium sulphate in eclampsia and severe pre-eclampsia cases in a rural set up of Bangladesh. Bangladesh Med Res Counc Bull, 31(2), 75-82. | 2005 | Journal article | ✓ | | | | |
| Shetty, A. K., Mhazo, M., Moyo, S., von Lieven, A., Mateta, P., Katzenstein, D. A., . . . Bassett, M. T. (2005). The feasibility of voluntary counselling and HIV testing for pregnant women using community volunteers in Zimbabwe. Int J STD AIDS, 16(11), 755-759. doi:10.1258/095646205774763090 | 2005 | Journal article | ✓ | | | | |
| Thapa, S., Choe, M. K., & Retherford, R. D. (2005). Effects of vitamin A supplementation on child mortality: evidence from Nepal's 2001 Demographic and Health Survey. Trop Med Int Health, 10(8), 782-789. doi:10.1111/j.1365-3156.2005.01448.x | 2005 | Journal article | | | | ✓ | |
| Webster, J., Lines, J., Bruce, J., Armstrong Schellenberg, J. R., & Hanson, K. (2005). Which delivery systems reach the poor? A review of equity of coverage of ever-treated nets, never-treated nets, and immunisation to reduce child mortality in Africa. Lancet Infect Dis, 5(11), 709-717. doi:10.1016/S1473-3099(05)70269-3 | 2005 | Journal article | | | ✓ | | ✓ |
| Adventist Development Relief Agency/Cambodia. (2006). Child survival XVII - Final evaluation. | 2006 | Report | ✓ | | | ✓ | |
| Adventist Development Relief Agency/Nicaragua. (2006). Final Evaluation: Child Survival XVII. | 2006 | Report | ✓ | | | ✓ | |
| Ahrari, M., Houser, R. F., Yassin, S., Mogheez, M., Hussaini, Y., Crump, P., . . . Levinson, F. J. (2006). A positive deviance-based antenatal nutrition project improves birth-weight in Upper Egypt. J Health Popul Nutr, 24(4), 498-507. | 2006 | Journal article | | ✓ | | | |

| Chronological reference list: Assessments included in the CBPHC review | | | | | | | |
|---|---|---|---|---|---|---|---|
| | | | Type of assessment carried out | | | | |
| | | | | Child health | | | |
| Reference | Year | Reference type | Mater-nal health | Neo-natal health | Health of children 1-59m | Health of 0-59-m-old children | Equity effect |
| Baker, E. J., Sanei, L. C., & Franklin, N. (2006). Early initiation of and exclusive breastfeeding in large-scale community-based programmes in Bolivia and Madagascar. J Health Popul Nutr, 24(4), 530-539. | 2006 | Journal article | | | | ✓ | |
| Bari, S., Mannan, I., Rahman, M. A., Darmstadt, G. L., Serajil, M. H., Baqui, A. H., . . . Bangladesh Projahnmo, I. I. S. G. (2006). Trends in use of referral hospital services for care of sick newborns in a community-based intervention in Tangail District, Bangladesh. J Health Popul Nutr, 24(4), 519-529. | 2006 | Journal article | | | | ✓ | |
| Bawah, A. A., Philips, J. F., Adjuik, M., Vaughan-Smith, M., Macleod, B., & Binka, F. N. (2006). The impact of immunization on the association between poverty and child survival: evidence from Kassena-Nankana district of northern Ghana. Population Council Working Papers 2/8. New York, NY | 2006 | Report | | | | ✓ | ✓ |
| Care/Mozambique. (2006). Child survial project: Final evaluation. | 2006 | Report | ✓ | | | ✓ | |
| Chaiken, M. S., Deconinck, H., & Degefie, T. (2006). The promise of a community-based approach to managing severe malnutrition: A case study from Ethiopia. Food and Nutrition Bulletin, 27(2), 95-103. doi:http://dx.doi.org/10.1177/156482650602700201 | 2006 | Journal article | | | | ✓ | |
| Derman, R. J., Kodkany, B. S., Goudar, S. S., Geller, S. E., Naik, V. A., Bellad, M. B., . . . Moss, N. (2006). Oral misoprostol in preventing postpartum haemorrhage in resource-poor communities: a randomised controlled trial. Lancet, 368(9543), 1248-1253. doi:10.1016/S0140-6736(06)69522-6 | 2006 | Journal article | ✓ | | | | |
| Edwards, N. C., & Roelofs, S. M. (2006). Sustainability: the elusive dimension of international health projects. Can J Public Health, 97(1), 45-49. | 2006 | Journal article | | | | ✓ | |
| Future Generations/Afghanistan (2006). Community health worker training for women's empowerment in Afghanistan: Summary report. | 2006 | Report | ✓ | | | ✓ | |
| Future Generations/Peru (2007). Internship Center Pilot Project The CLAS Las Moras-Huanuco: Mid-term evaluation. | 2006 | Report | | | | ✓ | |
| Hale, L., DaVanzo, J., Razzaque, A., & Rahman, M. (2006). Why are infant and child mortality rates lower in the MCH-FP area of Matlab, Bangladesh? Stud Fam Plann, 37(4), 281-292. doi:http://dx.doi.org/10.1111/j.1728-4465.2006.00106.x | 2006 | Journal article | | | | ✓ | |
| Khandekar, R., Ton, T. K., & Do Thi, P. (2006). Impact of face washing and environmental improvement on reduction of active trachoma in Vietnam-a public health intervention study. Ophthalmic Epidemiol, 13(1), 43-52. doi:10.1080/09286580500477507 | 2006 | Journal article | | | | ✓ | |
| Larocque, R., Casapia, M., Gotuzzo, E., MacLean, J. D., Soto, J. C., Rahme, E., & Gyorkos, T. W. (2006). A double-blind randomized controlled trial of antenatal mebendazole to reduce low birthweight in a hookworm-endemic area of Peru. Trop Med Int | 2006 | Journal article | ✓ | ✓ | | | |

| Chronological reference list: Assessments included in the CBPHC review | | | | | | | |
|---|---|---|---|---|---|---|---|
| | | | Type of assessment carried out | | | | |
| | | | | Child health | | | |
| Reference | Year | Reference type | Mater-nal health | Neo-natal health | Health of children 1-59m | Health of 0-59-m-old children | Equity effect |
| Health, 11(10), 1485-1495. doi:10.1111/j.1365-3156.2006.01706.x | | | | | | | |
| Management Sciences for Health/REACH (2006). Rural expansion of Afganistan's community-based healthcare program: Measuring program outcomes through household surveys. | 2006 | Report | | | | ✓ | |
| McPherson, R. A., Khadka, N., Moore, J. M., & Sharma, M. (2006). Are birth-preparedness programmes effective? Results from a field trial in Siraha district, Nepal. J Health Popul Nutr, 24(4), 479-488. | 2006 | Journal article | ✓ | | | | |
| Mullany, L. C., Darmstadt, G. L., Khatry, S. K., Katz, J., LeClerq, S. C., Shrestha, S., . . . Tielsch, J. M. (2006). Topical applications of chlorhexidine to the umbilical cord for prevention of omphalitis and neonatal mortality in southern Nepal: a community-based, cluster-randomised trial. Lancet, 367(9514), 910-918. doi:10.1016/S0140-6736(06)68381-5 | 2006 | Journal article | | ✓ | | | |
| Muller, O., Traore, C., Kouyate, B., Ye, Y., Frey, C., Coulibaly, B., & Becher, H. (2006). Effects of insecticide-treated bednets during early infancy in an African area of intense malaria transmission: a randomized controlled trial. Bull World Health Organ, 84(2), 120-126. doi:/S0042-96862006000200012 | 2006 | Journal article | | | | ✓ | |
| Perry, H., Cayemittes, M., Philippe, F., Dowell, D., Dortonne, J. R., Menager, H., . . . Berggren, G. (2006). Reducing under-five mortality through Hopital Albert Schweitzer's integrated system in Haiti. Health Policy Plan, 21(3), 217-230. doi:10.1093/heapol/czl005 | 2006 | Journal article | | ✓ | ✓ | | ✓ |
| Phillips, J. F., Bawah, A. A., & Binka, F. N. (2006). Accelerating reproductive and child health programme impact with community-based services: the Navrongo experiment in Ghana. Bull World Health Organ, 84(12), 949-955. doi:http://dx.doi.org/10.2471/blt.06.030064 | 2006 | Journal article | | | | ✓ | |
| Plan/Mali. (2006). Child Reach: Child Survival XVII Project, Kita District, Kayes region, Mali. | 2006 | Report | | | | ✓ | |
| Plan/Nepal (2006). For our mothers and children: Scaling success to the district level Plan Nepal Child Survival XVIII. | 2006 | Report | ✓ | ✓ | | | |
| SANRU III (2006). Final Evaluation. | 2006 | Report | ✓ | | | ✓ | |
| Save the Children/Ethiopia (2006). Essential Services for Maternal and Child Survival in Ethiopia: Mobilizing the Traditional and Public Health Sectors and Informing Programming for Pastoralist Populations. | 2006 | Report | ✓ | | | ✓ | |
| Save the Children/Guinea (2006). Child Survival 18-Guinea final evaluation report: Community health Initiative for the districts of Kouroussa and Mandiana Guinea. | 2006 | Report | ✓ | | | ✓ | |
| Sazawal, S., Black, R. E., Ramsan, M., Chwaya, H. M., Stoltzfus, R. J., Dutta, A., . . . Kabole, F. M. (2006). Effects of routine prophylactic supplementation with iron and folic acid on admission to hospital and mortality in preschool children in a high malaria transmission setting: community-based, randomised, | 2006 | Journal article | | | | ✓ | |

## Chronological reference list: Assessments included in the CBPHC review

| Reference | Year | Reference type | Type of assessment carried out | | | | |
|---|---|---|---|---|---|---|---|
| | | | | Child health | | | |
| | | | Maternal health | Neonatal health | Health of children 1-59m | Health of 0-59-m-old children | Equity effect |
| placebo-controlled trial. Lancet, 367(9505), 133-143. doi:10.1016/S0140-6736(06)67962-2 | | | | | | | |
| Sepulveda, J., Bustreo, F., Tapia, R., Rivera, J., Lozano, R., Olaiz, G., . . . Valdespino, J. L. (2006). Improvement of child survival in Mexico: the diagonal approach. Lancet, 368(9551), 2017-2027. doi:10.1016/S0140-6736(06)69569-X | 2006 | Journal article | | | | ✓ | ✓ |
| Syed, U., Asiruddin, S., Helal, M. S., Mannan, II, & Murray, J. (2006). Immediate and early postnatal care for mothers and newborns in rural Bangladesh. J Health Popul Nutr, 24(4), 508-518. | 2006 | Journal article | ✓ | ✓ | | | |
| Tielsch, J. M., Khatry, S. K., Stoltzfus, R. J., Katz, J., LeClerq, S. C., Adhikari, R., . . . Black, R. E. (2006). Effect of routine prophylactic supplementation with iron and folic acid on preschool child mortality in southern Nepal: community-based, cluster-randomised, placebo-controlled trial. Lancet, 367(9505), 144-152. doi:10.1016/S0140-6736(06)67963-4 | 2006 | Journal article | | | | ✓ | |
| World Relief/Rwanda (2006). Rwanda "Umucyo" (Illumination) Child Survival project: Final evaluation report. | 2006 | Report | | | | ✓ | |
| AbdelRahman, S. H., Albashir, I. M., Hussein, S. A., Ahmed, M. E., Alfadil, S. M., & Mohamed, N. (2007). BDN programmes and the effect of medical students' interventions to promote child health in Sudan. East Mediterr Health J, 13(6), 1319-1329. | 2007 | Journal article | ✓ | | | ✓ | |
| Adu-Afarwuah, S., Lartey, A., Brown, K. H., Zlotkin, S., Briend, A., & Dewey, K. G. (2007). Randomized comparison of 3 types of micronutrient supplements for home fortification of complementary foods in Ghana: effects on growth and motor development. Am J Clin Nutr, 86(2), 412-420. | 2007 | Journal article | | | | ✓ | |
| Aghajanian, A., Mehryar, A. H., Ahmadnia, S., & Kazemipour, S. (2007). Impact of rural health development programme in the Islamic Republic of Iran on rural-urban disparities in health indicators. East Mediterr Health J, 13(6), 1466-1475. | 2007 | Journal article | ✓ | | | | |
| Alderman, H. (2007). Improving nutrition through community growth promotion: Longitudinal study of the nutrition and early child development program in Uganda. World Development, 35(8), 1376-1389. doi:http://dx.doi.org/10.1016/j.worlddev.2007.04.003 | 2007 | Journal article | | | | ✓ | |
| Baek, C., Mathambo, V., Mkhize, S., Friedman, I., Apicella, L., & Rutenberg, N. (2007). Key findings from an evaluation of the mother2mothers programs in KwaZulu-Natal, South Africa. | 2007 | Report | | | | ✓ | |
| Barreto, M. L., Genser, B., Strina, A., Teixeira, M. G., Assis, A. M., Rego, R. F., . . . Cairncross, S. (2007). Effect of city-wide sanitation programme on reduction in rate of childhood diarrhoea in northeast Brazil: assessment by two cohort studies. Lancet, 370(9599), 1622-1628. doi:10.1016/S0140-6736(07)61638-9 | 2007 | Journal article | | | | ✓ | ✓ |

| Chronological reference list: Assessments included in the CBPHC review | | | | | | | |
|---|---|---|---|---|---|---|---|
| | | | Type of assessment carried out | | | | |
| | | | | Child health | | | |
| Reference | Year | Reference type | Maternal health | Neonatal health | Health of children 1-59m | Health of 0-59-m-old children | Equity effect |
| Basics/Rwanda. (2007). External evaluation of the pilot phase of the home-based management of malaria program in Rwanda: Final report. | 2007 | Report | | | | ✓ | |
| Binka, F. N., Bawah, A. A., Phillips, J. F., Hodgson, A., Adjuik, M., & MacLeod, B. (2007). Rapid achievement of the child survival millennium development goal: evidence from the Navrongo experiment in Northern Ghana. Trop Med Int Health, 12(5), 578-583. doi:10.1111/j.1365-3156.2007.01826.x | 2007 | Journal article | | | | ✓ | |
| Care/Ethiopia. (2007). Farta child survival project, Amhara National state, South Gondar administrative zone, Farta Woreda, Ethiopia. | 2007 | Report | | | | ✓ | |
| CB-MNC Nepal (2007). Community-based maternal and neonatal care program (CB-MNC): Summative report on program activities and results in Banka, Jhapa and Kanchanpur districts from September 2005 - September 2007. | 2007 | Report | ✓ | | | ✓ | |
| Collins, S. (2007). Treating severe acute malnutrition seriously. Arch Dis Child, 92(5), 453-461. doi:10.1136/adc.2006.098327 | 2007 | Journal article | | | | ✓ | |
| Crookston, B. T., Dearden, K. A., Chan, K., Chan, T., & Stoker, D. D. (2007). Buddhist nuns on the move: an innovative approach to improving breastfeeding practices in Cambodia. Matern Child Nutr, 3(1), 10-24. doi:10.1111/j.1740-8709.2007.00074.x | 2007 | Journal article | | ✓ | | | ✓ |
| Curamericas/Bolivia. (2007). Providing child survival services to rural and peri-urban populations in Bolivia - Final Evaluation Report. | 2007 | Report | ✓ | | | ✓ | |
| Curamericas/Guatemala (2007). Census-Based, Impact-Oriented Child Survival Project. October 1, 2002 - September 30, 2007: Final Evaluation Report | 2007 | Report | ✓ | | | ✓ | |
| Dubowitz, T., Levinson, D., Peterman, J. N., Verma, G., Jacob, S., & Schultink, W. (2007). Intensifying efforts to reduce child malnutrition in India: an evaluation of the Dular program in Jharkhand, India. Food Nutr Bull, 28(3), 266-273. doi:http://dx.doi.org/10.1177/156482650702800302 | 2007 | Journal article | | | | ✓ | |
| Edward, A., Ernst, P., Taylor, C., Becker, S., Mazive, E., & Perry, H. (2007). Examining the evidence of under-five mortality reduction in a community-based programme in Gaza, Mozambique. Trans R Soc Trop Med Hyg, 101(8), 814-822. doi:10.1016/j.trstmh.2007.02.025 | 2007 | Journal article | | | | ✓ | |
| Eklund, P., Imai, K., & Felloni, F. (2007). Women's organizations, maternal knowledge, and social capital to reduce prevalence of stunted children: evidence from rural Nepal. Journal of Development Studies, 43(3), 456-489. doi:http://dx.doi.org/10.1080/00220380701204406 | 2007 | Journal article | | | | ✓ | |
| Vision Eritrea & The Swiss Red Cross. (2007). Community based health care project, Eritrea (2003-2007): Final Evaluation Report. | 2007 | Report | ✓ | | | | |
| Fegan, G. W., Noor, A. M., Akhwale, W. S., Cousens, S., & Snow, R. W. (2007). Effect of expanded insecticide-treated bednet coverage on child survival in rural | 2007 | Journal article | | | ✓ | | ✓ |

| Chronological reference list: Assessments included in the CBPHC review | | | | | | | |
|---|---|---|---|---|---|---|---|
| | | | Type of assessment carried out | | | | |
| | | | | Child health | | | |
| Reference | Year | Reference type | Mater-nal health | Neo-natal health | Health of children 1-59m | Health of 0-59-m-old children | Equity effect |
| Kenya: a longitudinal study. Lancet, 370(9592), 1035-1039. doi:10.1016/S0140-6736(07)61477-9 | | | | | | | |
| Future Generations/Peru (2007). CLAS Las Moras: Intermediate Evaluation. | 2007 | Report | ✓ | | | | |
| Gottlieb, J. (2007). Reducing child mortality with Vitamin A in Nepal.Case Studies in Global Health: Millions Saved. Levine, R (ed.) 2007 Jones and Bartlett | 2007 | Report | | | | ✓ | |
| Grobusch, M. P., Lell, B., Schwarz, N. G., Gabor, J., Dornemann, J., Potschke, M., . . . Kremsner, P. G. (2007). Intermittent preventive treatment against malaria in infants in Gabon--a randomized, double-blind, placebo-controlled trial. J Infect Dis, 196(11), 1595-1602. doi:10.1086/522160 | 2007 | Journal article | | | | ✓ | |
| Gupta, D. N., Rajendran, K., Mondal, S. K., Ghosh, S., & Bhattacharya, S. K. (2007). Operational feasibility of implementing community-based zinc supplementation: impact on childhood diarrheal morbidity. Pediatr Infect Dis J, 26(4), 306-310. doi:10.1097/01.inf.0000258692.65485.d9 | 2007 | Journal article | | | ✓ | | |
| Houeto, D., & Deccache, A. (2007). Child malaria in sub-saharan Africa: effective control and prevention require a health promotion approach. Int Q Community Health Educ, 28(1), 51-62. doi:10.2190/IQ.28.1.e | 2007 | Journal article | | | ✓ | | |
| Levinson, F. J., Barney, J., Bassett, L., & Schultink, W. (2007). Utilization of positive deviance analysis in evaluating community-based nutrition programs: an application to the Dular program in Bihar, India. Food Nutr Bull, 28(3), 259-265. doi:http://dx.doi.org/10.1177/156482650702800301 | 2007 | Journal article | | | | ✓ | |
| Li, Y., Hotta, M., Shi, A., Li, Z., Yin, J., Guo, G., . . . Ushijima, H. (2007). Malnutrition improvement for infants under 18 months old of Dai minority in Luxi, China. Pediatr Int, 49(2), 273-279. doi:10.1111/j.1442-200X.2007.02349.x | 2007 | Journal article | | ✓ | | | |
| Luabeya, K. K., Mpontshane, N., Mackay, M., Ward, H., Elson, I., Chhagan, M., . . . Bennish, M. L. (2007). Zinc or multiple micronutrient supplementation to reduce diarrhea and respiratory disease in South African children: a randomized controlled trial. PLoS One, 2(6), e541. doi:10.1371/journal.pone.0000541 | 2007 | Journal article | | | | ✓ | |
| Mbonye, A. K., Bygbjerg, I., & Magnussen, P. (2007). Intermittent preventive treatment of malaria in pregnancy: evaluation of a new delivery approach and the policy implications for malaria control in Uganda. Health Policy, 81(2-3), 228-241. doi:10.1016/j.healthpol.2006.05.018 | 2007 | Journal article | ✓ | | | ✓ | |
| Migele, J., Ombeki, S., Ayalo, M., Biggerstaff, M., & Quick, R. (2007). Diarrhea prevention in a Kenyan school through the use of a simple safe water and hygiene intervention. Am J Trop Med Hyg, 76(2), 351-353. | 2007 | Journal article | | | | ✓ | |

## Chronological reference list: Assessments included in the CBPHC review

| | | | Type of assessment carried out | | | | |
|---|---|---|---|---|---|---|---|
| | | | | Child health | | | |
| **Reference** | **Year** | **Reference type** | **Maternal health** | **Neonatal health** | **Health of children 1-59m** | **Health of 0-59-m-old children** | **Equity effect** |
| Mockenhaupt, F. P., Reither, K., Zanger, P., Roepcke, F., Danquah, I., Saad, E., . . . Bienzle, U. (2007). Intermittent preventive treatment in infants as a means of malaria control: a randomized, double-blind, placebo-controlled trial in northern Ghana. Antimicrob Agents Chemother, 51(9), 3273-3281. doi:10.1128/AAC.00513-07 | 2007 | Journal article | | | ✓ | | |
| Noor, A. M., Amin, A. A., Akhwale, W. S., & Snow, R. W. (2007). Increasing coverage and decreasing inequity in insecticide-treated bed net use among rural Kenyan children. PLoS Med, 4(8), e255. doi:10.1371/journal.pmed.0040255 | 2007 | Journal article | | | | ✓ | ✓ |
| Owusu-Agyei, S., Awini, E., Anto, F., Mensah-Afful, T., Adjuik, M., Hodgson, A., . . . Binka, F. (2007). Assessing malaria control in the Kassena-Nankana district of northern Ghana through repeated surveys using the RBM tools. Malar J, 6, 103. doi:10.1186/1475-2875-6-103 | 2007 | Journal article | ✓ | | | | |
| Paxson, C., & Schady, N. (2007). Does money matter?: The effects of cash transfers on child health and development in rural Ecuador. Retrieved from World Bank Policy Research Working Paper 4426: | 2007 | Report | | | | ✓ | |
| Pence, B. W., Nyarko, P., Phillips, J. F., & Debpuur, C. (2007). The effect of community nurses and health volunteers on child mortality: the Navrongo Community Health and Family Planning Project. Scand J Public Health, 35(6), 599-608. doi:10.1080/14034940701349225 | 2007 | Journal article | | | ✓ | | |
| Razzaque, A., Streatfield, P. K., & Gwatkin, D. R. (2007). Does health intervention improve socioeconomic inequalities of neonatal, infant and child mortality? Evidence from Matlab, Bangladesh. Int J Equity Health, 6, 4. doi:10.1186/1475-9276-6-4 | 2007 | Journal article | | | | ✓ | ✓ |
| Roy, S. K., Jolly, S. P., Shafique, S., Fuchs, G. J., Mahmud, Z., Chakraborty, B., & Roy, S. (2007). Prevention of malnutrition among young children in rural Bangladesh by a food-health-care educational intervention: a randomized, controlled trial. Food Nutr Bull, 28(4), 375-383. doi:http://dx.doi.org/10.1177/156482650702800401 | 2007 | Journal article | | | ✓ | | |
| Sadler, K., Myatt, M., Feleke, T., & Collins, S. (2007). A comparison of the programme coverage of two therapeutic feeding interventions implemented in neighbouring districts of Malawi. Public Health Nutr, 10(9), 907-913. doi:10.1017/S1368980007711035 | 2007 | Journal article | | | | ✓ | |
| Sazawal, S., Black, R. E., Ramsan, M., Chwaya, H. M., Dutta, A., Dhingra, U., . . . Kabole, F. M. (2007). Effect of zinc supplementation on mortality in children aged 1-48 months: a community-based randomised placebo-controlled trial. Lancet, 369(9565), 927-934. doi:10.1016/S0140-6736(07)60452-8 | 2007 | Journal article | | | | ✓ | |
| Schemann, J. F., Guinot, C., Traore, L., Zefack, G., Dembele, M., Diallo, I., . . . Malvy, D. (2007). Longitudinal evaluation of three azithromycin distribution strategies for treatment of trachoma in a | 2007 | Journal article | | | | ✓ | |

| Chronological reference list: Assessments included in the CBPHC review | | | | | | | |
|---|---|---|---|---|---|---|---|
| | | | Type of assessment carried out | | | | |
| | | | | Child health | | | |
| Reference | Year | Reference type | Mater-nal health | Neo-natal health | Health of children 1-59m | Health of 0-59-m-old children | Equity effect |
| sub-Saharan African country, Mali. Acta Trop, 101(1), 40-53. doi:10.1016/j.actatropica.2006.12.003 | | | | | | | |
| Schultink, W., Mitra, K., & Mustaphi, P. (2007). Cost-effective reduction in under nutrition in India through community based interventions. UNICEF. | 2007 | Report | | | | ✓ | |
| Skarbinski, J., Massaga, J. J., Rowe, A. K., & Kachur, S. P. (2007). Distribution of free untreated bednets bundled with insecticide via an integrated child health campaign in Lindi Region, Tanzania: lessons for future campaigns. Am J Trop Med Hyg, 76(6), 1100-1106. | 2007 | Journal article | | | ✓ | | ✓ |
| Tielsch, J. M., Darmstadt, G. L., Mullany, L. C., Khatry, S. K., Katz, J., LeClerq, S. C., . . . Adhikari, R. (2007). Impact of newborn skin-cleansing with chlorhexidine on neonatal mortality in southern Nepal: a community-based, cluster-randomized trial. Pediatrics, 119(2), e330-340. doi:10.1542/peds.2006-1192 | 2007 | Journal article | | ✓ | | | |
| Tielsch, J. M., Khatry, S. K., Stoltzfus, R. J., Katz, J., LeClerq, S. C., Adhikari, R., . . . Shresta, S. (2007). Effect of daily zinc supplementation on child mortality in southern Nepal: a community-based, cluster randomised, placebo-controlled trial. Lancet, 370(9594), 1230-1239. doi:10.1016/S0140-6736(07)61539-6 | 2007 | Journal article | | | | ✓ | |
| Tielsch, J. M., Rahmathullah, L., Thulasiraj, R. D., Katz, J., Coles, C., Sheeladevi, S., . . . Prakash, K. (2007). Newborn vitamin A dosing reduces the case fatality but not incidence of common childhood morbidities in South India. J Nutr, 137(11), 2470-2474. | 2007 | Journal article | | | | ✓ | |
| UNICEF/Bihar & Jharkhand. (2007). Dular: An integrated community based child development and nutrition project Bihar & Jharkhand India. | 2007 | Report | | | | ✓ | |
| UNICEF/Rajasthan. (2007). Anchal se angan tak: Community-based integrated nutrition strategy, Rajasthan. | 2007 | Report | | | | ✓ | |
| Ajayi, I. O., Browne, E. N., Garshong, B., Bateganya, F., Yusuf, B., Agyei-Baffour, P., . . . Pagnoni, F. (2008). Feasibility and acceptability of artemisinin-based combination therapy for the home management of malaria in four African sites. Malar J, 7, 6. doi:10.1186/1475-2875-7-6 | 2008 | Journal article | | | ✓ | | |
| Alderman, H., Ndiaye, B., Linnemayr, S., Ka, A., Rokx, C., Dieng, K., & Mulder-Sibanda, M. (2008). Effectiveness of a community-based intervention to improve nutrition in young children in Senegal: a difference in difference analysis. Public Health Nutr, 12(5), 667-673. doi:10.1017/S1368980008002619 | 2008 | Journal article | | | ✓ | | |
| Asling-Monemi, K., Tabassum Naved, R., & Persson, L. A. (2008). Violence against women and the risk of under-five mortality: analysis of community-based data from rural Bangladesh. Acta Paediatr, 97(2), 226-232. doi:10.1111/j.1651-2227.2007.00597.x | 2008 | Journal article | | | | ✓ | |

## Chronological reference list: Assessments included in the CBPHC review

| Reference | Year | Reference type | Type of assessment carried out | | | | |
|---|---|---|---|---|---|---|---|
| | | | | Child health | | | |
| | | | Mater-nal health | Neo-natal health | Health of children 1-59m | Health of 0-59-m-old children | Equity effect |
| Baqui, A. H., El-Arifeen, S., Darmstadt, G. L., Ahmed, S., Williams, E. K., Seraji, H. R., . . . Projahnmo Study, G. (2008). Effect of community-based newborn-care intervention package implemented through two service-delivery strategies in Sylhet district, Bangladesh: a cluster-randomised controlled trial. Lancet, 371(9628), 1936-1944. doi:10.1016/S0140-6736(08)60835-1 | 2008 | Journal article | ✓ | | | ✓ | |
| Baqui, A. H., Rosecrans, A. M., Williams, E. K., Agrawal, P. K., Ahmed, S., Darmstadt, G. L., . . . Santosham, M. (2008). NGO facilitation of a government community-based maternal and neonatal health programme in rural India: improvements in equity. Health Policy Plan, 23(4), 234-243. doi:10.1093/heapol/czn012 | 2008 | Journal article | ✓ | ✓ | | | ✓ |
| Baqui, A., Williams, E. K., Rosecrans, A. M., Agrawal, P. K., Ahmed, S., Darmstadt, G. L., . . . Santosham, M. (2008). Impact of an integrated nutrition and health programme on neonatal mortality in rural northern India. Bull World Health Organ, 86(10), 796-804, A. doi:http://dx.doi.org/10.2471/blt.07.042226 | 2008 | Journal article | ✓ | | | ✓ | |
| Bashour, H. N., Kharouf, M. H., Abdulsalam, A. A., El Asmar, K., Tabbaa, M. A., & Cheikha, S. A. (2008). Effect of postnatal home visits on maternal/infant outcomes in Syria: a randomized controlled trial. Public Health Nurs, 25(2), 115-125. doi:10.1111/j.1525-1446.2008.00688.x | 2008 | Journal article | ✓ | | | ✓ | |
| Benn, C. S., Diness, B. R., Roth, A., Nante, E., Fisker, A. B., Lisse, I. M., . . . Aaby, P. (2008). Effect of 50,000 IU vitamin A given with BCG vaccine on mortality in infants in Guinea-Bissau: randomised placebo controlled trial. BMJ, 336(7658), 1416-1420. doi:10.1136/bmj.39542.509444.AE | 2008 | Journal article | | | | ✓ | |
| Bhandari, N., Mazumder, S., Taneja, S., Dube, B., Agarwal, R. C., Mahalanabis, D., . . . Bhan, M. K. (2008). Effectiveness of zinc supplementation plus oral rehydration salts compared with oral rehydration salts alone as a treatment for acute diarrhea in a primary care setting: a cluster randomized trial. Pediatrics, 121(5), e1279-1285. doi:10.1542/peds.2007-1939 | 2008 | Journal article | | | ✓ | | |
| Bhutta, Z. A., Memon, Z. A., Soofi, S., Salat, M. S., Cousens, S., & Martines, J. (2008). Implementing community-based perinatal care: results from a pilot study in rural Pakistan. Bull World Health Organ, 86(6), 452-459. doi:http://dx.doi.org/10.2471/blt.07.045849 | 2008 | Journal article | ✓ | | | ✓ | |
| Bryce, J., Gilroy, K., Jones, G., Hazel, E., Black, R. E., & Victora, C. G. (2008). The Retrospective Evaluation of ACSD: Cross-site analyses and conclusions. | 2008 | Report | ✓ | | | ✓ | ✓ |
| Care/Nicaragua (2008). Child Survival Project (CSP) XVIII: Phase two - Final Evaluation. | 2008 | Report | ✓ | | | ✓ | |
| Cesar, J. A., Mendoza-Sassi, R. A., Ulmi, E. F., Dall'Agnol, M. M., & Neumann, N. A. (2008). [Effects of different home visit strategies on prenatal care in Southern Brazil]. Cad Saude Publica, 24(11), 2614- | 2008 | Journal article | ✓ | | | ✓ | |

| Chronological reference list: Assessments included in the CBPHC review | | | | | | | | |
|---|---|---|---|---|---|---|---|---|
| | | | Type of assessment carried out | | | | | |
| | | | | Child health | | | | |
| Reference | Year | Reference type | Mater-nal health | Neo-natal health | Health of children 1-59m | Health of 0-59-m-old children | Equity effect |
| 2622. doi:http://dx.doi.org/10.1590/s0102-311x2008001100016 | | | | | | | |
| Concern Worldwide/Bangladesh (2008). The end of magical thinking: Sustainability evaluation three years after the end of the Saidpur and Parbatipur urban health project. | 2008 | Report | | | | ✓ | |
| Cumberland, P., Edwards, T., Hailu, G., Harding-Esch, E., Andreasen, A., Mabey, D., & Todd, J. (2008). The impact of community level treatment and preventative interventions on trachoma prevalence in rural Ethiopia. Int J Epidemiol, 37(3), 549-558. doi:10.1093/ije/dyn045 | 2008 | Journal article | | | | ✓ | ✓ |
| Das, L. K., Jambulingam, P., & Sadanandane, C. (2008). Impact of community-based presumptive chloroquine treatment of fever cases on malaria morbidity and mortality in a tribal area in Orissa State, India. Malar J, 7, 75. doi:10.1186/1475-2875-7-75 | 2008 | Journal article | | | | ✓ | |
| Dawson, P., Pradhan, Y., Houston, R., Karki, S., Poudel, D., & Hodgins, S. (2008). From research to national expansion: 20 years' experience of community-based management of childhood pneumonia in Nepal. Bull World Health Organ, 86(5), 339-343. doi:http://dx.doi.org/10.2471/blt.07.047688 | 2008 | Journal article | | | | ✓ | |
| Fernald, L. C., Gertler, P. J., & Neufeld, L. M. (2008). Role of cash in conditional cash transfer programmes for child health, growth, and development: an analysis of Mexico's Oportunidades. Lancet, 371(9615), 828-837. doi:10.1016/S0140-6736(08)60382-7 | 2008 | Journal article | | | | ✓ | ✓ |
| Fiedler, J. L., & Chuko, T. (2008). The cost of Child Health Days: a case study of Ethiopia's Enhanced Outreach Strategy (EOS). Health Policy Plan, 23(4), 222-233. doi:10.1093/heapol/czn015 | 2008 | Journal article | | | | ✓ | |
| Food for the Hungry/Mozambique (2008). Achieving equity, coverge, and impact through a care group network, Mozambique, Sofala Province. | 2008 | Report | | | | ✓ | |
| Harkins, T., Drasbek, C., Arroyo, J., & McQuestion, M. (2008). The health benefits of social mobilization: experiences with community-based Integrated Management of Childhood Illness in Chao, Peru and San Luis, Honduras. Promot Educ, 15(2), 15-20. doi:10.1177/1025382308090340 | 2008 | Journal article | | | | ✓ | |
| Jakobsen, M. S., Sodemann, M., Biai, S., Nielsen, J., & Aaby, P. (2008). Promotion of exclusive breastfeeding is not likely to be cost effective in West Africa. A randomized intervention study from Guinea-Bissau. Acta Paediatr, 97(1), 68-75. doi:10.1111/j.1651-2227.2007.00532.x | 2008 | Journal article | | | | ✓ | |
| Kisinza, W. N., Kisoka, W. J., Mutalemwa, P. P., Njau, J., Tenu, F., Nkya, T., . . . Magesa, S. M. (2008). Community directed interventions for malaria, tuberculosis and vitamin A in onchocerciasis endemic districts of Tanzania. Tanzan J Health Res, 10(4), 232-239. doi:http://dx.doi.org/10.4314/thrb.v10i4.45079 | 2008 | Journal article | | | | ✓ | |
| Klemm, R. D., Labrique, A. B., Christian, P., Rashid, M., Shamim, A. A., Katz, J., . . . West, K. P., Jr. (2008). | 2008 | Journal article | | | | ✓ | |

**Chronological reference list: Assessments included in the CBPHC review**

| Reference | Year | Reference type | Type of assessment carried out | | | | |
|---|---|---|---|---|---|---|---|
| | | | | Child health | | | |
| | | | Mater-nal health | Neo-natal health | Health of children 1-59m | Health of 0-59-m-old children | Equity effect |
| Newborn vitamin A supplementation reduced infant mortality in rural Bangladesh. Pediatrics, 122(1), e242-250. doi:10.1542/peds.2007-3448 | | | | | | | |
| Kouyate, B., Some, F., Jahn, A., Coulibaly, B., Eriksen, J., Sauerborn, R., . . . Mueller, O. (2008). Process and effects of a community intervention on malaria in rural Burkina Faso: randomized controlled trial. Malar J, 7, 50. doi:10.1186/1475-2875-7-50 | 2008 | Journal article | | | ✓ | | |
| Kumar, V., Mohanty, S., Kumar, A., Misra, R. P., Santosham, M., Awasthi, S., . . . Saksham Study, G. (2008). Effect of community-based behaviour change management on neonatal mortality in Shivgarh, Uttar Pradesh, India: a cluster-randomised controlled trial. Lancet, 372(9644), 1151-1162. doi:10.1016/S0140-6736(08)61483-X | 2008 | Journal article | | ✓ | | | ✓ |
| Care/Sierra Leone (2008). Child survival project 'For Di Pikin Dem Wel Bodi', Koinadugu District, Northern Region, Sierra Leone: Final evaluation. | 2008 | Report | ✓ | | | | |
| Lutter, C. K., Rodriguez, A., Fuenmayor, G., Avila, L., Sempertegui, F., & Escobar, J. (2008). Growth and micronutrient status in children receiving a fortified complementary food. J Nutr, 138(2), 379-388. | 2008 | Journal article | | | | ✓ | ✓ |
| Asha/India (2008). Overview | 2008 | Report | ✓ | | | ✓ | ✓ |
| Mbonye, A. K., Bygbjerg, I. C., & Magnussen, P. (2008). Intermittent preventive treatment of malaria in pregnancy: a new delivery system and its effect on maternal health and pregnancy outcomes in Uganda. Bull World Health Organ, 86(2), 93-100. doi:http://dx.doi.org/10.2471/blt.07.041822 | 2008 | Journal article | ✓ | | | ✓ | |
| Mbonye, A. K., Bygbjerg, I., & Magnussen, P. (2008). Intermittent preventive treatment of malaria in pregnancy: a community-based delivery system and its effect on parasitemia, anemia and low birth weight in Uganda. Int J Infect Dis, 12(1), 22-29. doi:10.1016/j.ijid.2006.10.008 | 2008 | Journal article | ✓ | | | | |
| Mbonye, A. K., Hansen, K. S., Bygbjerg, I. C., & Magnussen, P. (2008). Intermittent preventive treatment of malaria in pregnancy: the incremental cost-effectiveness of a new delivery system in Uganda. Trans R Soc Trop Med Hyg, 102(7), 685-693. doi:10.1016/j.trstmh.2008.04.016 | 2008 | Journal article | ✓ | | | ✓ | ✓ |
| Mbonye, A. K., Schultz Hansen, K., Bygbjerg, I. C., & Magnussen, P. (2008). Effect of a community-based delivery of intermittent preventive treatment of malaria in pregnancy on treatment seeking for malaria at health units in Uganda. Public Health, 122(5), 516-525. doi:10.1016/j.puhe.2007.07.024 | 2008 | Journal article | ✓ | | | ✓ | |
| Medical Team International/Liberia (2008). Grand Cape Mount child survival project: Improved child health in a transitional state through IMCI. | 2008 | Report | | | | ✓ | |
| Mermin, J., Were, W., Ekwaru, J. P., Moore, D., Downing, R., Behumbiize, P., . . . Bunnell, R. (2008). Mortality in HIV-infected Ugandan adults receiving antiretroviral treatment and survival of their HIV-uninfected children: a prospective cohort study. | 2008 | Journal article | | | ✓ | | |

## Chronological reference list: Assessments included in the CBPHC review

| Reference | Year | Reference type | Type of assessment carried out | | | | |
|---|---|---|---|---|---|---|---|
| | | | | Child health | | | |
| | | | Mater-nal health | Neo-natal health | Health of children 1-59m | Health of 0-59-m-old children | Equity effect |
| Lancet, 371(9614), 752-759. doi:10.1016/S0140-6736(08)60345-1 | | | | | | | |
| Mullany, L. C., Katz, J., Li, Y. M., Khatry, S. K., LeClerq, S. C., Darmstadt, G. L., & Tielsch, J. M. (2008). Breast-feeding patterns, time to initiation, and mortality risk among newborns in southern Nepal. J Nutr, 138(3), 599-603. | 2008 | Journal article | | ✓ | | | |
| Newlands, D., Yugbare-Belemsaga, D., Ternent, L., Hounton, S., & Chapman, G. (2008). Assessing the costs and cost-effectiveness of a skilled care initiative in rural Burkina Faso. Trop Med Int Health, 13 Suppl 1, 61-67. doi:10.1111/j.1365-3156.2008.02088.x | 2008 | Journal article | ✓ | | | | |
| Omer, K., Mhatre, S., Ansari, N., Laucirica, J., & Andersson, N. (2008). Evidence-based training of frontline health workers for door-to-door health promotion: a pilot randomized controlled cluster trial with Lady Health Workers in Sindh Province, Pakistan. Patient Educ Couns, 72(2), 178-185. doi:10.1016/j.pec.2008.02.018 | 2008 | Journal article | ✓ | | | ✓ | |
| Rahman, A., Malik, A., Sikander, S., Roberts, C., & Creed, F. (2008). Cognitive behaviour therapy-based intervention by community health workers for mothers with depression and their infants in rural Pakistan: a cluster-randomised controlled trial. Lancet, 372(9642), 902-909. doi:10.1016/S0140-6736(08)61400-2 | 2008 | Journal article | ✓ | | | | |
| Ruel, M. T., Menon, P., Habicht, J. P., Loechl, C., Bergeron, G., Pelto, G., . . . Hankebo, B. (2008). Age-based preventive targeting of food assistance and behaviour change and communication for reduction of childhood undernutrition in Haiti: a cluster randomised trial. Lancet, 371(9612), 588-595. doi:10.1016/S0140-6736(08)60271-8 | 2008 | Journal article | | | | ✓ | |
| Save the Children/Bolivia (2008). Food security program: Assessment survey report. | 2008 | Report | | | | ✓ | |
| Sievers, A. C., Lewey, J., Musafiri, P., Franke, M. F., Bucyibaruta, B. J., Stulac, S. N., . . . Daily, J. P. (2008). Reduced paediatric hospitalizations for malaria and febrile illness patterns following implementation of community-based malaria control programme in rural Rwanda. Malar J, 7, 167. doi:10.1186/1475-2875-7-167 | 2008 | Journal article | | | ✓ | | |
| Sloan, N. L., Ahmed, S., Mitra, S. N., Choudhury, N., Chowdhury, M., Rob, U., & Winikoff, B. (2008). Community-based kangaroo mother care to prevent neonatal and infant mortality: a randomized, controlled cluster trial. Pediatrics, 121(5), e1047-1059. doi:10.1542/peds.2007-0076 | 2008 | Journal article | | ✓ | | | |
| Supplementation with Multiple Micronutrients Intervention Trial Study, G., Shankar, A. H., Jahari, A. B., Sebayang, S. K., Aditiawarman, Apriatni, M., . . . Sofia, G. (2008). Effect of maternal multiple micronutrient supplementation on fetal loss and infant death in Indonesia: a double-blind cluster-randomised trial. Lancet, 371(9608), 215-227. doi:10.1016/S0140-6736(08)60133-6 | 2008 | Journal article | | | | ✓ | |

## Chronological reference list: Assessments included in the CBPHC review

| Reference | Year | Reference type | Type of assessment carried out | | | | |
|---|---|---|---|---|---|---|---|
| | | | | Child health | | | |
| | | | Mater-nal health | Neo-natal health | Health of children 1-59m | Health of 0-59-m-old children | Equity effect |
| Tielsch, J. M., Rahmathullah, L., Katz, J., Thulasiraj, R. D., Coles, C., Sheeladevi, S., & Prakash, K. (2008). Maternal night blindness during pregnancy is associated with low birthweight, morbidity, and poor growth in South India. J Nutr, 138(4), 787-792. | 2008 | Journal article | | ✓ | | | |
| Tiono, A. B., Kabore, Y., Traore, A., Convelbo, N., Pagnoni, F., & Sirima, S. B. (2008). Implementation of Home based management of malaria in children reduces the work load for peripheral health facilities in a rural district of Burkina Faso. Malar J, 7, 201. doi:10.1186/1475-2875-7-201 | 2008 | Journal article | | | | ✓ | ✓ |
| World Vision/India (2008). Pragati Child Survival Project, Uttar Pradesh, India: Final Evaluation. | 2008 | Report | | | | ✓ | |
| Wuehler, S. E., Sempertegui, F., & Brown, K. H. (2008). Dose-response trial of prophylactic zinc supplements, with or without copper, in young Ecuadorian children at risk of zinc deficiency. Am J Clin Nutr, 87(3), 723-733. | 2008 | Journal article | | | | ✓ | |
| Zeba, A. N., Sorgho, H., Rouamba, N., Zongo, I., Rouamba, J., Guiguemde, R. T., . . . Ouedraogo, J. B. (2008). Major reduction of malaria morbidity with combined vitamin A and zinc supplementation in young children in Burkina Faso: a randomized double blind trial. Nutr J, 7, 7. doi:10.1186/1475-2891-7-7 | 2008 | Journal article | | | | ✓ | |
| Aquino, R., de Oliveira, N. F., & Barreto, M. L. (2009). Impact of the family health program on infant mortality in Brazilian municipalities. Am J Public Health, 99(1), 87-93. doi:10.2105/AJPH.2007.127480 | 2009 | Journal article | | | | ✓ | ✓ |
| Aracena, M., Krause, M., Perez, C., Mendez, M. J., Salvatierra, L., Soto, M., . . . Altimir, C. (2009). A cost-effectiveness evaluation of a home visit program for adolescent mothers. J Health Psychol, 14(7), 878-887. doi:10.1177/1359105309340988 | 2009 | Journal article | ✓ | | | | |
| Arifeen, S. E., Hoque, D. M., Akter, T., Rahman, M., Hoque, M. E., Begum, K., . . . Black, R. E. (2009). Effect of the Integrated Management of Childhood Illness strategy on childhood mortality and nutrition in a rural area in Bangladesh: a cluster randomised trial. Lancet, 374(9687), 393-403. doi:10.1016/S0140-6736(09)60828-X | 2009 | Journal article | | | ✓ | | ✓ |
| Bachmann, M. O. (2009). Cost effectiveness of community-based therapeutic care for children with severe acute malnutrition in Zambia: decision tree model. Cost Eff Resour Alloc, 7, 2. doi:10.1186/1478-7547-7-2 | 2009 | Journal article | | | | ✓ | |
| Baqui, A. H., Ahmed, S., El Arifeen, S., Darmstadt, G. L., Rosecrans, A. M., Mannan, I., . . . Projahnmo 1 Study, G. (2009). Effect of timing of first postnatal care home visit on neonatal mortality in Bangladesh: a observational cohort study. BMJ, 339, b2826. doi:10.1136/bmj.b2826 | 2009 | Journal article | | | | ✓ | |
| Baqui, A. H., Arifeen, S. E., Williams, E. K., Ahmed, S., Mannan, I., Rahman, S. M., . . . Darmstadt, G. L. (2009). Effectiveness of home-based management of newborn infections by community health workers in | 2009 | Journal article | | ✓ | | | |

## Chronological reference list: Assessments included in the CBPHC review

| Reference | Year | Reference type | Type of assessment carried out | | | | |
|---|---|---|---|---|---|---|---|
| | | | | Child health | | | |
| | | | Mater-nal health | Neo-natal health | Health of children 1-59m | Health of 0-59-m-old children | Equity effect |
| rural Bangladesh. Pediatr Infect Dis J, 28(4), 304-310. doi:10.1097/INF.0b013e31819069e8 | | | | | | | |
| Bhutta, Z. A., Rizvi, A., Raza, F., Hotwani, S., Zaidi, S., Moazzam Hossain, S., . . . Bhutta, S. (2009). A comparative evaluation of multiple micronutrient and iron-folic acid supplementation during pregnancy in Pakistan: impact on pregnancy outcomes. Food Nutr Bull, 30(4 Suppl), S496-505. doi:http://dx.doi.org/10.1177/15648265090304s404 | 2009 | Journal article | ✓ | | | ✓ | |
| Bisimwa, G., Mambo, T., Mitangala, P., Schirvel, C., Porignon, D., Dramaix, M., & Donnen, P. (2009). Nutritional monitoring of preschool-age children by community volunteers during armed conflict in the Democratic Republic of the Congo. Food Nutr Bull, 30(2), 120-127. doi:http://dx.doi.org/10.1177/156482650903000203 | 2009 | Journal article | | | | ✓ | |
| Christian, P., Stewart, C. P., LeClerq, S. C., Wu, L., Katz, J., West, K. P., Jr., & Khatry, S. K. (2009). Antenatal and postnatal iron supplementation and childhood mortality in rural Nepal: a prospective follow-up in a randomized, controlled community trial. Am J Epidemiol, 170(9), 1127-1136. doi:10.1093/aje/kwp253 | 2009 | Journal article | | | | ✓ | |
| Cisse, B., Cairns, M., Faye, E., O, N. D., Faye, B., Cames, C., . . . Milligan, P. (2009). Randomized trial of piperaquine with sulfadoxine-pyrimethamine or dihydroartemisinin for malaria intermittent preventive treatment in children. PLoS One, 4(9), e7164. doi:10.1371/journal.pone.0007164 | 2009 | Journal article | | | ✓ | | |
| Dongre, A. R., Deshmukh, P. R., & Garg, B. S. (2009). A community based approach to improve health care seeking for newborn danger signs in rural Wardha, India. Indian J Pediatr, 76(1), 45-50. doi:10.1007/s12098-009-0028-y | 2009 | Journal article | | ✓ | | | |
| Fernald, L. C., Gertler, P. J., & Neufeld, L. M. (2009). 10-year effect of Oportunidades, Mexico's conditional cash transfer programme, on child growth, cognition, language, and behaviour: a longitudinal follow-up study. Lancet, 374(9706), 1997-2005. doi:10.1016/S0140-6736(09)61676-7 | 2009 | Journal article | | | | ✓ | |
| Geissbuhler, Y., Kannady, K., Chaki, P. P., Emidi, B., Govella, N. J., Mayagaya, V., . . . Killeen, G. F. (2009). Microbial larvicide application by a large-scale, community-based program reduces malaria infection prevalence in urban Dar es Salaam, Tanzania. PLoS One, 4(3), e5107. doi:10.1371/journal.pone.0005107 | 2009 | Journal article | | | | ✓ | |
| Hawkes, M., Katsuva, J. P., & Masumbuko, C. K. (2009). Use and limitations of malaria rapid diagnostic testing by community health workers in war-torn Democratic Republic of Congo. Malar J, 8, 308. doi:10.1186/1475-2875-8-308 | 2009 | Journal article | | | | ✓ | |
| Khan, M. A., & Ahmed, S. M. (2009). The "Birthing Hut" facilities of MANOSHI: A Two-Part Paper, Exploring the Inception and Post-Inception Phases of Urban Delivery Centres of Dhaka. | 2009 | Report | ✓ | | | | |

| Chronological reference list: Assessments included in the CBPHC review | | | | | | | |
|---|---|---|---|---|---|---|---|
| | | | Type of assessment carried out | | | | |
| | | | | Child health | | | |
| Reference | Year | Reference type | Maternal health | Neonatal health | Health of children 1-59m | Health of 0-59-m-old children | Equity effect |
| Msyamboza, K. P., Savage, E. J., Kazembe, P. N., Gies, S., Kalanda, G., D'Alessandro, U., & Brabin, B. J. (2009). Community-based distribution of sulfadoxine-pyrimethamine for intermittent preventive treatment of malaria during pregnancy improved coverage but reduced antenatal attendance in southern Malawi. Trop Med Int Health, 14(2), 183-189. doi:10.1111/j.1365-3156.2008.02197.x | 2009 | Journal article | ✓ | | | | |
| Naheed, A., Walker Fischer, C. L., Mondal, D., Ahmed, S., Arifeen, S. E., Yunus, M., . . . Baqui, A. H. (2009). Zinc therapy for diarrhoea improves growth among Bangladeshi infants 6 to 11 months of age. J Pediatr Gastroenterol Nutr, 48(1), 89-93. doi:10.1097/MPG.0b013e31817f0182 | 2009 | Journal article | | | ✓ | | |
| Ndiaye, M., Siekmans, K., Haddad, S., & Receveur, O. (2009). Impact of a positive deviance approach to improve the effectiveness of an iron-supplementation program to control nutritional anemia among rural Senegalese pregnant women. Food Nutr Bull, 30(2), 128-136. doi:http://dx.doi.org/10.1177/156482650903000204 | 2009 | Journal article | ✓ | | | | |
| Otten, M., Aregawi, M., Were, W., Karema, C., Medin, A., Bekele, W., . . . Grabowsky, M. (2009). Initial evidence of reduction of malaria cases and deaths in Rwanda and Ethiopia due to rapid scale-up of malaria prevention and treatment. Malar J, 8, 14. doi:10.1186/1475-2875-8-14 | 2009 | Journal article | | | ✓ | | |
| Perez, F., Ba, H., Dastagire, S. G., & Altmann, M. (2009). The role of community health workers in improving child health programmes in Mali. BMC Int Health Hum Rights, 9, 28. doi:10.1186/1472-698X-9-28 | 2009 | Journal article | | | | ✓ | |
| Porco, T. C., Gebre, T., Ayele, B., House, J., Keenan, J., Zhou, Z., . . . Lietman, T. M. (2009). Effect of mass distribution of azithromycin for trachoma control on overall mortality in Ethiopian children: a randomized trial. JAMA, 302(9), 962-968. doi:10.1001/jama.2009.1266 | 2009 | Journal article | | | | ✓ | |
| Purdin, S., Khan, T., & Saucier, R. (2009). Reducing maternal mortality among Afghan refugees in Pakistan. Int J Gynaecol Obstet, 105(1), 82-85. doi:10.1016/j.ijgo.2008.12.021 | 2009 | Journal article | ✓ | | | | |
| Rutherford, M. E., Dockerty, J. D., Jasseh, M., Howie, S. R., Herbison, P., Jeffries, D. J., . . . Hill, P. C. (2009). Preventive measures in infancy to reduce under-five mortality: a case-control study in The Gambia. Trop Med Int Health, 14(2), 149-155. doi:10.1111/j.1365-3156.2008.02204.x | 2009 | Journal article | | | | ✓ | |
| Schumann, K., Longfils, P., Monchy, D., von Xylander, S., Weinheimer, H., & Solomons, N. W. (2009). Efficacy and safety of twice-weekly administration of three RDAs of iron and folic acid with and without complement of 14 essential micronutrients at one or two RDAs: a placebo-controlled intervention trial in anemic Cambodian infants 6 to 24 months of age. Eur | 2009 | Journal article | | | | ✓ | |

| Chronological reference list: Assessments included in the CBPHC review | | | | | | | |
|---|---|---|---|---|---|---|---|
| | | | Type of assessment carried out | | | | |
| | | | | Child health | | | |
| Reference | Year | Reference type | Mater-nal health | Neo-natal health | Health of children 1-59m | Health of 0-59-m-old children | Equity effect |
| J Clin Nutr, 63(3), 355-368. doi:10.1038/sj.ejcn.1602930 | | | | | | | |
| Skinner, J., & Rathavy, T. (2009). Design and evaluation of a community participatory, birth preparedness project in Cambodia. Midwifery, 25(6), 738-743. doi:10.1016/j.midw.2008.01.006 | 2009 | Journal article | ✓ | | | | |
| Stewart, C. P., Christian, P., LeClerq, S. C., West, K. P., Jr., & Khatry, S. K. (2009). Antenatal supplementation with folic acid + iron + zinc improves linear growth and reduces peripheral adiposity in school-age children in rural Nepal. Am J Clin Nutr, 90(1), 132-140. doi:10.3945/ajcn.2008.27368 | 2009 | Journal article | | | | ✓ | |
| Thang, N. D., Erhart, A., Hung le, X., Thuan le, K., Xa, N. X., Thanh, N. N., . . . D'Alessandro, U. (2009). Rapid decrease of malaria morbidity following the introduction of community-based monitoring in a rural area of central Vietnam. Malar J, 8, 3. doi:10.1186/1475-2875-8-3 | 2009 | Journal article | | | ✓ | | |
| Thompson, M. E., & Harutyunyan, T. L. (2009). Impact of a community-based integrated management of childhood illnesses (IMCI) programme in Gegharkunik, Armenia. Health Policy Plan, 24(2), 101-107. doi:10.1093/heapol/czn048 | 2009 | Journal article | | | | ✓ | |
| Ahluwalia, I. B., Robinson, D., Vallely, L., Gieseker, K. E., & Kabakama, A. (2010). Sustainability of community-capacity to promote safer motherhood in northwestern Tanzania: what remains? Glob Health Promot, 17(1), 39-49. doi:10.1177/1757975909356627 | 2010 | Journal article | ✓ | | | | |
| Azad, K., Barnett, S., Banerjee, B., Shaha, S., Khan, K., Rego, A. R., . . . Costello, A. (2010). Effect of scaling up women's groups on birth outcomes in three rural districts in Bangladesh: a cluster-randomised controlled trial. Lancet, 375(9721), 1193-1202. doi:10.1016/S0140-6736(10)60142-0 | 2010 | Journal article | ✓ | | | ✓ | |
| Darmstadt, G. L., Choi, Y., Arifeen, S. E., Bari, S., Rahman, S. M., Mannan, I., . . . Bangladesh Projahnmo-2 Mirzapur Study, G. (2010). Evaluation of a cluster-randomized controlled trial of a package of community-based maternal and newborn interventions in Mirzapur, Bangladesh. PLoS One, 5(3), e9696. doi:10.1371/journal.pone.0009696 | 2010 | Journal article | ✓ | | | | |
| Eriksen, J., Mujinja, P., Warsame, M., Nsimba, S., Kouyate, B., Gustafsson, L. L., . . . Tomson, G. (2010). Effectiveness of a community intervention on malaria in rural Tanzania - a randomised controlled trial. Afr Health Sci, 10(4), 332-340. | 2010 | Journal article | | | | ✓ | |
| Ghimire, M., Pradhan, Y. V., & Maskey, M. K. (2010). Community-based interventions for diarrhoeal diseases and acute respiratory infections in Nepal. Bull World Health Organ, 88(3), 216-221. doi:10.2471/BLT.09.065649 | 2010 | Journal article | | | | ✓ | |
| Hodgins, S., McPherson, R., Suvedi, B. K., Shrestha, R. B., Silwal, R. C., Ban, B., . . . Baqui, A. H. (2010). Testing a scalable community-based approach to improve | 2010 | Journal article | ✓ | | | ✓ | |

| Chronological reference list: Assessments included in the CBPHC review | | | | | | | |
|---|---|---|---|---|---|---|---|
| | | | Type of assessment carried out | | | | |
| | | | | Child health | | | |
| Reference | Year | Reference type | Mater-nal health | Neo-natal health | Health of children 1-59m | Health of 0-59-m-old children | Equity effect |
| maternal and neonatal health in rural Nepal. J Perinatol, 30(6), 388-395. doi:10.1038/jp.2009.181 | | | | | | | |
| Lemma, H., Byass, P., Desta, A., Bosman, A., Costanzo, G., Toma, L., . . . Barnabas, G. A. (2010). Deploying artemether-lumefantrine with rapid testing in Ethiopian communities: impact on malaria morbidity, mortality and healthcare resources. Trop Med Int Health, 15(2), 241-250. doi:10.1111/j.1365-3156.2009.02447.x | 2010 | Journal article | | | ✓ | | |
| Lim, S. S., Dandona, L., Hoisington, J. A., James, S. L., Hogan, M. C., & Gakidou, E. (2010). India's Janani Suraksha Yojana, a conditional cash transfer programme to increase births in health facilities: an impact evaluation. Lancet, 375(9730), 2009-2023. doi:10.1016/S0140-6736(10)60744-1 | 2010 | Journal article | ✓ | | | | |
| Lugada, E., Levin, J., Abang, B., Mermin, J., Mugalanzi, E., Namara, G., . . . Bunnell, R. (2010). Comparison of home and clinic-based HIV testing among household members of persons taking antiretroviral therapy in Uganda: results from a randomized trial. J Acquir Immune Defic Syndr, 55(2), 245-252. doi:10.1097/QAI.0b013e3181e9e069 | 2010 | Journal article | | | ✓ | | |
| Lundeen, E., Schueth, T., Toktobaev, N., Zlotkin, S., Hyder, S. M., & Houser, R. (2010). Daily use of Sprinkles micronutrient powder for 2 months reduces anemia among children 6 to 36 months of age in the Kyrgyz Republic: a cluster-randomized trial. Food Nutr Bull, 31(3), 446-460. doi:http://dx.doi.org/10.1177/156482651003100307 | 2010 | Journal article | | | | ✓ | |
| Mazumder, S., Taneja, S., Bhandari, N., Dube, B., Agarwal, R. C., Mahalanabis, D., . . . Black, R. E. (2010). Effectiveness of zinc supplementation plus oral rehydration salts for diarrhoea in infants aged less than 6 months in Haryana state, India. Bull World Health Organ, 88(10), 754-760. doi:10.2471/BLT.10.075986 | 2010 | Journal article | | | ✓ | | |
| Midhet, F., & Becker, S. (2010). Impact of community-based interventions on maternal and neonatal health indicators: Results from a community randomized trial in rural Balochistan, Pakistan. Reprod Health, 7, 30. doi:10.1186/1742-4755-7-30 | 2010 | Journal article | ✓ | | | | |
| Mullany, L. C., Lee, T. J., Yone, L., Lee, C. I., Teela, K. C., Paw, P., . . . Beyrer, C. (2010). Impact of community-based maternal health workers on coverage of essential maternal health interventions among internally displaced communities in eastern Burma: the MOM project. PLoS Med, 7(8), e1000317. doi:10.1371/journal.pmed.1000317 | 2010 | Journal article | ✓ | | | | |
| Mushi, D., Mpembeni, R., & Jahn, A. (2010). Effectiveness of community based Safe Motherhood promoters in improving the utilization of obstetric care. The case of Mtwara Rural District in Tanzania. BMC Pregnancy Childbirth, 10, 14. doi:10.1186/1471-2393-10-14 | 2010 | Journal article | ✓ | | | | |

| Chronological reference list: Assessments included in the CBPHC review | | | | | | | |
|---|---|---|---|---|---|---|---|
| | | | Type of assessment carried out | | | | |
| | | | | Child health | | | |
| Reference | Year | Reference type | Mater-nal health | Neo-natal health | Health of children 1-59m | Health of 0-59-m-old children | Equity effect |
| Opryszko, M. C., Majeed, S. W., Hansen, P. M., Myers, J. A., Baba, D., Thompson, R. E., & Burnham, G. (2010). Water and hygiene interventions to reduce diarrhoea in rural Afghanistan: a randomized controlled study. J Water Health, 8(4), 687-702. doi:10.2166/wh.2010.121 | 2010 | Journal article | | | | ✓ | |
| Rabbani, G. H., Larson, C. P., Islam, R., Saha, U. R., & Kabir, A. (2010). Green banana-supplemented diet in the home management of acute and prolonged diarrhoea in children: a community-based trial in rural Bangladesh. Trop Med Int Health, 15(10), 1132-1139. doi:10.1111/j.1365-3156.2010.02608.x | 2010 | Journal article | | | | ✓ | |
| Rajbhandari, S., Hodgins, S., Sanghvi, H., McPherson, R., Pradhan, Y. V., Baqui, A. H., & Misoprostol Study, G. (2010). Expanding uterotonic protection following childbirth through community-based distribution of misoprostol: operations research study in Nepal. Int J Gynaecol Obstet, 108(3), 282-288. doi:10.1016/j.ijgo.2009.11.006 | 2010 | Journal article | ✓ | | | | |
| Rath, S., Nair, N., Tripathy, P. K., Barnett, S., Rath, S., Mahapatra, R., . . . Prost, A. (2010). Explaining the impact of a women's group led community mobilisation intervention on maternal and newborn health outcomes: the Ekjut trial process evaluation. BMC Int Health Hum Rights, 10, 25. doi:10.1186/1472-698X-10-25 | 2010 | Journal article | ✓ | | | | |
| Ryman, T., Macauley, R., Nshimirimana, D., Taylor, P., Shimp, L., & Wilkins, K. (2010). Reaching every district (RED) approach to strengthen routine immunization services: evaluation in the African region, 2005. J Public Health (Oxf), 32(1), 18-25. doi:10.1093/pubmed/fdp048 | 2010 | Journal article | | | | ✓ | |
| Sanghvi, H., Ansari, N., Prata, N. J., Gibson, H., Ehsan, A. T., & Smith, J. M. (2010). Prevention of postpartum hemorrhage at home birth in Afghanistan. Int J Gynaecol Obstet, 108(3), 276-281. doi:10.1016/j.ijgo.2009.12.003 | 2010 | Journal article | ✓ | | | | |
| Tripathy, P., Nair, N., Barnett, S., Mahapatra, R., Borghi, J., Rath, S., . . . Costello, A. (2010). Effect of a participatory intervention with women's groups on birth outcomes and maternal depression in Jharkhand and Orissa, India: a cluster-randomised controlled trial. Lancet, 375(9721), 1182-1192. doi:10.1016/S0140-6736(09)62042-0 | 2010 | Journal article | ✓ | | | ✓ | |
| Yeboah-Antwi, K., Pilingana, P., Macleod, W. B., Semrau, K., Siazeele, K., Kalesha, P., . . . Hamer, D. H. (2010). Community case management of fever due to malaria and pneumonia in children under five in Zambia: a cluster randomized controlled trial. PLoS Med, 7(9), e1000340. doi:10.1371/journal.pmed.1000340 | 2010 | Journal article | | | ✓ | | |
| Aaby, P., Roth, A., Ravn, H., Napirna, B. M., Rodrigues, A., Lisse, I. M., . . . Benn, C. S. (2011). Randomized trial of BCG vaccination at birth to low-birth-weight children: beneficial nonspecific effects in the neonatal | 2011 | Journal article | | | | ✓ | |

| Chronological reference list: Assessments included in the CBPHC review | | | | | | | |
|---|---|---|---|---|---|---|---|
| | | | Type of assessment carried out | | | | |
| | | | | Child health | | | |
| Reference | Year | Reference type | Mater-nal health | Neo-natal health | Health of children 1-59m | Health of 0-59-m-old children | Equity effect |
| period? J Infect Dis, 204(2), 245-252. doi:10.1093/infdis/jir240 | | | | | | | |
| Agha, S. (2011). Impact of a maternal health voucher scheme on institutional delivery among low income women in Pakistan. Reprod Health, 8, 10. doi:10.1186/1742-4755-8-10 | 2011 | Journal article | ✓ | | | | |
| Alam, D. S., Yunus, M., El Arifeen, S., Chowdury, H. R., Larson, C. P., Sack, D. A., . . . Black, R. E. (2011). Zinc treatment for 5 or 10 days is equally efficacious in preventing diarrhea in the subsequent 3 months among Bangladeshi children. J Nutr, 141(2), 312-315. doi:10.3945/jn.110.120857 | 2011 | Journal article | | | | ✓ | |
| Bari, A., Sadruddin, S., Khan, A., Khan, I., Khan, A., Lehri, I. A., . . . Qazi, S. A. (2011). Community case management of severe pneumonia with oral amoxicillin in children aged 2-59 months in Haripur district, Pakistan: a cluster randomised trial. Lancet, 378(9805), 1796-1803. doi:10.1016/S0140-6736(11)61140-9 | 2011 | Journal article | | | ✓ | | |
| Bhutta, Z. A., Soofi, S., Cousens, S., Mohammad, S., Memon, Z. A., Ali, I., . . . Martines, J. (2011). Improvement of perinatal and newborn care in rural Pakistan through community-based strategies: a cluster-randomised effectiveness trial. Lancet, 377(9763), 403-412. doi:10.1016/S0140-6736(10)62274-X | 2011 | Journal article | ✓ | ✓ | | | |
| Bojang, K. A., Akor, F., Conteh, L., Webb, E., Bittaye, O., Conway, D. J., . . . Greenwood, B. (2011). Two strategies for the delivery of IPTc in an area of seasonal malaria transmission in the Gambia: a randomised controlled trial. PLoS Med, 8(2), e1000409. doi:10.1371/journal.pmed.1000409 | 2011 | Journal article | | | ✓ | | ✓ |
| Brenner, J. L., Kabakyenga, J., Kyomuhangi, T., Wotton, K. A., Pim, C., Ntaro, M., . . . Singhal, N. (2011). Can volunteer community health workers decrease child morbidity and mortality in southwestern Uganda? An impact evaluation. PLoS One, 6(12), e27997. doi:10.1371/journal.pone.0027997 | 2011 | Journal article | | | | ✓ | |
| Care/Nepal. (2011). Community Responsive Antenatal, Delivery, and Life Essential (CRADLE) Support Program (MANASHI): Final Evaluation Report, Doti and Kailali districts of Far Western Region of Nepal. | 2011 | Report | | ✓ | | | |
| Concern Worldwide/Rwanda (2011). Final evaluation of the Kabeho Mwana expanded impact child survival program. | 2011 | Report | | | | ✓ | |
| Dongre, A. R., Deshmukh, P. R., & Garg, B. S. (2011). Community-led initiative for control of anemia among children 6 to 35 months of age and unmarried adolescent girls in rural Wardha, India. Food Nutr Bull, 32(4), 315-323. doi:http://dx.doi.org/10.1177/156482651103200402 | 2011 | Journal article | | | | ✓ | |
| du Preez, M., Conroy, R. M., Ligondo, S., Hennessy, J., Elmore-Meegan, M., Soita, A., & McGuigan, K. G. (2011). Randomized intervention study of solar | 2011 | Journal article | | | ✓ | | |

## Chronological reference list: Assessments included in the CBPHC review

| Reference | Year | Reference type | Type of assessment carried out | | | | |
|---|---|---|---|---|---|---|---|
| | | | | Child health | | | |
| | | | Mater-nal health | Neo-natal health | Health of children 1-59m | Health of 0-59-m-old children | Equity effect |
| disinfection of drinking water in the prevention of dysentery in Kenyan children aged under 5 years. Environ Sci Technol, 45(21), 9315-9323. doi:10.1021/es2018835 | | | | | | | |
| Dynes, M., Rahman, A., Beck, D., Moran, A., Rahman, A., Pervin, J., . . . Sibley, L. (2011). Home-based life saving skills in Matlab, Bangladesh: a process evaluation of a community-based maternal child health programme. Midwifery, 27(1), 15-22. doi:10.1016/j.midw.2009.07.009 | 2011 | Journal article | ✓ | | | | |
| Escamilla, V., Wagner, B., Yunus, M., Streatfield, P. K., van Geen, A., & Emch, M. (2011). Effect of deep tube well use on childhood diarrhoea in Bangladesh. Bull World Health Organ, 89(7), 521-527. doi:10.2471/BLT.10.085530 | 2011 | Journal article | | | | ✓ | |
| Goal/Ethiopia. (2011). Sidama Child Survival Project/Awassa Zuriya and Boricha Waredas (districts), SidamanZone, SNNPR, Ethiopia: Final evaluation project. | 2011 | Report | | | | ✓ | |
| Rescue International/Niger (2011). Health start child survival program: Child survival and health grants programs. | 2011 | Report | | | | ✓ | |
| Khanal, S., Sharma, J., Gc, V. S., Dawson, P., Houston, R., Khadka, N., & Yengden, B. (2011). Community health workers can identify and manage possible infections in neonates and young infants: MINI--a model from Nepal. J Health Popul Nutr, 29(3), 255-264. doi:http://dx.doi.org/10.3329/jhpn.v29i3.7873 | 2011 | Journal article | | ✓ | | | |
| Kounnavong, S., Sunahara, T., Mascie-Taylor, C. G., Hashizume, M., Okumura, J., Moji, K., . . . Yamamoto, T. (2011). Effect of daily versus weekly home fortification with multiple micronutrient powder on haemoglobin concentration of young children in a rural area, Lao People's Democratic Republic: a randomised trial. Nutr J, 10, 129. doi:10.1186/1475-2891-10-129 | 2011 | Journal article | | | | ✓ | |
| McGuigan, K. G., Samaiyar, P., du Preez, M., & Conroy, R. M. (2011). High compliance randomized controlled field trial of solar disinfection of drinking water and its impact on childhood diarrhea in rural Cambodia. Environ Sci Technol, 45(18), 7862-7867. doi:10.1021/es201313x | 2011 | Journal article | | | ✓ | | |
| Mehta, S., Mugusi, F. M., Bosch, R. J., Aboud, S., Chatterjee, A., Finkelstein, J. L., . . . Fawzi, W. W. (2011). A randomized trial of multivitamin supplementation in children with tuberculosis in Tanzania. Nutr J, 10, 120. doi:10.1186/1475-2891-10-120 | 2011 | Journal article | | | | ✓ | |
| Mobeen, N., Durocher, J., Zuberi, N., Jahan, N., Blum, J., Wasim, S., . . . Hatcher, J. (2011). Administration of misoprostol by trained traditional birth attendants to prevent postpartum haemorrhage in homebirths in Pakistan: a randomised placebo-controlled trial. BJOG, 118(3), 353-361. doi:10.1111/j.1471-0528.2010.02807.x | 2011 | Journal article | ✓ | | | | |

| Chronological reference list: Assessments included in the CBPHC review | | | | | | | |
|---|---|---|---|---|---|---|---|
| | | | Type of assessment carried out | | | | |
| | | | | Child health | | | |
| Reference | Year | Reference type | Mater-nal health | Neo-natal health | Health of children 1-59m | Health of 0-59-m-old children | Equity effect |
| Mubi, M., Janson, A., Warsame, M., Martensson, A., Kallander, K., Petzold, M. G., . . . Bjorkman, A. (2011). Malaria rapid testing by community health workers is effective and safe for targeting malaria treatment: randomised cross-over trial in Tanzania. PLoS One, 6(7), e19753. doi:10.1371/journal.pone.0019753 | 2011 | Journal article | | | ✓ | | |
| Mukanga, D., Babirye, R., Peterson, S., Pariyo, G. W., Ojiambo, G., Tibenderana, J. K., . . . Kallander, K. (2011). Can lay community health workers be trained to use diagnostics to distinguish and treat malaria and pneumonia in children? Lessons from rural Uganda. Trop Med Int Health, 16(10), 1234-1242. doi:10.1111/j.1365-3156.2011.02831.x | 2011 | Journal article | | | ✓ | | |
| Nankabirwa, V., Tylleskar, T., Nankunda, J., Engebretsen, I. M., Sommerfelt, H., Tumwine, J. K., & Consortium, P. E. R. (2011). Malaria parasitaemia among infants and its association with breastfeeding peer counselling and vitamin A supplementation: a secondary analysis of a cluster randomized trial. PLoS One, 6(7), e21862. doi:10.1371/journal.pone.0021862 | 2011 | Journal article | | | | ✓ | |
| Ngasala, B. E., Malmberg, M., Carlsson, A. M., Ferreira, P. E., Petzold, M. G., Blessborn, D., . . . Martensson, A. (2011). Effectiveness of artemether-lumefantrine provided by community health workers in under-five children with uncomplicated malaria in rural Tanzania: an open label prospective study. Malar J, 10, 64. doi:10.1186/1475-2875-10-64 | 2011 | Journal article | | | ✓ | | |
| Okeibunor, J. C., Orji, B. C., Brieger, W., Ishola, G., Otolorin, E., Rawlins, B., . . . Fink, G. (2011). Preventing malaria in pregnancy through community-directed interventions: evidence from Akwa Ibom State, Nigeria. Malar J, 10, 227. doi:10.1186/1475-2875-10-227 | 2011 | Journal article | ✓ | | | | |
| Patouillard, E., Conteh, L., Webster, J., Kweku, M., Chandramohan, D., & Greenwood, B. (2011). Coverage, adherence and costs of intermittent preventive treatment of malaria in children employing different delivery strategies in Jasikan, Ghana. PLoS One, 6(11), e24871. doi:10.1371/journal.pone.0024871 | 2011 | Journal article | | | ✓ | | |
| Plan/Nepal. (2011). Local innovation for better outcomes for neonates (LIBON) project: Plan Nepal Child Survival Project XXVII, Sunsari, Parsa, and Bara districts in Nepal. | 2011 | Report | | | | ✓ | |
| Qureshi, A. M., Oche, O. M., Sadiq, U. A., & Kabiru, S. (2011). Using community volunteers to promote exclusive breastfeeding in Sokoto State, Nigeria. Pan Afr Med J, 10, 8. doi:http://dx.doi.org/10.4314/pamj.v10i0.72215 | 2011 | Journal article | | ✓ | | | |
| Ryman, T. K., Trakroo, A., Wallace, A., Gupta, S. K., Wilkins, K., Mehta, P., & Dietz, V. (2011). Implementation and evaluation of the Reaching Every District (RED) strategy in Assam, India, 2005-2008. Vaccine, 29(14), 2555-2560. doi:10.1016/j.vaccine.2011.01.061 | 2011 | Journal article | | | | ✓ | |

| Chronological reference list: Assessments included in the CBPHC review | | | | | | | | |
|---|---|---|---|---|---|---|---|---|
| | | | Type of assessment carried out | | | | | |
| | | | | Child health | | | | |
| Reference | Year | Reference type | Mater-nal health | Neo-natal health | Health of children 1-59m | Health of 0-59-m-old children | Equity effect |
| Sesay, S., Milligan, P., Touray, E., Sowe, M., Webb, E. L., Greenwood, B. M., & Bojang, K. A. (2011). A trial of intermittent preventive treatment and home-based management of malaria in a rural area of The Gambia. Malar J, 10, 2. doi:10.1186/1475-2875-10-2 | 2011 | Journal article | | | ✓ | | |
| Sur, D., Manna, B., Niyogi, S. K., Ramamurthy, T., Palit, A., Nomoto, K., . . . Bhattacharya, S. K. (2011). Role of probiotic in preventing acute diarrhoea in children: a community-based, randomized, double-blind placebo-controlled field trial in an urban slum. Epidemiol Infect, 139(6), 919-926. doi:10.1017/S0950268810001780 | 2011 | Journal article | | | | ✓ | |
| Tine, R. C., Faye, B., Ndour, C. T., Ndiaye, J. L., Ndiaye, M., Bassene, C., . . . Gaye, O. (2011). Impact of combining intermittent preventive treatment with home management of malaria in children less than 10 years in a rural area of Senegal: a cluster randomized trial. Malar J, 10, 358. doi:10.1186/1475-2875-10-358 | 2011 | Journal article | | | ✓ | | |
| Turan, J. M., Tesfagiorghis, M., & Polan, M. L. (2011). Evaluation of a community intervention for promotion of safe motherhood in Eritrea. J Midwifery Womens Health, 56(1), 8-17. doi:10.1111/j.1542-2011.2010.00001.x | 2011 | Journal article | ✓ | | | | |
| Agrawal, P. K., Agrawal, S., Ahmed, S., Darmstadt, G. L., Williams, E. K., Rosen, H. E., . . . Baqui, A. H. (2012). Effect of knowledge of community health workers on essential newborn health care: a study from rural India. Health Policy Plan, 27(2), 115-126. doi:10.1093/heapol/czr018 | 2012 | Journal article | | ✓ | | | |
| Alehagen, S. A., Finnstrom, O., Hermansson, G. V., Somasundaram, K. V., Bangal, V. B., Patil, A., . . . Johansson, A. K. (2012). Nurse-based antenatal and child health care in rural India, implementation and effects - an Indian-Swedish collaboration. Rural Remote Health, 12, 2140. | 2012 | Journal article | ✓ | | | | |
| Arifeen, S. E., Mullany, L. C., Shah, R., Mannan, I., Rahman, S. M., Talukder, M. R., . . . Baqui, A. H. (2012). The effect of cord cleansing with chlorhexidine on neonatal mortality in rural Bangladesh: a community-based, cluster-randomised trial. Lancet, 379(9820), 1022-1028. doi:10.1016/S0140-6736(11)61848-5 | 2012 | Journal article | | ✓ | | | |
| Balaluka, G. B., Nabugobe, P. S., Mitangala, P. N., Cobohwa, N. B., Schirvel, C., Dramaix, M. W., & Donnen, P. (2012). Community volunteers can improve breastfeeding among children under six months of age in the Democratic Republic of Congo crisis. Int Breastfeed J, 7, 2. doi:10.1186/1746-4358-7-2 | 2012 | Journal article | | ✓ | | | |

| Chronological reference list: Assessments included in the CBPHC review | | | | | | | |
|---|---|---|---|---|---|---|---|
| | | | Type of assessment carried out | | | | |
| | | | | Child health | | | |
| Reference | Year | Reference type | Mater-nal health | Neo-natal health | Health of children 1-59m | Health of 0-59-m-old children | Equity effect |
| Bawah, A. A., Philip, J. F., Asuming, P., Walega, P., Wak, G., Schmitt, M., & Oduro, A. (2012). Can Community Health Services Offset the Effect of Poverty and Low Maternal Educational Attainment on Childhood Mortality? Evidence from the Navrongo Experiment in Northern Ghana. Paper presented at the Second Global Symposium on Health Systems Research, Beijing, China. | 2012 | Conference proceedings | | | | ✓ | |
| Bhandari, N., Mazumder, S., Taneja, S., Sommerfelt, H., Strand, T. A., & Group, I. E. S. (2012). Effect of implementation of Integrated Management of Neonatal and Childhood Illness (IMNCI) programme on neonatal and infant mortality: cluster randomised controlled trial. BMJ, 344, e1634. doi:10.1136/bmj.e1634 | 2012 | Journal article | | ✓ | | | |
| Bowen, A., Agboatwalla, M., Luby, S., Tobery, T., Ayers, T., & Hoekstra, R. M. (2012). Association between intensive handwashing promotion and child development in Karachi, Pakistan: a cluster randomized controlled trial. Arch Pediatr Adolesc Med, 166(11), 1037-1044. doi:10.1001/archpediatrics.2012.1181 | 2012 | Journal article | | | ✓ | | |
| Coffey, P. S., Sharma, J., Gargi, K. C., Neupane, D., Dawson, P., & Pradhan, Y. V. (2012). Feasibility and acceptability of gentamicin in the Uniject prefilled injection system for community-based treatment of possible neonatal sepsis: the experience of female community health volunteers in Nepal. J Perinatol, 32(12), 959-965. doi:10.1038/jp.2012.20 | 2012 | Journal article | | ✓ | | | |
| Catholic Relief Services/Nicaragua. (2012). Child Survival and Health Project: Final Evaluation. | 2012 | Report | | | | ✓ | |
| Deribew, A., Birhanu, Z., Sena, L., Dejene, T., Reda, A. A., Sudhakar, M., . . . Deribe, K. (2012). The effect of household heads training about the use of treated bed nets on the burden of malaria and anaemia in under-five children: a cluster randomized trial in Ethiopia. Malar J, 11, 8. doi:10.1186/1475-2875-11-8 | 2012 | Journal article | | | ✓ | | |
| Grimwood, A., Fatti, G., Mothibi, E., Malahlela, M., Shea, J., & Eley, B. (2012). Community adherence support improves programme retention in children on antiretroviral treatment: a multicentre cohort study in South Africa. J Int AIDS Soc, 15(2), 17381. doi:10.7448/IAS.15.2.17381 | 2012 | Journal article | | | | ✓ | |
| Hamer, D. H., Brooks, E. T., Semrau, K., Pilingana, P., MacLeod, W. B., Siazeele, K., . . . Yeboah-Antwi, K. (2012). Quality and safety of integrated community case management of malaria using rapid diagnostic tests and pneumonia by community health workers. Pathog Glob Health, 106(1), 32-39. doi:10.1179/1364859411Y.0000000042 | 2012 | Journal article | | | | ✓ | |
| Helen Keller International/Nepal (2012). Action against malnutrition through agriculture: Nepal Child Survival Project, Kailali and Baitadi districts, Far Western Region, Bajura expansion district. | 2012 | Report | | | | ✓ | |

| Chronological reference list: Assessments included in the CBPHC review | | | | | | | |
|---|---|---|---|---|---|---|---|
| | | | Type of assessment carried out | | | | |
| | | | | Child health | | | |
| Reference | Year | Reference type | Maternal health | Neonatal health | Health of children 1-59m | Health of 0-59-m-old children | Equity effect |
| Kalyango, J. N., Rutebemberwa, E., Alfven, T., Ssali, S., Peterson, S., & Karamagi, C. (2012). Performance of community health workers under integrated community case management of childhood illnesses in eastern Uganda. Malar J, 11, 282. doi:10.1186/1475-2875-11-282 | 2012 | Journal article | | | | ✓ | |
| Keating, J., Hutchinson, P., Miller, J. M., Bennett, A., Larsen, D. A., Hamainza, B., . . . Eisele, T. P. (2012). A quasi-experimental evaluation of an interpersonal communication intervention to increase insecticide-treated net use among children in Zambia. Malar J, 11, 313. doi:10.1186/1475-2875-11-313 | 2012 | Journal article | | | ✓ | | |
| Kema, K. M., Komwihangiro, J., & Kimaro, S. (2012). Integrated community based child survival, reproductive health and water and sanitation program in Mkuranga district, Tanzania: a replicable model of good practices in community based health care. Pan Afr Med J, 13 Suppl 1, 11. | 2012 | Journal article | ✓ | | | | |
| Kim, M. H., Ahmed, S., Buck, W. C., Preidis, G. A., Hosseinipour, M. C., Bhalakia, A., . . . Kline, M. W. (2012). The Tingathe programme: a pilot intervention using community health workers to create a continuum of care in the prevention of mother to child transmission of HIV (PMTCT) cascade of services in Malawi. J Int AIDS Soc, 15 Suppl 2, 17389. doi:10.7448/IAS.15.4.17389 | 2012 | Journal article | ✓ | | ✓ | | |
| Kisia, J., Nelima, F., Otieno, D. O., Kiilu, K., Emmanuel, W., Sohani, S., . . . Akhwale, W. (2012). Factors associated with utilization of community health workers in improving access to malaria treatment among children in Kenya. Malar J, 11, 248. doi:10.1186/1475-2875-11-248 | 2012 | Journal article | | | ✓ | | |
| Kumar, V., Kumar, A., Das, V., Srivastava, N. M., Baqui, A. H., Santosham, M., . . . Saksham Study, G. (2012). Community-driven impact of a newborn-focused behavioral intervention on maternal health in Shivgarh, India. Int J Gynaecol Obstet, 117(1), 48-55. doi:10.1016/j.ijgo.2011.10.031 | 2012 | Journal article | ✓ | | | | |
| Lori, J. R., Amable, E. E., Mertz, S. G., & Moriarty, K. (2012). Behavior change following implementation of home-based life-saving skills in Liberia, West Africa. J Midwifery Womens Health, 57(5), 495-501. doi:10.1111/j.1542-2011.2012.00172.x | 2012 | Journal article | ✓ | | | | |
| More, N. S., Bapat, U., Das, S., Alcock, G., Patil, S., Porel, M., . . . Osrin, D. (2012). Community mobilization in Mumbai slums to improve perinatal care and outcomes: a cluster randomized controlled trial. PLoS Med, 9(7), e1001257. doi:10.1371/journal.pmed.1001257 | 2012 | Journal article | ✓ | | | | |
| Moyo, S., Verver, S., Hawkridge, A., Geiter, L., Hatherill, M., Workman, L., . . . South African Tuberculosis Vaccine Initiative Neonatal Study, T. (2012). Tuberculosis case finding for vaccine trials in young children in high-incidence settings: a randomised trial. Int J Tuberc Lung Dis, 16(2), 185-191. doi:10.5588/ijtld.11.0348 | 2012 | Journal article | | | | ✓ | |

| Chronological reference list: Assessments included in the CBPHC review | | | | | | | |
|---|---|---|---|---|---|---|---|
| | | | Type of assessment carried out | | | | |
| | | | | Child health | | | |
| Reference | Year | Reference type | Mater-nal health | Neo-natal health | Health of children 1-59m | Health of 0-59-m-old children | Equity effect |
| Nahar, T., Azad, K., Aumon, B. H., Younes, L., Shaha, S., Kuddus, A., . . . Fottrell, E. (2012). Scaling up community mobilisation through women's groups for maternal and neonatal health: experiences from rural Bangladesh. BMC Pregnancy Childbirth, 12, 5. doi:10.1186/1471-2393-12-5 | 2012 | Journal article | ✓ | | | | |
| Ngabo, F., Nguimfack, J., Nwaigwe, F., Mugeni, C., Muhoza, D., Wilson, D. R., . . . Binagwaho, A. (2012). Designing and Implementing an Innovative SMS-based alert system (RapidSMS-MCH) to monitor pregnancy and reduce maternal and child deaths in Rwanda. Pan Afr Med J, 13, 31. | 2012 | Journal article | ✓ | | | | |
| Nonvignon, J., Chinbuah, M. A., Gyapong, M., Abbey, M., Awini, E., Gyapong, J. O., & Aikins, M. (2012). Is home management of fevers a cost-effective way of reducing under-five mortality in Africa? The case of a rural Ghanaian District. Trop Med Int Health, 17(8), 951-957. doi:10.1111/j.1365-3156.2012.03018.x | 2012 | Journal article | | | ✓ | | |
| Ohnmar, Tun, M., San, S., Than, W., & Chongsuvivatwong, V. (2012). Effects of malaria volunteer training on coverage and timeliness of diagnosis: a cluster randomized controlled trial in Myanmar. Malar J, 11, 309. doi:10.1186/1475-2875-11-309 | 2012 | Journal article | | | ✓ | | |
| Peletz, R., Simunyama, M., Sarenje, K., Baisley, K., Filteau, S., Kelly, P., & Clasen, T. (2012). Assessing water filtration and safe storage in households with young children of HIV-positive mothers: a randomized, controlled trial in Zambia. PLoS One, 7(10), e46548. doi:10.1371/journal.pone.0046548 | 2012 | Journal article | | | ✓ | | |
| Prata, N., Ejembi, C., Fraser, A., Shittu, O., & Minkler, M. (2012). Community mobilization to reduce postpartum hemorrhage in home births in northern Nigeria. Soc Sci Med, 74(8), 1288-1296. doi:10.1016/j.socscimed.2011.11.035 | 2012 | Journal article | ✓ | | | | |
| Rahman, F., Bose, S., Linnan, M., Rahman, A., Mashreky, S., Haaland, B., & Finkelstein, E. (2012). Cost-effectiveness of an injury and drowning prevention program in Bangladesh. Pediatrics, 130(6), e1621-1628. doi:10.1542/peds.2012-0757 | 2012 | Journal article | | | | ✓ | |
| Ratsimbasoa, A., Ravony, H., Vonimpaisomihanta, J. A., Raherinjafy, R., Jahevitra, M., Rapelanoro, R., . . . Menard, D. (2012). Compliance, safety, and effectiveness of fixed-dose artesunate-amodiaquine for presumptive treatment of non-severe malaria in the context of home management of malaria in Madagascar. Am J Trop Med Hyg, 86(2), 203-210. doi:10.4269/ajtmh.2012.11-0047 | 2012 | Journal article | | | ✓ | | |
| Ratsimbasoa, A., Ravony, H., Vonimpaisomihanta, J. A., Raherinjafy, R., Jahevitra, M., Rapelanoro, R., . . . Menard, D. (2012). Management of uncomplicated malaria in febrile under five-year-old children by community health workers in Madagascar: reliability of malaria rapid diagnostic tests. Malar J, 11, 85. doi:10.1186/1475-2875-11-85 | 2012 | Journal article | | | ✓ | | |

## Chronological reference list: Assessments included in the CBPHC review

| Reference | Year | Reference type | Type of assessment carried out | | | | |
|---|---|---|---|---|---|---|---|
| | | | | Child health | | | |
| | | | Maternal health | Neonatal health | Health of children 1-59m | Health of 0-59-m-old children | Equity effect |
| Satti, H., McLaughlin, M. M., Omotayo, D. B., Keshavjee, S., Becerra, M. C., Mukherjee, J. S., & Seung, K. J. (2012). Outcomes of comprehensive care for children empirically treated for multidrug-resistant tuberculosis in a setting of high HIV prevalence. PLoS One, 7(5), e37114. doi:10.1371/journal.pone.0037114 | 2012 | Journal article | | | | ✓ | |
| Sivhaga, K., Hlabano, B., & Odhiambo, P. O. (2012). Using partnership approach to reduce mortality and morbidity among children under five in Limpopo province, South Africa. Pan Afr Med J, 13 Suppl 1, 14. | 2012 | Journal article | | | | ✓ | |
| Soofi, S., Ahmed, S., Fox, M. P., MacLeod, W. B., Thea, D. M., Qazi, S. A., & Bhutta, Z. A. (2012). Effectiveness of community case management of severe pneumonia with oral amoxicillin in children aged 2-59 months in Matiari district, rural Pakistan: a cluster-randomised controlled trial. Lancet, 379(9817), 729-737. doi:10.1016/S0140-6736(11)61714-5 | 2012 | Journal article | | | | ✓ | |
| Soofi, S., Cousens, S., Imdad, A., Bhutto, N., Ali, N., & Bhutta, Z. A. (2012). Topical application of chlorhexidine to neonatal umbilical cords for prevention of omphalitis and neonatal mortality in a rural district of Pakistan: a community-based, cluster-randomised trial. Lancet, 379(9820), 1029-1036. doi:10.1016/S0140-6736(11)61877-1 | 2012 | Journal article | | ✓ | | | |
| Stauber, C. E., Kominek, B., Liang, K. R., Osman, M. K., & Sobsey, M. D. (2012). Evaluation of the impact of the plastic BioSand filter on health and drinking water quality in rural Tamale, Ghana. Int J Environ Res Public Health, 9(11), 3806-3823. doi:10.3390/ijerph9113806 | 2012 | Journal article | | | ✓ | | |
| Suchdev, P. S., Ruth, L. J., Woodruff, B. A., Mbakaya, C., Mandava, U., Flores-Ayala, R., . . . Quick, R. (2012). Selling Sprinkles micronutrient powder reduces anemia, iron deficiency, and vitamin A deficiency in young children in Western Kenya: a cluster-randomized controlled trial. Am J Clin Nutr, 95(5), 1223-1230. doi:10.3945/ajcn.111.030072 | 2012 | Journal article | | | | ✓ | |
| Wangalwa, G., Cudjoe, B., Wamalwa, D., Machira, Y., Ofware, P., Ndirangu, M., & Ilako, F. (2012). Effectiveness of Kenya's Community Health Strategy in delivering community-based maternal and newborn health care in Busia County, Kenya: non-randomized pre-test post test study. Pan Afr Med J, 13 Suppl 1, 12. | 2012 | Journal article | ✓ | ✓ | | | |
| Wilford, R., Golden, K., & Walker, D. G. (2012). Cost-effectiveness of community-based management of acute malnutrition in Malawi. Health Policy Plan, 27(2), 127-137. doi:10.1093/heapol/czr017 | 2012 | Journal article | | | | ✓ | |
| World Relief/Burundi (2012). Ramba Kibondo "Live Long Child" Child Survival Project: Final evaluation project, Kibuye Health District, Gitega Province, Burundi. | 2012 | Report | | | | ✓ | |
| World Renew/India (2012). FInal report on the Parivartan ("Transformation") Child Survival Project, Sahibganj district, Jarkhand state, India. | 2012 | Report | | | | ✓ | |
| Younes, L., Houweling, T. A., Azad, K., Costello, A., & Fottrell, E. (2012). Estimating coverage of a women's | 2012 | Journal article | | ✓ | | | |

| Chronological reference list: Assessments included in the CBPHC review | | | | | | | |
|---|---|---|---|---|---|---|---|
| | | | Type of assessment carried out | | | | |
| | | | | Child health | | | |
| Reference | Year | Reference type | Mater-nal health | Neo-natal health | Health of children 1-59m | Health of 0-59-m-old children | Equity effect |
| group intervention among a population of pregnant women in rural Bangladesh. BMC Pregnancy Childbirth, 12, 60. doi:10.1186/1471-2393-12-60 | | | | | | | |
| Callaghan-Koru, J. A., Nonyane, B. A., Guenther, T., Sitrin, D., Ligowe, R., Chimbalanga, E., . . . Baqui, A. H. (2013). Contribution of community-based newborn health promotion to reducing inequities in healthy newborn care practices and knowledge: evidence of improvement from a three-district pilot program in Malawi. BMC Public Health, 13, 1052. doi:10.1186/1471-2458-13-1052 | 2013 | Journal article | ✓ | | | ✓ | ✓ |
| Carlo, W. A., Goudar, S. S., Pasha, O., Chomba, E., Wallander, J. L., Biasini, F. J., . . . Children's Health Research, I. (2013). Randomized trial of early developmental intervention on outcomes in children after birth asphyxia in developing countries. J Pediatr, 162(4), 705-712 e703. doi:10.1016/j.jpeds.2012.09.052 | 2013 | Journal article | | | | ✓ | |
| Chinbuah, M. A., Adjuik, M., Cobelens, F., Koram, K. A., Abbey, M., Gyapong, M., . . . Gyapong, J. O. (2013). Impact of treating young children with antimalarials with or without antibiotics on morbidity: a cluster-randomized controlled trial in Ghana. Int Health, 5(3), 228-235. doi:10.1093/inthealth/iht021 | 2013 | Journal article | | | | ✓ | |
| Colbourn, T., Nambiar, B., Bondo, A., Makwenda, C., Tsetekani, E., Makonda-Ridley, A., . . . Costello, A. (2013). Effects of quality improvement in health facilities and community mobilization through women's groups on maternal, neonatal and perinatal mortality in three districts of Malawi: MaiKhanda, a cluster randomized controlled effectiveness trial. Int Health, 5(3), 180-195. doi:10.1093/inthealth/iht011 | 2013 | Journal article | ✓ | ✓ | | | |
| Curamericas Global/Liberia (2013). Final evaluation for Nehnwaa child survival project: Census-Based Impact-Oriented methodology for community-based primary health care in Nimba country, Liberia. | 2013 | Report | ✓ | | | ✓ | |
| Davis, T. P., Jr., Wetzel, C., Hernandez Avilan, E., de Mendoza Lopes, C., Chase, R. P., Winch, P. J., & Perry, H. B. (2013). Reducing child global undernutrition at scale in Sofala Province, Mozambique, using Care Group Volunteers to communicate health messages to mothers. Glob Health Sci Pract, 1(1), 35-51. doi:10.9745/GHSP-D-12-00045 | 2013 | Journal article | | | | ✓ | |
| Episcopal Relief & Development/Uganda. (2013). Ajula Pa Rwot (Child Survival) Project in Northern Uganda - Amuru and Gulu Districts. | 2013 | Report | | | | ✓ | |
| Findley, S. E., Uwemedimo, O. T., Doctor, H. V., Green, C., Adamu, F., & Afenyadu, G. Y. (2013). Comparison of high- versus low-intensity community health worker intervention to promote newborn and child health in Northern Nigeria. Int J Womens Health, 5, 717-728. doi:10.2147/IJWH.S49785 | 2013 | Journal article | ✓ | ✓ | | | |
| Fottrell, E., Azad, K., Kuddus, A., Younes, L., Shaha, S., Nahar, T., . . . Houweling, T. A. (2013). The effect of increased coverage of participatory women's groups on neonatal mortality in Bangladesh: A cluster | 2013 | Journal article | | ✓ | | | |

| Chronological reference list: Assessments included in the CBPHC review | | | | | | | |
|---|---|---|---|---|---|---|---|
| | | | Type of assessment carried out | | | | |
| | | | | Child health | | | |
| Reference | Year | Reference type | Maternal health | Neonatal health | Health of children 1-59m | Health of 0-59-m-old children | Equity effect |
| randomized trial. JAMA Pediatr, 167(9), 816-825. doi:10.1001/jamapediatrics.2013.2534 | | | | | | | |
| Gilroy, K. E., Callaghan-Koru, J. A., Cardemil, C. V., Nsona, H., Amouzou, A., Mtimuni, A., . . . Group, C. C.-M. Q. o. C. W. (2013). Quality of sick child care delivered by Health Surveillance Assistants in Malawi. Health Policy Plan, 28(6), 573-585. doi:10.1093/heapol/czs095 | 2013 | Journal article | | | | ✓ | |
| Gregson, S., Nyamukapa, C. A., Sherr, L., Mugurungi, O., & Campbell, C. (2013). Grassroots community organizations' contribution to the scale-up of HIV testing and counselling services in Zimbabwe. AIDS, 27(10), 1657-1666. doi:10.1097/QAD.0b013e3283601b90 | 2013 | Journal article | | | | ✓ | |
| Gupta, N., Cyamatare, F. R., Niyigena, P., Niyigena, J. W., Stulac, S., Mugwaneza, P., . . . Franke, M. F. (2013). Clinical outcomes of a comprehensive integrated program for HIV-exposed infants: a 3-year experience promoting HIV-free survival in rural Rwanda. J Acquir Immune Defic Syndr, 62(4), e109-114. doi:10.1097/QAI.0b013e31827d5118 | 2013 | Journal article | | ✓ | ✓ | | |
| Habib, M. A., Soofi, S., Sadiq, K., Samejo, T., Hussain, M., Mirani, M., . . . Bhutta, Z. A. (2013). A study to evaluate the acceptability, feasibility and impact of packaged interventions ("Diarrhea Pack") for prevention and treatment of childhood diarrhea in rural Pakistan. BMC Public Health, 13, 922. doi:10.1186/1471-2458-13-922 | 2013 | Journal article | | | | ✓ | |
| Havemann, K., Pridmore, P., Tomkins, A., & Garn, K. D. (2013). What works and why? Evaluation of a community nutrition programme in Kenya. Public Health Nutr, 16(9), 1614-1621. doi:10.1017/S1368980012004880 | 2013 | Journal article | | | | ✓ | |
| Johnson, A. D., Thomson, D. R., Atwood, S., Alley, I., Beckerman, J. L., Kone, I., . . . Mukherjee, J. (2013). Assessing early access to care and child survival during a health system strengthening intervention in Mali: a repeated cross sectional survey. PLoS One, 8(12), e81304. doi:10.1371/journal.pone.0081304 | 2013 | Journal article | | | ✓ | | |
| Kalyango, J. N., Alfven, T., Peterson, S., Mugenyi, K., Karamagi, C., & Rutebemberwa, E. (2013). Integrated community case management of malaria and pneumonia increases prompt and appropriate treatment for pneumonia symptoms in children under five years in Eastern Uganda. Malar J, 12, 340. doi:10.1186/1475-2875-12-340 | 2013 | Journal article | | | ✓ | | |
| Kapungu, C. T., Mensah-Homiah, J., Akosah, E., Asare, G., Carnahan, L., Frimpong, M. A., . . . Ghana, P. P. H. S. G. (2013). A community-based continuum of care model for the prevention of postpartum hemorrhage in rural Ghana. Int J Gynaecol Obstet, 120(2), 156-159. doi:10.1016/j.ijgo.2012.08.021 | 2013 | Journal article | ✓ | | | | |

## Chronological reference list: Assessments included in the CBPHC review

| Reference | Year | Reference type | Type of assessment carried out | | | | |
|---|---|---|---|---|---|---|---|
| | | | | Child health | | | |
| | | | Mater-nal health | Neo-natal health | Health of children 1-59m | Health of 0-59-m-old children | Equity effect |
| Karim, A. M., Admassu, K., Schellenberg, J., Alemu, H., Getachew, N., Ameha, A., . . . Betemariam, W. (2013). Effect of ethiopia's health extension program on maternal and newborn health care practices in 101 rural districts: a dose-response study. PLoS One, 8(6), e65160. doi:10.1371/journal.pone.0065160 | 2013 | Journal article | ✓ | | | | |
| Keoprasith, B., Kizuki, M., Watanabe, M., & Takano, T. (2013). The impact of community-based, workshop activities in multiple local dialects on the vaccination coverage, sanitary living and the health status of multiethnic populations in Lao PDR. Health Promot Int, 28(3), 453-465. doi:10.1093/heapro/das030 | 2013 | Journal article | | | | ✓ | |
| Khan, M. H., Khalique, N., Siddiqui, A. R., & Amir, A. (2013). Impact of behavior change communication among pregnant women regarding neonatal care. Indian J Pediatr, 80(10), 804-808. doi:10.1007/s12098-013-1076-x | 2013 | Journal article | | ✓ | | | |
| Kirkwood, B. R., Manu, A., ten Asbroek, A. H., Soremekun, S., Weobong, B., Gyan, T., . . . Hill, Z. (2013). Effect of the Newhints home-visits intervention on neonatal mortality rate and care practices in Ghana: a cluster randomised controlled trial. Lancet, 381(9884), 2184-2192. doi:10.1016/S0140-6736(13)60095-1 | 2013 | Journal article | | ✓ | | | |
| Littrell, M., Moukam, L. V., Libite, R., Youmba, J. C., & Baugh, G. (2013). Narrowing the treatment gap with equitable access: mid-term outcomes of a community case management program in Cameroon. Health Policy Plan, 28(7), 705-716. doi:10.1093/heapol/czs110 | 2013 | Journal article | | | ✓ | | ✓ |
| Livingston, A., Tomedi, A., Campbell, A., Morales, C., & Mwanthi, M. A. (2013). A community health worker home visitation project to prevent neonatal deaths in Kenya. J Trop Pediatr, 59(1), 64-66. doi:10.1093/tropej/fms034 | 2013 | Journal article | | | | ✓ | |
| Lori, J. R., Munro, M. L., Rominski, S., Williams, G., Dahn, B. T., Boyd, C. J., . . . Gwenegale, W. (2013). Maternity waiting homes and traditional midwives in rural Liberia. Int J Gynaecol Obstet, 123(2), 114-118. doi:10.1016/j.ijgo.2013.05.024 | 2013 | Journal article | ✓ | | | | |
| Mullany, L. C., Shah, R., El Arifeen, S., Mannan, I., Winch, P. J., Hill, A., . . . Baqui, A. H. (2013). Chlorhexidine cleansing of the umbilical cord and separation time: a cluster-randomized trial. Pediatrics, 131(4), 708-715. doi:10.1542/peds.2012-2951 | 2013 | Journal article | | ✓ | | | |
| Nalwadda, C. K., Waiswa, P., Kiguli, J., Namazzi, G., Namutamba, S., Tomson, G., . . . Guwatudde, D. (2013). High compliance with newborn community-to-facility referral in eastern Uganda:.an opportunity to improve newborn survival. PLoS One, 8(11), e81610. doi:10.1371/journal.pone.0081610 | 2013 | Journal article | | ✓ | | | |
| Nanyonjo, A., Makumbi, F., Etou, P., Tomson, G., Kallander, K., & in, S. S. G. (2013). Perceived quality of care for common childhood illnesses: facility versus | 2013 | Journal article | | | ✓ | | |

| Chronological reference list: Assessments included in the CBPHC review | | | | | | | |
|---|---|---|---|---|---|---|---|
| | | | Type of assessment carried out | | | | |
| | | | | Child health | | | |
| Reference | Year | Reference type | Mater-nal health | Neo-natal health | Health of children 1-59m | Health of 0-59-m-old children | Equity effect |
| community based providers in Uganda. PLoS One, 8(11), e79943. doi:10.1371/journal.pone.0079943 | | | | | | | |
| Navarro, J. I., Sigulem, D. M., Ferraro, A. A., Polanco, J. J., & Barros, A. J. (2013). The double task of preventing malnutrition and overweight: a quasi-experimental community-based trial. BMC Public Health, 13, 212. doi:10.1186/1471-2458-13-212 | 2013 | Journal article | | | | ✓ | |
| Parashar, M., Singh, S., Kishore, J., Kumar, A., & Bhardwaj, M. (2013). Effect of Community-based Behavior Change Communication on Delivery and Newborn Health Care Practices in a Resettlement Colony of Delhi. Indian J Community Med, 38(1), 42-48. doi:10.4103/0970-0218.106627 | 2013 | Journal article | | | | ✓ | |
| Persson, L. A., Nga, N. T., Malqvist, M., Thi Phuong Hoa, D., Eriksson, L., Wallin, L., . . . Ewald, U. (2013). Effect of Facilitation of Local Maternal-and-Newborn Stakeholder Groups on Neonatal Mortality: Cluster-Randomized Controlled Trial. PLoS Med, 10(5), e1001445. doi:10.1371/journal.pmed.1001445 | 2013 | Journal article | | ✓ | | | |
| Radhakrishna, K. V., Hemalatha, R., Geddam, J. J., Kumar, P. A., Balakrishna, N., & Shatrugna, V. (2013). Effectiveness of zinc supplementation to full term normal infants: a community based double blind, randomized, controlled, clinical trial. PLoS One, 8(5), e61486. doi:10.1371/journal.pone.0061486 | 2013 | Journal article | | | | ✓ | |
| Ramsey, K., Hingora, A., Kante, M., Jackson, E., Exavery, A., Pemba, S., . . . Phillips, J. F. (2013). The Tanzania Connect Project: a cluster-randomized trial of the child survival impact of adding paid community health workers to an existing facility-focused health system. BMC Health Serv Res, 13 Suppl 2, S6. doi:10.1186/1472-6963-13-S2-S6 | 2013 | Journal article | | ✓ | | | |
| Roy, S. S., Mahapatra, R., Rath, S., Bajpai, A., Singh, V., Rath, S., . . . Prost, A. (2013). Improved neonatal survival after participatory learning and action with women's groups: a prospective study in rural eastern India. Bull World Health Organ, 91(6), 426-433B. doi:10.2471/BLT.12.105171 | 2013 | Journal article | | | | ✓ | |
| Save the Children/Ethiopia (2013). Innovation for Scale- Enhancing Ethiopia's health extentions package in the Southerm Nations and Nationalities People's Region (SNNPR): Report of the final evaluation. | 2013 | Report | | | | ✓ | |
| Shewade, H. D., Patro, B. K., Bharti, B., Soundappan, K., Kaur, A., & Taneja, N. (2013). Effectiveness of indigenous ready-to-use therapeutic food in community-based management of uncomplicated severe acute malnutrition: a randomized controlled trial from India. J Trop Pediatr, 59(5), 393-398. doi:10.1093/tropej/fmt039 | 2013 | Journal article | | | | ✓ | |
| Siekmans, K., Sohani, S., Kisia, J., Kiilu, K., Wamalwa, E., Nelima, F., . . . Ngindu, A. (2013). Community case management of malaria: a pro-poor intervention in rural Kenya. Int Health, 5(3), 196-204. doi:10.1093/inthealth/iht017 | 2013 | Journal article | | | ✓ | | |

| Chronological reference list: Assessments included in the CBPHC review | | | | | | | |
|---|---|---|---|---|---|---|---|
| | | | Type of assessment carried out | | | | |
| | | | | Child health | | | |
| Reference | Year | Reference type | Mater-nal health | Neo-natal health | Health of children 1-59m | Health of 0-59-m-old children | Equity effect |
| Stanton, C. K., Newton, S., Mullany, L. C., Cofie, P., Tawiah Agyemang, C., Adiibokah, E., . . . Owusu-Agyei, S. (2013). Effect on postpartum hemorrhage of prophylactic oxytocin (10 IU) by injection by community health officers in Ghana: a community-based, cluster-randomized trial. PLoS Med, 10(10), e1001524. doi:10.1371/journal.pmed.1001524 | 2013 | Journal article | ✓ | | | | |
| USAID/Burundi (2013). Community health systems strengthening in Cibitoke province, Burundi: Mabayi. Child survival project final evaluation report. | 2013 | Report | ✓ | | | ✓ | |
| Vazir, S., Engle, P., Balakrishna, N., Griffiths, P. L., Johnson, S. L., Creed-Kanashiro, H., . . . Bentley, M. E. (2013). Cluster-randomized trial on complementary and responsive feeding education to caregivers found improved dietary intake, growth and development among rural Indian toddlers. Matern Child Nutr, 9(1), 99-117. doi:10.1111/j.1740-8709.2012.00413.x | 2013 | Journal article | | | | ✓ | |
| Vir, S. C. (2013). Community based maternal and child health nutrition project, uttar pradesh: an innovative strategy focusing on "at risk" families. Indian J Community Med, 38(4), 234-239. doi:10.4103/0970-0218.120159 | 2013 | Journal article | | ✓ | | | |
| World Vision/Afghanistan (2013). Better Health for Afghan Mother and Children (BHAMC) Project: Final Evaluation project, Karukh, Zindajan, Kohsan and Chisht-e-Sharif districts, Herat province, Afghanistan. | 2013 | Report | | | | ✓ | |
| Wajid, A., White, F., & Karim, M. S. (2013). Community health workers and health care delivery: evaluation of a women's reproductive health care project in a developing country. PLoS One, 8(9), e75476. doi:10.1371/journal.pone.0075476 | 2013 | Journal article | ✓ | | | | |
| Adam, M. B., Dillmann, M., Chen, M. K., Mbugua, S., Ndung'u, J., Mumbi, P., . . . Meissner, P. (2014). Improving maternal and newborn health: effectiveness of a community health worker program in rural Kenya. PLoS One, 9(8), e104027. doi:10.1371/journal.pone.0104027 | 2014 | Journal article | ✓ | | | | |
| Afsana, K., Haque, M. R., Sobhan, S., & Shahin, S. A. (2014). BRAC's experience in scaling-up MNP in Bangladesh. Asia Pac J Clin Nutr, 23(3), 377-384. doi:10.6133/apjcn.2014.23.3.22 | 2014 | Journal article | | | | ✓ | |
| Aga Khan Foundation/Pakistan (2014). Chitral Child Survival Project, Chitral, Khyber Pakhtunkhwa, Pakistan: Final Evaluation. | 2014 | Report | ✓ | | | | |
| Aga Khan Foundation/USA (2014). Chitral child survival project, Chitral, Khber Pakhtunkhwa, Pakistan: Final evaluation. | 2014 | Report | | ✓ | | | |
| Amano, S., Shrestha, B. P., Chaube, S. S., Higuchi, M., Manandhar, D. S., Osrin, D., . . . Saville, N. (2014). Effectiveness of female community health volunteers in the detection and management of low-birth-weight in Nepal. Rural Remote Health, 14(1), 2508. | 2014 | Journal article | | ✓ | | | |
| Ameha, A., Karim, A. M., Erbo, A., Ashenafi, A., Hailu, M., Hailu, B., . . . Betemariam, W. (2014). Effectiveness of supportive supervision on the consistency of | 2014 | Journal article | | | | ✓ | |

| Chronological reference list: Assessments included in the CBPHC review | | | | | | | | |
|---|---|---|---|---|---|---|---|---|
| | | | Type of assessment carried out | | | | | |
| | | | | Child health | | | | |
| Reference | Year | Reference type | Mater-nal health | Neo-natal health | Health of children 1-59m | Health of 0-59-m-old children | Equity effect |
| integrated community cases management skills of the health extension workers in 113 districts of Ethiopia. Ethiop Med J, 52 Suppl 3, 65-71. | | | | | | | |
| Amouzou, A., Morris, S., Moulton, L. H., & Mukanga, D. (2014). Assessing the impact of integrated community case management (iCCM) programs on child mortality: Review of early results and lessons learned in sub-Saharan Africa. J Glob Health, 4(2), 020411. doi:10.7189/jogh.04.020411 | 2014 | Journal article | | | | ✓ | |
| Ansah Manu, A., ten Asbroek, A., Soremekun, S., Gyan, T., Weobong, B., Tawiah-Agyemang, C., . . . Kirkwood, B. R. (2014). Evaluating the implementation of community volunteer assessment and referral of sick babies: lessons learned from the Ghana Newhints home visits cluster randomized controlled trial. Health Policy Plan, 29 Suppl 2, ii114-127. doi:10.1093/heapol/czu080 | 2014 | Journal article | | ✓ | | | |
| Ayuku, D., Embleton, L., Koech, J., Atwoli, L., Hu, L., Ayaya, S., . . . Braitstein, P. (2014). The government of Kenya cash transfer for orphaned and vulnerable children: cross-sectional comparison of household and individual characteristics of those with and without. BMC Int Health Hum Rights, 14, 25. doi:10.1186/1472-698X-14-25 | 2014 | Journal article | | | | ✓ | |
| Coutinho, S. B., Lira, P. I., Lima, M. C., Frias, P. G., Eickmann, S. H., & Ashworth, A. (2014). Promotion of exclusive breast-feeding at scale within routine health services: impact of breast-feeding counselling training for community health workers in Recife, Brazil. Public Health Nutr, 17(4), 948-955. doi:10.1017/S1368980013001833 | 2014 | Journal article | | ✓ | | | |
| Das, A., Friedman, J., Kandpal, E., Ramana, G. N., Gupta, R. K., Pradhan, M. M., & Govindaraj, R. (2014). Strengthening malaria service delivery through supportive supervision and community mobilization in an endemic Indian setting: an evaluation of nested delivery models. Malar J, 13, 482. doi:10.1186/1475-2875-13-482 | 2014 | Journal article | | | ✓ | | |
| Fatti, G., Shaikh, N., Eley, B., & Grimwood, A. (2014). Improved virological suppression in children on antiretroviral treatment receiving community-based adherence support: a multicentre cohort study from South Africa. AIDS Care, 26(4), 448-453. doi:10.1080/09540121.2013.855699 | 2014 | Journal article | | | ✓ | | |
| Gill, C. J., MacLeod, W. B., Phiri-Mazala, G., Guerina, N. G., Mirochnick, M., Knapp, A. B., & Hamer, D. H. (2014). Can traditional birth attendants be trained to accurately identify septic infants, initiate antibiotics, and refer in a rural African setting? Glob Health Sci Pract, 2(3), 318-327. doi:10.9745/GHSP-D-14-00045 | 2014 | Journal article | | ✓ | | | |
| Hamainza, B., Moonga, H., Sikaala, C. H., Kamuliwo, M., Bennett, A., Eisele, T. P., . . . Killeen, G. F. (2014). Monitoring, characterization and control of chronic, symptomatic malaria infections in rural Zambia through monthly household visits by paid community | 2014 | Journal article | | | | ✓ | |

| Chronological reference list: Assessments included in the CBPHC review | | | | | | | |
|---|---|---|---|---|---|---|---|
| | | | Type of assessment carried out | | | | |
| | | | | Child health | | | |
| Reference | Year | Reference type | Maternal health | Neonatal health | Health of children 1-59m | Health of 0-59-m-old children | Equity effect |
| health workers. Malar J, 13, 128. doi:10.1186/1475-2875-13-128 | | | | | | | |
| Langston, A., Weiss, J., Landegger, J., Pullum, T., Morrow, M., Kabadege, M., . . . Sarriot, E. (2014). Plausible role for CHW peer support groups in increasing care-seeking in an integrated community case management project in Rwanda: a mixed methods evaluation. Glob Health Sci Pract, 2(3), 342-354. doi:10.9745/GHSP-D-14-00067 | 2014 | Journal article | ✓ | | | ✓ | |
| le Roux, I. M., Rotheram-Borus, M. J., Stein, J., & Tomlinson, M. (2014). The impact of paraprofessional home visitors on infants' growth and health at 18 months. Vulnerable Child Youth Stud, 9(4), 291-304. doi:10.1080/17450128.2014.940413 | 2014 | Journal article | | | | ✓ | |
| Lema, I. A., Sando, D., Magesa, L., Machumi, L., Mungure, E., Mwanyika Sando, M., . . . Barnighausen, T. (2014). Community health workers to improve antenatal care and PMTCT uptake in Dar es Salaam, Tanzania: a quantitative performance evaluation. J Acquir Immune Defic Syndr, 67 Suppl 4, S195-201. doi:10.1097/QAI.0000000000000371 | 2014 | Journal article | | | | ✓ | |
| Lopes, S. C., Cabral, A. J., & de Sousa, B. (2014). Community health workers: to train or to restrain? A longitudinal survey to assess the impact of training community health workers in the Bolama Region, Guinea-Bissau. Hum Resour Health, 12, 8. doi:10.1186/1478-4491-12-8 | 2014 | Journal article | | | | ✓ | |
| Matovu, F., Nanyiti, A., & Rutebemberwa, E. (2014). Household health care-seeking costs: experiences from a randomized, controlled trial of community-based malaria and pneumonia treatment among under-fives in eastern Uganda. Malar J, 13, 222. doi:10.1186/1475-2875-13-222 | 2014 | Journal article | | | ✓ | | |
| Mayhew, M., Ickx, P., Stanekzai, H., Mashal, T., & Newbrander, W. (2014). Improving nutrition in Afghanistan through a community-based growth monitoring and promotion programme: a pre-post evaluation in five districts. Glob Public Health, 9 Suppl 1, S58-75. doi:10.1080/17441692.2014.917194 | 2014 | Journal article | | | | ✓ | |
| Mazumder, S., Taneja, S., Bahl, R., Mohan, P., Strand, T. A., Sommerfelt, H., . . . Childhood Illness Evaluation Study, G. (2014). Effect of implementation of integrated management of neonatal and childhood illness programme on treatment seeking practices for morbidities in infants: cluster randomised trial. BMJ, 349, g4988. doi:10.1136/bmj.g4988 | 2014 | Journal article | | | ✓ | | |
| Miller, L. C., Joshi, N., Lohani, M., Rogers, B., Loraditch, M., Houser, R., . . . Mahato, S. (2014). Community development and livestock promotion in rural Nepal: effects on child growth and health. Food Nutr Bull, 35(3), 312-326. doi:http://dx.doi.org/10.1177/156482651403500304 | 2014 | Journal article | | | | ✓ | |
| Mkumbo, E., Hanson, C., Penfold, S., Manzi, F., & Schellenberg, J. (2014). Innovation in supervision and support of community health workers for better | 2014 | Journal article | | ✓ | | | |

| Chronological reference list: Assessments included in the CBPHC review | | | | | | | |
|---|---|---|---|---|---|---|---|
| | | | Type of assessment carried out | | | | |
| | | | | Child health | | | |
| Reference | Year | Reference type | Mater-nal health | Neo-natal health | Health of children 1-59m | Health of 0-59-m-old children | Equity effect |
| newborn survival in southern Tanzania. Int Health, 6(4), 339-341. doi:10.1093/inthealth/ihu016 | | | | | | | |
| Moseson, H., Hamad, R., & Fernald, L. (2014). Microcredit participation and child health: results from a cross-sectional study in Peru. J Epidemiol Community Health, 68(12), 1175-1181. doi:10.1136/jech-2014-204071 | 2014 | Journal article | | | | ✓ | |
| Mugeni, C., Levine, A. C., Munyaneza, R. M., Mulindahabi, E., Cockrell, H. C., Glavis-Bloom, J., . . . Binagwaho, A. (2014). Nationwide implementation of integrated community case management of childhood illness in Rwanda. Glob Health Sci Pract, 2(3), 328-341. doi:10.9745/GHSP-D-14-00080 | 2014 | Journal article | | | | ✓ | |
| Penfold, S., Manzi, F., Mkumbo, E., Temu, S., Jaribu, J., Shamba, D. D., . . . Schellenberg, J. A. (2014). Effect of home-based counselling on newborn care practices in southern Tanzania one year after implementation: a cluster-randomised controlled trial. BMC Pediatr, 14, 187. doi:10.1186/1471-2431-14-187 | 2014 | Journal article | | ✓ | | | |
| Rasooly, M. H., Govindasamy, P., Aqil, A., Rutstein, S., Arnold, F., Noormal, B., . . . Shadoul, A. (2014). Success in reducing maternal and child mortality in Afghanistan. Glob Public Health, 9 Suppl 1, S29-42. doi:10.1080/17441692.2013.827733 | 2014 | Journal article | | | | ✓ | |
| Rotheram-Borus, M. J., Tomlinson, M., le Roux, I. M., Harwood, J. M., Comulada, S., O'Connor, M. J., . . . Worthman, C. M. (2014). A cluster randomised controlled effectiveness trial evaluating perinatal home visiting among South African mothers/infants. PLoS One, 9(10), e105934. doi:10.1371/journal.pone.0105934 | 2014 | Journal article | ✓ | ✓ | | | |
| Saleem, A. F., Mahmud, S., Baig-Ansari, N., & Zaidi, A. K. (2014). Impact of maternal education about complementary feeding on their infants' nutritional outcomes in low- and middle-income households: a community-based randomized interventional study in Karachi, Pakistan. J Health Popul Nutr, 32(4), 623-633. | 2014 | Journal article | | | | ✓ | |
| Save the Children/Zambia (2014). Lufwanyama Integrated Neonatal and Child Health Project in Zambia (LINCHPIN): Final Evaluation. | 2014 | Report | ✓ | ✓ | | | |
| Seim, A. R., Alassoum, Z., Bronzan, R. N., Mainassara, A. A., Jacobsen, J. L., & Gali, Y. A. (2014). Pilot community-mobilization program reduces maternal and perinatal mortality and prevents obstetric fistula in Niger. Int J Gynaecol Obstet, 127(3), 269-274. doi:10.1016/j.ijgo.2014.06.016 | 2014 | Journal article | ✓ | ✓ | | | |
| Singh, K., Brodish, P., & Haney, E. (2014). Postnatal care by provider type and neonatal death in sub-Saharan Africa: a multilevel analysis. BMC Public Health, 14, 941. doi:10.1186/1471-2458-14-941 | 2014 | Journal article | | ✓ | | | |
| Smith, J. M., Baawo, S. D., Subah, M., Sirtor-Gbassie, V., Howe, C. J., Ishola, G., . . . Dwivedi, V. (2014). Advance distribution of misoprostol for prevention of postpartum hemorrhage (PPH) at home births in two | 2014 | Journal article | ✓ | | | | |

| Chronological reference list: Assessments included in the CBPHC review | | | | | | | | |
|---|---|---|---|---|---|---|---|---|
| | | | Type of assessment carried out | | | | | |
| | | | | Child health | | | | |
| Reference | Year | Reference type | Mater-nal health | Neo-natal health | Health of children 1-59m | Health of 0-59-m-old children | Equity effect |
| districts of Liberia. BMC Pregnancy Childbirth, 14, 189. doi:10.1186/1471-2393-14-189 | | | | | | | |
| Smith, J. M., Dimiti, A., Dwivedi, V., Ochieng, I., Dalaka, M., Currie, S., . . . McKaig, C. (2014). Advance distribution of misoprostol for the prevention of postpartum hemorrhage in South Sudan. Int J Gynaecol Obstet, 127(2), 183-188. doi:10.1016/j.ijgo.2014.05.016 | 2014 | Journal article | ✓ | | | | |
| Tine, R. C., Ndour, C. T., Faye, B., Cairns, M., Sylla, K., Ndiaye, M., . . . Gaye, O. (2014). Feasibility, safety and effectiveness of combining home based malaria management and seasonal malaria chemoprevention in children less than 10 years in Senegal: a cluster-randomised trial. Trans R Soc Trop Med Hyg, 108(1), 13-21. doi:10.1093/trstmh/trt103 | 2014 | Journal article | | | ✓ | | |
| Tomlinson, M., Doherty, T., Ijumba, P., Jackson, D., Lawn, J., Persson, L. A., . . . Chopra, M. (2014). Goodstart: a cluster randomised effectiveness trial of an integrated, community-based package for maternal and newborn care, with prevention of mother-to-child transmission of HIV in a South African township. Trop Med Int Health, 19(3), 256-266. doi:10.1111/tmi.12257 | 2014 | Journal article | | | | ✓ | |
| Vir, S. C., Kalita, A., Mondal, S., & Malik, R. (2014). Impact of community-based mitanin programme on undernutrition in rural Chhattisgarh State, India. Food Nutr Bull, 35(1), 83-91. doi:http://dx.doi.org/10.1177/156482651403500110 | 2014 | Journal article | | | | ✓ | |
| Wogi, A., Teno, D., Bulto, T., Deressa, W., Alemu, H., & Nigussie, M. (2014). Effect of integrated community case management of common childhood illnesses on the quality of malaria case management provided by health extension workers at health posts. Ethiop Med J, 52 Suppl 3, 99-108. | 2014 | Journal article | | | | ✓ | |
| Yansaneh, A. I., Moulton, L. H., George, A. S., Rao, S. R., Kennedy, N., Bangura, P., . . . Diaz, T. (2014). Influence of community health volunteers on care seeking and treatment coverage for common childhood illnesses in the context of free health care in rural Sierra Leone. Trop Med Int Health, 19(12), 1466-1476. doi:10.1111/tmi.12383 | 2014 | Journal article | | | | ✓ | |
| Abbey, M., Bartholomew, L. K., Pappoe, M., & van den Borne, B. (2015). Treating fever in children under 5 years of age: caregiver perceptions of community health worker services in Dangme West district, Ghana. Int Health, 7(6), 455-463. doi:10.1093/inthealth/ihv027 | 2015 | Journal article | | | | ✓ | |
| Adams, A. M., Nababan, H. Y., & Hanifi, S. M. (2015). Building social networks for maternal and newborn health in poor urban settlements: a cross-sectional study in Bangladesh. PLoS One, 10(4), e0123817. doi:10.1371/journal.pone.0123817 | 2015 | Journal article | ✓ | | | | |
| Ahmed, S., Ahmed, S., McKaig, C., Begum, N., Mungia, J., Norton, M., & Baqui, A. H. (2015). The Effect of Integrating Family Planning with a Maternal and Newborn Health Program on Postpartum | 2015 | Journal article | ✓ | | | | |

| Chronological reference list: Assessments included in the CBPHC review | | | | | | | |
|---|---|---|---|---|---|---|---|
| | | | Type of assessment carried out | | | | |
| | | | | Child health | | | |
| Reference | Year | Reference type | Mater-nal health | Neo-natal health | Health of children 1-59m | Health of 0-59-m-old children | Equity effect |
| Contraceptive Use and Optimal Birth Spacing in Rural Bangladesh. Stud Fam Plann, 46(3), 297-312. doi:10.1111/j.1728-4465.2015.00031.x | | | | | | | |
| Ahmed, S., Kim, M. H., Dave, A. C., Sabelli, R., Kanjelo, K., Preidis, G. A., . . . Abrams, E. J. (2015). Improved identification and enrolment into care of HIV-exposed and -infected infants and children following a community health worker intervention in Lilongwe, Malawi. J Int AIDS Soc, 18(1), 19305. doi:10.7448/IAS.18.1.19305 | 2015 | Journal article | | | ✓ | | |
| Awor, P., Wamani, H., Tylleskar, T., & Peterson, S. (2015). Drug seller adherence to clinical protocols with integrated management of malaria, pneumonia and diarrhoea at drug shops in Uganda. Malar J, 14, 277. doi:10.1186/s12936-015-0798-9 | 2015 | Journal article | | | | ✓ | |
| Curamericas/Guatemala (2015). Community-Based, Impact-Oriented Child Survial in Huehuetenango, Guatemala: Focused strategic assessment. | 2015 | Report | ✓ | ✓ | | | |
| Ezeanolue, E. E., Obiefune, M. C., Ezeanolue, C. O., Ehiri, J. E., Osuji, A., Ogidi, A. G., . . . Ogedegbe, G. (2015). Effect of a congregation-based intervention on uptake of HIV testing and linkage to care in pregnant women in Nigeria (Baby Shower): a cluster randomised trial. Lancet Glob Health, 3(11), e692-700. doi:10.1016/S2214-109X(15)00195-3 | 2015 | Journal article | ✓ | | | | |
| Fathima, F. N., Raju, M., Varadharajan, K. S., Krishnamurthy, A., Ananthkumar, S. R., & Mony, P. K. (2015). Assessment of 'accredited social health activists'-a national community health volunteer scheme in Karnataka State, India. J Health Popul Nutr, 33(1), 137-145. | 2015 | Journal article | ✓ | | | ✓ | |
| Findley, S. E., Doctor, H. V., Ashir, G. M., Kana, M. A., Mani, A. S., Green, C., & Afenyadu, G. Y. (2015). Reinvigorating health systems and community-based services to improve maternal health outcomes: case study from northern Nigeria. J Prim Care Community Health, 6(2), 88-99. doi:10.1177/2150131914549383 | 2015 | Journal article | ✓ | | | | |
| Gabida, M., Chemhuru, M., Tshimanga, M., Gombe, N. T., Takundwa, L., & Bangure, D. (2015). Effect of distribution of educational material to mothers on duration and severity of diarrhoea and pneumonia, Midlands Province, Zimbabwe: a cluster randomized controlled trial. Int Breastfeed J, 10, 13. doi:10.1186/s13006-015-0037-6 | 2015 | Journal article | | | | ✓ | |
| Haver, J., Brieger, W., Zoungrana, J., Ansari, N., & Kagoma, J. (2015). Experiences engaging community health workers to provide maternal and newborn health services: implementation of four programs. Int J Gynaecol Obstet, 130 Suppl 2, S32-39. doi:10.1016/j.ijgo.2015.03.006 | 2015 | Journal article | ✓ | | | ✓ | |
| Kimani-Murage, E. W., Norris, S. A., Mutua, M. K., Wekesah, F., Wanjohi, M., Muhia, N., . . . Griffiths, P. L. (2016). Potential effectiveness of Community Health Strategy to promote exclusive breastfeeding in urban poor settings in Nairobi, Kenya: a quasi-experimental | 2015 | Journal article | | ✓ | | | |

| Chronological reference list: Assessments included in the CBPHC review | | | | | | | |
|---|---|---|---|---|---|---|---|
| | | | Type of assessment carried out | | | | |
| | | | | Child health | | | |
| Reference | Year | Reference type | Maternal health | Neonatal health | Health of children 1-59m | Health of 0-59-m-old children | Equity effect |
| study. J Dev Orig Health Dis, 7(2), 172-184. doi:10.1017/S2040174415007941 | | | | | | | |
| King, C., McCollum, E. D., Mankhambo, L., Colbourn, T., Beard, J., Hay Burgess, D. C., . . . Mukanga, D. (2015). Can We Predict Oral Antibiotic Treatment Failure in Children with Fast-Breathing Pneumonia Managed at the Community Level? A Prospective Cohort Study in Malawi. PLoS One, 10(8), e0136839. doi:10.1371/journal.pone.0136839 | 2015 | Journal article | | | | ✓ | |
| Kohli, C., Kishore, J., Sharma, S., & Nayak, H. (2015). Knowledge and practice of Accredited Social Health Activists for maternal healthcare delivery in Delhi. J Family Med Prim Care, 4(3), 359-363. doi:10.4103/2249-4863.161317 | 2015 | Journal article | ✓ | | | | |
| Linn, A. M., Ndiaye, Y., Hennessee, I., Gaye, S., Linn, P., Nordstrom, K., & McLaughlin, M. (2015). Reduction in symptomatic malaria prevalence through proactive community treatment in rural Senegal. Trop Med Int Health, 20(11), 1438-1446. doi:10.1111/tmi.12564 | 2015 | Journal article | | | ✓ | | |
| Lunsford, S. S., Fatta, K., Stover, K. E., & Shrestha, R. (2015). Supporting close-to-community providers through a community health system approach: case examples from Ethiopia and Tanzania. Hum Resour Health, 13, 12. doi:10.1186/s12960-015-0006-6 | 2015 | Journal article | ✓ | | | | |
| Memon, Z. A., Khan, G. N., Soofi, S. B., Baig, I. Y., & Bhutta, Z. A. (2015). Impact of a community-based perinatal and newborn preventive care package on perinatal and neonatal mortality in a remote mountainous district in Northern Pakistan. BMC Pregnancy Childbirth, 15, 106. doi:10.1186/s12884-015-0538-8 | 2015 | Journal article | ✓ | ✓ | | | |
| Mohan, D., Gupta, S., LeFevre, A., Bazant, E., Killewo, J., & Baqui, A. H. (2015). Determinants of postnatal care use at health facilities in rural Tanzania: multilevel analysis of a household survey. BMC Pregnancy Childbirth, 15, 282. doi:10.1186/s12884-015-0717-7 | 2015 | Journal article | ✓ | | | | |
| Mushamiri, I., Luo, C., Iiams-Hauser, C., & Ben Amor, Y. (2015). Evaluation of the impact of a mobile health system on adherence to antenatal and postnatal care and prevention of mother-to-child transmission of HIV programs in Kenya. BMC Public Health, 15, 102. doi:10.1186/s12889-015-1358-5 | 2015 | Journal article | ✓ | | | | |
| Nonyane, B. A., Kc, A., Callaghan-Koru, J. A., Guenther, T., Sitrin, D., Syed, U., . . . Baqui, A. H. (2015). Equity improvements in maternal and newborn care indicators: results from the Bardiya district of Nepal. Health Policy Plan. doi:10.1093/heapol/czv077 | 2015 | Journal article | ✓ | | | | ✓ |
| Orobaton, N., Abegunde, D., Shoretire, K., Abdulazeez, J., Fapohunda, B., Lamiri, G., . . . Osborne-Smith, M. (2015). A Report of At-Scale Distribution of Chlorhexidine Digluconate 7.1% Gel for Newborn Cord Care to 36,404 Newborns in Sokoto State, Nigeria: Initial Lessons Learned. PLoS One, 10(7), e0134040. doi:10.1371/journal.pone.0134040 | 2015 | Journal article | | ✓ | | | |

| Chronological reference list: Assessments included in the CBPHC review | | | | | | | |
|---|---|---|---|---|---|---|---|
| | | | Type of assessment carried out | | | | |
| | | | | Child health | | | |
| Reference | Year | Reference type | Mater-nal health | Neo-natal health | Health of children 1-59m | Health of 0-59-m-old children | Equity effect |
| Rahman, M., Jhohura, F. T., Mistry, S. K., Chowdhury, T. R., Ishaque, T., Shah, R., & Afsana, K. (2015). Assessing Community Based Improved Maternal Neonatal Child Survival (IMNCS) Program in Rural Bangladesh. PLoS One, 10(9), e0136898. doi:10.1371/journal.pone.0136898 | 2015 | Journal article | ✓ | | | ✓ | |
| Sesay, F. F., Hodges, M. H., Kamara, H. I., Turay, M., Wolfe, A., Samba, T. T., . . . Jambai, A. (2015). High coverage of vitamin A supplementation and measles vaccination during an integrated Maternal and Child Health Week in Sierra Leone. Int Health, 7(1), 26-31. doi:10.1093/inthealth/ihu073 | 2015 | Journal article | | | | ✓ | |
| Shaw, B., Amouzou, A., Miller, N. P., Tsui, A. O., Bryce, J., Tafesse, M., & Surkan, P. J. (2015). Determinants of Utilization of Health Extension Workers in the Context of Scale-Up of Integrated Community Case Management of Childhood Illnesses in Ethiopia. Am J Trop Med Hyg, 93(3), 636-647. doi:10.4269/ajtmh.14-0660 | 2015 | Journal article | | | | ✓ | |
| Shrestha, J. R., Manandhar, D. S., Manandhar, S. R., Adhikari, D., Rai, C., Rana, H., . . . Pradhan, A. (2015). Maternal and Neonatal Health Knowledge, Service Quality and Utilization: Findings from a Community Based Quasi-experimental Trial in Arghakhanchi District of Nepal. J Nepal Health Res Counc, 13(29), 78-83. | 2015 | Journal article | ✓ | | | | |
| Sitrin, D., Guenther, T., Waiswa, P., Namutamba, S., Namazzi, G., Sharma, S., . . . Moran, A. (2015). Improving newborn care practices through home visits: lessons from Malawi, Nepal, Bangladesh, and Uganda. Glob Health Action, 8, 23963. doi:10.3402/gha.v8.23963 | 2015 | Journal article | | ✓ | | | |
| Smith, A., Sabido, M., Camey, E., Batres, A., & Casabona, J. (2015). Lessons learned from integrating simultaneous triple point-of-care screening for syphilis, hepatitis B, and HIV in prenatal services through rural outreach teams in Guatemala. Int J Gynaecol Obstet, 130 Suppl 1, S70-72. doi:10.1016/j.ijgo.2015.04.009 | 2015 | Journal article | ✓ | | | | |
| Timsa, L., Marrone, G., Ekirapa, E., & Waiswa, P. (2015). Strategies for helping families prepare for birth: experiences from eastern central Uganda. Glob Health Action, 8, 23969. doi:10.3402/gha.v8.23969 | 2015 | Journal article | ✓ | | | | |
| Tomlinson, M., Rotheram-Borus, M. J., Harwood, J., le Roux, I. M., O'Connor, M., & Worthman, C. (2015). Community health workers can improve child growth of antenatally-depressed, South African mothers: a cluster randomized controlled trial. BMC Psychiatry, 15, 225. doi:10.1186/s12888-015-0606-7 | 2015 | Journal article | | | | ✓ | |
| Uzondu, C. A., Doctor, H. V., Findley, S. E., Afenyadu, G. Y., & Ager, A. (2015). Female health workers at the doorstep: a pilot of community-based maternal, newborn, and child health service delivery in northern Nigeria. Glob Health Sci Pract, 3(1), 97-108. doi:10.9745/GHSP-D-14-00117 | 2015 | Journal article | ✓ | ✓ | | | |

| Chronological reference list: Assessments included in the CBPHC review | | | | | | | |
|---|---|---|---|---|---|---|---|
| | | | Type of assessment carried out | | | | |
| | | | | Child health | | | |
| Reference | Year | Reference type | Mater-nal health | Neo-natal health | Health of children 1-59m | Health of 0-59-m-old children | Equity effect |
| Vogt, F., Ferreyra, C., Bernasconi, A., Ncube, L., Taziwa, F., Marange, W., . . . Becher, H. (2015). Tracing defaulters in HIV prevention of mother-to-child transmission programmes through community health workers: results from a rural setting in Zimbabwe. J Int AIDS Soc, 18, 20022. doi:10.7448/IAS.18.1.20022 | 2015 | Journal article | | ✓ | | | |
| Waiswa, P., Pariyo, G., Kallander, K., Akuze, J., Namazzi, G., Ekirapa-Kiracho, E., . . . Uganda Newborn Study, T. (2015). Effect of the Uganda Newborn Study on care-seeking and care practices: a cluster-randomised controlled trial. Glob Health Action, 8, 24584. doi:10.3402/gha.v8.24584 | 2015 | Journal article | ✓ | ✓ | | | |

**Table S4. Assessments of Child Survival Projects funded by the USAID Child Survival and Grants Health Program included in the review**

| NGO | Country | Year of final evaluation | Included in maternal health database | Included in neonatal and child health database |
|---|---|---|---|---|
| ADRA | Cambodia | 2006 | X | X |
| | Guinea | 2005 | X | X |
| | Nicaragua | 2006 | X | X |
| Africare | Senegal | 2008 | | X |
| | Uganda | 2008 | | X |
| AKF | India | 2008 | | X |
| | Pakistan | 2014 | X | X |
| AME-SADA | Haiti | 2009 | | X |
| AMREF | Kenya | 2010 | | X |
| ARC | Cambodia | 2008 | | X |
| CARE | Ethiopia | 2007 | | X |
| | Kenya | 1999 | | X |
| | Mozambique | 2006 | X | X |
| | Nepal | 2011 | | X |
| | Nicaragua | 2008 | X | X |
| | Sierra Leone | 2008 | X | X |
| | Zambia | 2012 | | X |
| Concern Worldwide | Bangladesh | 2008 | | X |
| | Burundi | 2013 | X | X |
| | Haiti | 2010 | | X |
| | Rwanda | 2011 | | X |
| CRS | DR Congo | 2010 | | X |
| | Nicaragua | 2012 | | X |
| Curamericas | Bolivia | 2007 | X | X |
| | Guatemala | 2007 | X | X |
| | | 2015 | X | X |
| | Liberia | 2013 | X | X |
| ERD | Uganda | 2013 | | X |
| FFHI | Bolivia | 1995 | | X |
| | Mozambique | 2010 | | X |
| Future Generations | Peru | 2007 | X | X |
| GHA | Haiti | 2010 | | X |
| GOAL | Ethiopia | 2011 | | X |
| HAI | Timor L'Este | 2008 | | X |
| Health Partners | Uganda | 2010 | | X |
| HealthRight | Kenya | 2010 | | X |
| HHF | Haiti | 2009 | | X |
| HKI | Mali | 2009 | | X |
| | Nepal | 2012 | | X |

| NGO | Country | Year of final evaluation | Included in maternal health database | Included in neonatal and child health database |
|---|---|---|---|---|
| | Niger | 2009 | | X |
| Hope Worldwide | India | 2010 | | X |
| IEF | Malawi | 1995 | | X |
| IRC | Sierra Leone | 2008 | | X |
| IRD | Cambodia | 2010 | | X |
| MCDI | Madagascar | 2015 | | X |
| Mercy Corps | Indonesia | 2010 | | X |
| Mercy Corps | Tajikistan | 2008 | | X |
| MIHV | Uganda | 2004 | | X |
| MTI | Liberia | 2008 | | X |
| Plan | Cameroon | 2004 | | X |
| | Ecuador | 2003 | | X |
| | Kenya | 2009 | | X |
| | Mali | 2006 | | X |
| | Nepal | 2006 | X | X |
| PCI | India | 1999 | | X |
| | Indonesia | 1995 | | X |
| PSI | Malawi | 2011 | | X |
| Relief International | Niger | 2012 | | X |
| Save the Children | Afghanistan | 2008 | | X |
| | Bangladesh | 1995 | | X |
| | Bolivia | 2008 | | X |
| | Ethiopia | 2006 | | X |
| | Guinea | 2006 | | X |
| | Haiti | 1995 | | X |
| | Malawi | 2011 | | X |
| | Mali | 2009 | | X |
| | Zambia | 2014 | X | X |
| World Relief | Bangladesh | 2010 | | X |
| | Burundi | 2012 | | X |
| | Cambodia | 2007 | | X |
| | Malawi | 2009 | | X |
| | Mozambique | 2003 | | X |
| | Mozambique | 2009 | | X |
| | Rwanda | 2006 | | X |
| World Renew | India | 2013 | | X |
| World Vision | Afghanistan | 2013 | | X |
| | India | 1995 | | X |
| | India | 2008 | | X |
| | Nigeria | 1995 | | X |
| | Senegal | 1995 | X | X |

| NGO | Country | Year of final evaluation | Included in maternal health database | Included in neonatal and child health database |
|---|---|---|---|---|
| **Total** | | | 16 | 80 |

Abbreviations:

Adventist Development and Relief Agency

AKF: Aga Khan Foundation

AME-SADA: African Methodist Episcopal Service and Development Agency

AMREF: African Medical and Research Foundation

ARC: American Red Cross

CRS: Catholic Relief Services

ERD: Episcopal Relief and Development

FFHI: Food for the Hungry International

GHA: Global Health Alliance

IEF: International Eye Foundation

HAI: Health Alliance International

HHF: Haitian Health Foundation

HKI: Helen Keller International

IRC: International Rescue Committee

IRD: International Relief and Development

MCDI: Medical Care Development International

MIHV: Minnesota International Health Volunteers (now WellShare)

MTI: Medical Teams International

PCI: Project Concern International

PSI: Population Services International

**Table S5: Maternal Health Data Extraction Form**

# Maternal Health

# Data Extraction Form for Reviewers

## Systematic Review of the Effectiveness of Community-based Primary Health Care

**Working Group on Community-Based Primary Health Care**
**International Health Section**
**American Public Health Association, in association with**
**Maternal and Child Survival Program (MCSP/Jhpiego/USAID)**
**and**
**Department of International Health,**
**Johns Hopkins Bloomberg School of Public Health**

**24 October 2015**

***DATA EXTRACTION FORM***

## ***Instructions***

Please read the article or document carefully from cover to cover and make notes as appropriate. Then answer the questions as best you can. Your answers should be typed electronically using Microsoft Word and this version of the questionnaire. When you finish, please email it to Henry Perry (hperry2@jhu.edu).
Each article will be reviewed independently by two reviewers, and a third reviewer will reconcile any differences encountered in the two reviews and submit the final consolidated review for analysis.

## ***Identifying Information***

**1. List the complete reference** (*authors, title, location of document*)

________________________________________________________________________________
________________________________________________________________________________
________________________________________________________________________________
________________________________________________________________________________

**2. What type of document is this?**

- **a. Scientific article in peer-reviewed journal** [ ]
- **b. Other journal publication** [ ]
- **c. Unpublished project document (including evaluation)** [ ]
- **d. Review article** [ ]
- **e. Other (describe)**________________________________________________

## ***Level and Location of Project/Program/Study***

**3. At what level is this project/program/study?**

- **a. Single community** [ ]
- **b. Set of communities** [ ]
- **c. Sub-province (or district)** [ ]
- **d. Province** [ ]
- **e. National** [ ]
- **f. Multinational** [ ]
- **g. Policy** [ ]
- **h. Other (describe)**________________________________________________
- **i. Unknown** [ ]

**4. Country(ies)** ________________________________________________

**5. If the project/program/study is within one country, indicate the name of the specific location of the study.**

________________________________________________________________________________

**6. Type of geographical area** (*check all that apply*)

- **a. Urban** [ ]
- **b. Peri-urban** [ ]
- **c. Rural** [ ]
- **d. Tropical lowlands** [ ]
- **e. Tropical mountainous** [ ]
- **f. Mountainous desert** [ ]
- **g. Non-Tropical area** [ ]
- **h. Other (specify)**________________________________________________
- **i. Unknown** [ ]

| ***DATA EXTRACTION FORM*** |
| --- |

***Study Interventions*** *(check any that apply)*

**7. a. Is there any other ongoing intervention or program that this specific report is a part of?**

**Yes** [ ]
**No** [ ]

**b. If "yes", please describe it:**

________________________________________________________________________
________________________________________________________________________
________________________________________________________________________
________________________________________________________________________

**c. Is this project/program/study a part of a larger project/program/study? If so, please describe the larger effort.** *(Use the paper's own words if possible)*

________________________________________________________________________
________________________________________________________________________
________________________________________________________________________
________________________________________________________________________

***Note: All subsequent questions will refer to the specific project/program/study which the paper you are reviewing refers to and not any other broader ongoing assessments at the study site.)***

**8. What was the objective of the project/program/study?** *(Use the paper's own words if possible)*

________________________________________________________________________
________________________________________________________________________
________________________________________________________________________
________________________________________________________________________

**9. Maternal health topic area** *(Mark 'X' for Yes; leave blank for No)*

**a. Antenatal care** [ ]
**b. Screening for high-risk pregnancies** [ ]
**c. Gestational diabetes screening/monitoring** [ ]
**d. Post-partum care/home visit for post-partum care** [ ]
**e. Detection/treatment of complications of pregnancy (infection, depression, obstetric fistula, etc.)** [ ]
**f. Monitoring/control of blood pressure (and hypertension)** [ ]
**g. Detection/treatment of infection/sepsis** [ ]
**h. Detection of obstetrical complications** [ ]
**i. Development of birthing plan** [ ]
**j. Promotion of or utilization of trained attendant at delivery** [ ]
**k. Safe delivery kits/assistance with safe delivery** [ ]
**l. Training/support and use of traditional birth attendants** [ ]
**m. Promotion of facility-based delivery** [ ]
**n. Abortion and post-abortion care** [ ]
**o. Prevention/detection/treatment of anemia** [ ]
**p. Prevention/detection/treatment of hemorrhage** [ ]
**q. Immediate breastfeeding post-partum** [ ]
**r. Prevention/detection/treatment of pre-eclampsia or eclampsia** [ ]
**s. Referral for care of obstetrical complications** [ ]
**t. Maternal weight/height/nutritional status** [ ]
**u. Micronutrients** [ ]
**v. Malaria prevention** [ ]
**w. Malaria treatment** [ ]
**x. HIV prevention** [ ]

**y. HIV/AIDS treatment [ ]**
**z. Sexually transmitted disease/reproductive tract infection prevention [ ]**
**aa. Sexually transmitted disease/reproductive tract infection treatment [ ]**
**bb. Tetanus prevention [ ]**
**cc. Tetanus treatment [ ]**
**dd. Syphilis prevention [ ]**
**ee. Syphilis treatment [ ]**
**ff. Immunizations [ ]**
**gg. Family planning (including immediate post-partum contraception) [ ]**
**hh. Primary health care [ ]**
**ii. Women's empowerment [ ]**
**jj. Participatory women's groups [ ]**
**kk. Micro-credit/savings groups [ ]**
**ll. Gender violence [ ]**
**mm. Mental health [ ]**
**nn. Conditional cash transfers [ ]**
**oo Other [ ]**
**(specify)**________________________________________
**pp. Unknown [ ]**

**10a. Interventions** *(one per line for first 4)*
**a. first (major one)**________________________________________
**b. second** ________________________________________
**c. third**________________________________________
**d. fourth**________________________________________
**e. all others** ________________________________________

**10b. Is there any additional important information about the interventions?**

________________________________________________
________________________________________________
________________________________________________
________________________________________________

## ***Project/Program/Study Implementers***

**11. Implementers of the intervention at the community level**

**a. Type of main/direct implementers of the intervention(s)**

1. **Local community members only (who are not trained to be community health workers) [ ]**
2. **Community health workers (both paid and volunteer) [ ]**
3. **Research workers only for this project/program/study [ ]**
4. **Local government health professionals not selected by community [ ]**
5. **Expatriates [ ]**
6. **Other (specify)**____________________________________

**b. Name of implementers:** *(list names of the categories of people implementing the interventions which this study assesses, such as "auxiliary nurses" or "volunteer community health workers called Health Agents")*

________________________________________________
________________________________________________
________________________________________________

**c. Other implementers at community level** (*list any other implementers at the community level)*

____________________________________________________________________________
____________________________________________________________________________
____________________________________________________________________________
____________________________________________________________________________

**12. a. Type of facilitating and/or stakeholding organization(s)** (*entities which cooperated or which had a stake in the intervention or program but were not actual implementers*)

1. **Community only** [ ]
2. **Local NGO** [ ]
3. **Local government** [ ]
4. **State or national government** [ ]
5. **International NGO** [ ]
6. **National NGO** [ ]
7. **Faith-based organization** [ ]
8. **Profit or not-for-profit organization or foundation** [ ]

**b. Name of facilitating organization(s) and any other information:**

____________________________________________________________________________
____________________________________________________________________________
____________________________________________________________________________
____________________________________________________________________________

**13. Did any donor organization(s) support the study?** (*We consider a donor organization to be one which has only a funding role with a limited advisory or review role.*)

1. **USAID** [ ]
2. **AusAid** [ ]
3. **DFID** [ ]
4. **CIDA** [ ]
5. **JICA** [ ]
6. **World Bank** [ ]
7. **WHO** [ ]
8. **UNICEF** [ ]
9. **Other UN Agency (specify)**______________________________________________________
10. **Private company (specify)**______________________________________________________
11. **Other (specify)**__________________________________________________________________

## ***Study Details***

**14. a. Study start year and month (MM/YYYY)** ________/__________

**b. Study end year and month (MM/YYYY)**_________/__________

**15. Please indicate the length of time during which the study took place**

| | | |
|---|---|---|
| **Longer than 10 years** | 4 | [ ] |
| **5 - 9.9 years** | 3 | [ ] |
| **1.0 - 4.9 years** | 2 | [ ] |
| **Less than 1.0 year** | 1 | [ ] |
| **Not applicable** | 9 | [ ] |

## *DATA EXTRACTION FORM*

**16. Design** (*type of study*)

**1. Randomized controlled intervention** [ ]
**2. Non-randomized controlled intervention** [ ]
**3. Uncontrolled intervention** [ ]
**4. Case-control cross-sectional study** [ ]
**5. Cross-sectional study** [ ]
**6. Descriptive study** [ ]
**7. Non-study activity (specify)** [ ] ________________

**Study population** (*describe by grouping, e.g., villages, households or persons, and number of each*)

**17. Intervention group**_______________________________________________________________

**18.a. Was there a control group?** yes__/no__

**b. If Yes, please list:** ______________________________________________________________

**19. If control group present, please indicate:**

**a. Were the intervention and control groups/areas selected at random?** yes__/no__
**b. Were there community and facility-based arms in the intervention and control groups?** yes__/no__
**c. Were there baseline comparisons of the intervention and control groups/areas** yes__/no__
**d. Were there notable differences in socio-demographic characteristics or in other indicators of interest between the intervention and control groups/control areas at baseline?** yes__/no__

**If yes, please explain further:**_______________________________________________________
_____________________________________________________________________________

**20. If no control group, was there any other reference or comparison group used?** yes__/no__
**If yes, please explain further:**_______________________________________________________
_____________________________________________________________________________

**21. Age range of women in the study**

_______________________________________________________

**22. Any special characteristics of the study population?** (e.g., *in terms of sex, socio-economic status, characteristics of the children's mothers*)

_____________________________________________________________________________
_____________________________________________________________________________
_____________________________________________________________________________
_____________________________________________________________________________

**23. Other relevant characteristics of study population** (*e.g., ethnic, cultural, occupational, etc.*)

_____________________________________________________________________________

**24. Were men in the community involved in any way (e.g., fathers, husbands, community leaders, traditional healers)?** yes__/no__ /not specified___

Explain further:

______________________________________________________________________________
______________________________________________________________________________
______________________________________________________________________________
______________________________________________________________________________

## ***Project/Program/Study Context***

**25. What additional information about the context in which the intervention was implemented (aside from the study population characteristics mentioned in Questions 22-23) is significant?** (*e.g., epidemiological situation, status of the health services, long-term presence of research organization, etc.*)

______________________________________________________________________________
______________________________________________________________________________
______________________________________________________________________________
______________________________________________________________________________

**26. What "system" inputs were provided by the project/program/study for implementers at the community level?** (*e.g., training in health care or promotion skills, managerial support, logistics, referral, supervision, and monitoring*)

______________________________________________________________________________
______________________________________________________________________________
______________________________________________________________________________
______________________________________________________________________________

## ***Study Outcomes*** (*refer back to Questions 7-9*)

**27. a. Outcome variables** (*one per line*)

**1. Major outcome** ____________________________________________________________
**2. Second outcome**____________________________________________________________
**3. Third outcome**_____________________________________________________________
**4. Other outcomes**____________________________________________________________

**b. Table of outcome variables and significance level**

| **Outcome** | **Result (check one)** | | **By ___%** | **What was the level of statistical significance?** |
|---|---|---|---|---|
| e.g., bednet coverage | increased _**X**_ | decreased __ | 27% | <0.001 |
| 1. | increased __ | decreased __ | | |
| 2. | increased __ | decreased __ | | |
| 3. | increased __ | decreased __ | | |
| 4. | increased __ | decreased __ | | |

**28. Comment on outcomes**

______________________________________________________________________________
______________________________________________________________________________
______________________________________________________________________________
______________________________________________________________________________
______________________________________________________________________________
______________________________________________________________________________

## *DATA EXTRACTION FORM*

**29. Other key findings not mentioned in your responses to Questions 27 and 28, including unanticipated findings: include details of changes in outcomes, statistical details, and key conclusions.** (*This question is intended to emphasize findings not clearly intended from the interventions mentioned in Questions* 7-9.)

| | | |
|---|---|---|
| **a.** | **Evidence of specific positive health effect(s)** | **[ ]** |
| **b.** | **No useful evidence of any effect** | **[ ]** |
| **c.** | **Evidence of specific negative health effect(s)** | **[ ]** |
| **d.** | **Evidence of effects worth noting other than the intended effect(s)** | **[ ]** |

**Additional comments** (*Make it explicit if you are highlighting findings that the authors themselves have not emphasized. List each finding separately.)*

______________________________________________________________________________
______________________________________________________________________________
______________________________________________________________________________
______________________________________________________________________________
______________________________________________________________________________

## *DATA EXTRACTION FORM*

**30. Which of the following activities/processes/outcomes were included as part of the study?**

**Activities**

| | | |
|---|---|---|
| **a.** | **Micro-credit** | yes__/no__ |
| **b.** | **Income generation** | yes__/no__ |
| **c.** | **Conditional cash transfer** | yes__/no__ |
| **d.** | **Participatory rural appraisal** | yes__/no__ |
| **e.** | **Training of community health workers** | yes__/no__ |
| **f.** | **Formation and/or support of community groups** | yes__/no__ |

**Processes**

| | | |
|---|---|---|
| **a.** | **Promotion of equity** | yes__/no__ |
| **b.** | **Promotion of leadership within the community** | yes__/no__ |
| **c.** | **Promotion of partnerships between the community and the health program** | yes__/no__ |
| **d.** | **Promotion of systems for adaptive learning** | yes__/no__ |
| **e.** | **Promotion of the use of local resources for program support** | yes__/no__ |
| **f,** | **Promotion of community empowerment** | yes__/no__ |
| **g.** | **Promotion of women's empowerment** | yes__/no__ |

**Outcomes**

| | | |
|---|---|---|
| **a.** | **Assessment of sustainability** | yes__/no__ |
| **b.** | **Assessment of community empowerment** | yes__/no__ |
| **c1.** | **Assessment of women's empowerment** | yes__/no__ |
| **c2.** | **Assessment of equity** | yes__/no__ |
| **d.** | **Assessment of cost-effectiveness** | yes__/no__ |
| **e.** | **Assessment of scalability (ease of scaling up to a larger population)** | yes__/no__ |

**31. Provide any additional details about the interventions that may be relevant** (*e.g., coverage, equity, quality, and quantity that were shown to be relevant to the outcomes observed*)

______________________________________________________________________________
______________________________________________________________________________
______________________________________________________________________________
______________________________________________________________________________

**32. Describe any evidence about cost effectiveness:**

______________________________________________________________________________
______________________________________________________________________________
______________________________________________________________________________
______________________________________________________________________________

**33. Describe any social empowerment processes underway in the context of the project/program/study that might explain some of the findings or contribute to the findings:** (*Include here a brief outline of specific empowerment processes that were planned whose presence you have noted in your summary answers in Question 30-Outcomes b and c. If necessary, go back and review or modify your responses. The processes we are trying to identify here include empowerment of individual local community members, of the community as a whole, or of health staff. Forms of empowerment include, but are not limited to, education and training as well as transfer of roles, skills and responsibilities. Were these processes deliberately encouraged by the project/program/study or incidental to it?*)

______________________________________________

______________________________________________

______________________________________________

______________________________________________

______________________________________________

______________________________________________

______________________________________________

**34. a. Did the project/program/study gather and use data for monitoring and evaluation?** yes__/no__

**b. If "yes", describe how:**

______________________________________________

______________________________________________

______________________________________________

______________________________________________

## *Factors Other than Technical Design of Project/Program/Study Influencing Outcomes Studied*

**35. Level of community involvement (put an "X" in the appropriate box for planning, for implementation, and for evaluation)**

| | Level of community involvement | | | | |
|---|---|---|---|---|---|
| **Role of the community in:** | **Highly involved** | **Moderately involved** | **Minimally involved** | **No involvement** | **Information inadequate to make judgment** |
| Planning | | | | | |
| Implementation | | | | | |
| Evaluation | | | | | |

**36. Describe the role of the community in the project/program/study. Was this intended by the project/study? What did the project/study do to realize this role for the community?** (*Here, give specific details regarding the involvement summarized in Question 35*)

______________________________________________

______________________________________________

______________________________________________

______________________________________________

**37. Do you have any additional observations regarding the level of community involvement?** (*e.g., source of evidence, any uncertainties regarding level of community involvement*)

______________________________________________

______________________________________________

______________________________________________

______________________________________________

## *DATA EXTRACTION FORM*

**38. a. Are there any other influences which might have affected the success or failure of the project/program/study?** (*Mark "yes" if you assess that this attribute was present and influenced the outcome of the study*)

| | | Yes | No |
|---|---|---|---|
| **1.** | **Commitments to equity and collaboration** | [ ] | [ ] |
| **2.** | **Presence of strong local leadership** | [ ] | [ ] |
| **3.** | **Partnerships between community, policy makers, technical experts** | [ ] | [ ] |
| **4.** | **Systems for local adaptive learning** | [ ] | [ ] |
| **5.** | **Local resources** | [ ] | [ ] |
| **6.** | **Micro-credit** | [ ] | [ ] |
| **7.** | **Income generation** | [ ] | [ ] |
| **8.** | **Sustainability** | [ ] | [ ] |
| **9.** | **Interdependence** | [ ] | [ ] |
| **10.** | **Holistic and iterative action** | [ ] | [ ] |
| **11.** | **Political or social factors, such as a recent change in government** | [ ] | [ ] |
| **12.** | **Recent or current popular mobilizations** | [ ] | [ ] |

**13. Other (specify)**________________________________________________

**b. Further description if necessary:**

________________________________________________________________
________________________________________________________________
________________________________________________________________
________________________________________________________________

**39. a. Are there any substantial biases or other important limitations which are apparent to you?**
yes__/no__

**b. If "yes", please describe:**

________________________________________________________________
________________________________________________________________
________________________________________________________________
________________________________________________________________
________________________________________________________________

**40. What are the conditions and contextual factors which have contributed to the effectiveness (or ineffectiveness) of the interventions?** *(We have asked previously about which conditions or contextual factors were present. Here, we are asking you to indicate which of these had an important influence on the study's outcomes. Consider here information about such things as appropriate and timely referrals to the health system, identification of high-risk pregnant women -- including recognition of severe illness and danger signs, equity, gender, cost, coverage, and sustainability.).*

________________________________________________________________
________________________________________________________________
________________________________________________________________
________________________________________________________________
________________________________________________________________

| ***DATA EXTRACTION FORM*** |
|---|

**41. a. Did this study show that community participation had an effect on health outcome?** (*This question asks you to make a judgment on the presence of findings on community participation that you included previously in Questions 36 and 37*)

yes__/no__

**b. If "yes", was it positive or negative? Positive__ / negative___**

**c. Describe the specific role community participation played (or did not play) in reaching the observed health outcome?** (*If information not provided, indicate this.*)

______________________________________________
______________________________________________
______________________________________________
______________________________________________
______________________________________________

**42. a. Did this study show that linkages between the health system and the community had an effect on health outcome?** yes__/no__

**b. If "yes", was it positive or negative? Positive__ / negative ___**

**c. Describe the specific linkages and the role they played (or did not play) in reaching the observed health outcome?** (*If information not provided, indicate this.*)

______________________________________________
______________________________________________
______________________________________________
______________________________________________
______________________________________________

## *Reviewer's Assessment of Study Quality*

**43. Please provide your assessment of the quality of the study based on the following** *(If this question does not seem applicable to your study type, check NA):*

**43a. Methodology**

**a. Was there an appropriate control group?** yes__/no__/NA__
**b. Was the sample size adequate to detect a meaningful difference in outcome?** yes__/no__/NA__
**c. Was the data collection appropriate?** yes__/no__/NA__
**d. Are there any substantial biases present?** yes__/no__/NA__
**e. Does the analysis of the data appear appropriate?** yes__/no__/NA__
**f. Overall, does the methodology appear sound?** yes__/no__/NA__

**43b. Please indicate which of the following characteristics the study had. Each characteristic is worth 1 point. On the right-hand column please put the total number of points (8 total).**

| Cohort Study | Control or comparison group | Pre/post intervention data | Random assignment of participants to the intervention | Random selection of participants for assessment | Follow-up rate of 80% or more | Comparison groups equivalent on socio-demographics | Comparison groups equivalent at baseline on outcome measure | Final score (out of 8) |
|---|---|---|---|---|---|---|---|---|
| | | | | | | | | |

**43c. Please add any additional comments that seem appropriate.**

________________________________________
________________________________________
________________________________________

**44. Please summarize here the deficiencies and the strengths related to the quality of the study.**

**Strengths:**________________________________________
________________________________________
________________________________________

**Weaknesses:**________________________________________
________________________________________
________________________________________

**45. What is your overall assessment of the quality of the study?**

________________________________________
________________________________________
________________________________________
________________________________________
________________________________________

**46. Please rate the overall quality of the study**

| | | |
|---|---|---|
| **Exceptional quality** | 5 | [ ] |
| **High quality** | 4 | [ ] |
| **Good quality** | 3 | [ ] |
| **Poor quality** | 2 | [ ] |
| **Unacceptable quality** | 1 | [ ] |
| **Not applicable** | 9 | [ ] |

**47. Approximately how many people were included in the study?**

| | | |
|---|---|---|
| **Number of women in study > 100,000** | 4 | [ ] |
| **Number of women in study 25,000 - 99,999** | 3 | [ ] |
| **Number of women in study 5,000 - 24,999** | 2 | [ ] |
| **Number of women in study < 5,000** | 1 | [ ] |
| **Not applicable** | 9 | [ ] |

## ***Reviewer Identification***

**48. Reviewer** (*type your first and last name*):________________________________________

**49. Date of review (DD/MM/YYYY)** ________/________/________

**50. How much time did you spend reading the document and filling out the questionnaire?**

________________

Please email this form to Dr. Henry Perry (hperry2@jhu.edu).

Thank you for your assistance!

**Table S6. Neonatal and Child Health Data Extraction Form**

# Data Extraction Form for Reviewers

## Systematic Review of the Effectiveness of Community-based Primary Health Care

Working Group on Community-Based Primary Health Care
International Health Section
American Public Health Association, in association with
Maternal and Child Survival Program (MCSP/Jhpiego/USAID)
and
Department of International Health,
Johns Hopkins Bloomberg School of Public Health

24 October 2013

## *DATA EXTRACTION FORM*

## *Instructions*

Please read the article or document carefully from cover to cover and make notes as appropriate. Then answer the questions as best you can. Your answers should be typed electronically using Microsoft Word and this version of the questionnaire. When you finish, please email it to Henry Perry (hperry2@jhu.edu). Each article will be reviewed independently by two reviewers, and another person will reconcile any differences encountered in the two reviews and submit the final consolidated review for analysis.

## *Identifying Information*

**1. List the complete reference** (*authors, title, location of document*)

________________________________________________________________
________________________________________________________________
________________________________________________________________
________________________________________________________________

**2. What type of document is this?**

- **a. Scientific article in peer-reviewed journal** [ ]
- **b. Other journal publication** [ ]
- **c. Unpublished project document (including evaluation)** [ ]
- **d. Review article** [ ]
- **e. Other (describe)**________________________________________

## *Level and Location of Project/Program/Study*

**3. At what level is this project/program/study?**

- **a. Single community** [ ]
- **b. Set of communities** [ ]
- **c. Sub-province (or district)** [ ]
- **d. Province** [ ]
- **e. National** [ ]
- **f. Multinational** [ ]
- **g. Policy** [ ]
- **h. Other (describe)**________________________________________
- **i. Unknown** [ ]

**4. Country(ies)** ________________________________________________

**5. If the project/program/study is within one country, indicate the name of the specific location of the study**

________________________________________________________________

**6. Type of geographical area** (*check all that apply*)

- **a. Urban** [ ]
- **b. Peri-urban** [ ]
- **c. Rural** [ ]
- **d. Tropical lowlands** [ ]
- **e. Tropical mountainous** [ ]
- **f. Mountainous desert** [ ]
- **g. Non-Tropical area** [ ]
- **h. Other (specify)**________________________________________
- **i. Unknown** [ ]

***Study Interventions*** *(check any that apply)*

**7. a. Is there any other ongoing intervention or program that this specific report is a part of?**

**Yes** [ ]

**No** [ ]

**b. If "yes", please describe it:**

______________________________________________________________________
______________________________________________________________________
______________________________________________________________________
______________________________________________________________________

**c. Is this project/program/study a part of a larger project/program/study? If so, please describe the larger effort.** *(Use the paper's own words if possible)*

______________________________________________________________________
______________________________________________________________________
______________________________________________________________________
______________________________________________________________________

***Note: All subsequent questions will refer to the specific project/program/study which the paper you are reviewing refers to and not any other broader ongoing assessments at the study site.)***

**8. What was the objective of the project/program/study?** *(Use the paper's own words if possible)*

______________________________________________________________________
______________________________________________________________________
______________________________________________________________________
______________________________________________________________________

**9. Child health topic area** *(Mark 'X' for Yes; leave blank for No)*

**a. Child weight/height** [ ]
**b. Breastfeeding** [ ]
**c. Complementary feeding** [ ]
**d. Micronutrients** [ ]
**e. Diarrhea prevention** [ ]
**f. Diarrhea treatment** [ ]
**g. Pneumonia prevention** [ ]
**h. Pneumonia treatment** [ ]
**i. Malaria prevention** [ ]
**j. Malaria treatment** [ ]
**k. HIV prevention** [ ]
**l. HIV/AIDS treatment** [ ]
**m. Neonatal tetanus prevention** [ ]
**n. Neonatal tetanus treatment** [ ]
**o. Measles prevention** [ ]
**p. Measles treatment** [ ]
**q. Congenital syphilis prevention** [ ]
**r. Congenital syphilis treatment** [ ]
**s. Immunizations** [ ]
**t. Neonatal/perinatal health** [ ]
**u. Childhood illness/IMCI (integrated management of childhood illness)** [ ]
**v. Primary health care** [ ]
**w. Other (specify)**______________________________________________
**x. Unknown** [ ]

**10a. Interventions** *(one per line for first 4)*

**a. first (major one)**______________________________

**b. second** ______________________________

**c. third**______________________________

**d. fourth**______________________________

**e. all others** ______________________________

**10b. Is there any additional important information about the interventions?**

______________________________
______________________________
______________________________
______________________________

## ***Project/Program/Study Implementers***

**11. Implementers of the intervention at the community level**

**a. Type of main/direct implementers of the intervention(s)**

1. **Local community members only (who are not trained to be community health workers)** [ ]
2. **Community health workers (both paid and volunteer)** [ ]
3. **Research workers only for this project/program/study** [ ]
4. **Local government health professionals not selected by community** [ ]
5. **Expatriates** [ ]
6. **Other (specify)**______________________________

**b. Name of implementers:** *(list names of the categories of people implementing the interventions which this study assesses, such as "auxiliary nurses" or "volunteer community health workers called Health Agents")*

______________________________
______________________________
______________________________

**c. Other implementers at community level** *(list any other implementers at the community level)*

______________________________
______________________________
______________________________
______________________________

**12. a. Type of facilitating and/or stakeholding organization(s)** *(entities which cooperated or which had a stake in the intervention or program but were not actual implementers)*

1. **Community only** [ ]
2. **Local NGO** [ ]
3. **Local government** [ ]
4. **State or national government** [ ]
5. **International NGO** [ ]
6. **National NGO** [ ]
7. **Faith-based organization** [ ]
8. **Profit or not-for-profit organization or foundation** [ ]

**b. Name of facilitating organization(s) and any other information:**

_______________________________________________________________________________
_______________________________________________________________________________
_______________________________________________________________________________
_______________________________________________________________________________

**13. Did any donor organization(s) support the study?** (*We consider a donor organization to be one which has only a funding role with a limited advisory or review role.*)

1. **USAID** [ ]
2. **AusAid** [ ]
3. **DFID** [ ]
4. **CIDA** [ ]
5. **JICA** [ ]
6. **World Bank** [ ]
7. **WHO** [ ]
8. **UNICEF** [ ]
9. **Other UN Agency (specify)**______________________________________________
10. **Private company (specify)**______________________________________________
11. **Other (specify)**______________________________________________________

## ***Study Details***

**14. a. Study start year and month (MM/YYYY)** ________/__________

**b. Study end year and month (MM/YYYY)**________/__________

**15. Please indicate the length of time during which the study took place**

| | | |
|---|---|---|
| **Longer than 10 years** | 4 | [ ] |
| **5 - 9.9 years** | 3 | [ ] |
| **1.0 - 4.9 years** | 2 | [ ] |
| **Less than 1.0 year** | 1 | [ ] |
| **Not applicable** | 9 | [ ] |

**16. Design** (*type of study*)

| | |
|---|---|
| **1. Randomized controlled intervention** | [ ] |
| **2. Non-randomized controlled intervention** | [ ] |
| **3. Uncontrolled intervention** | [ ] |
| **4. Case-control cross-sectional study** | [ ] |
| **5. Cross-sectional study** | [ ] |
| **6. Descriptive study** | [ ] |
| **7. Non-study activity (specify)** | [ ] ______________ |

**Study population** (*describe by grouping, e.g., villages, households or persons, and number of each*)

**17. Intervention group**______________________________________________________

**18.a. Was there a control group?** yes__/no__

**b. If Yes, please list:** ___________________________________________________

**19. If control group present, please indicate:**

**a. Were the intervention and control groups/areas selected at random?** yes__/no__
**b. Were there community and facility-based arms in the intervention and control groups?** yes__/no__
**c. Were there baseline comparisons of the intervention and control groups/areas** yes__/no__
**d. Were there notable differences in socio-demographic characteristics or in other indicators of interest between the intervention and control groups/control areas at baseline?** yes__/no__

**If yes, please explain further:**______________________________________________

**20. If no control group, was there any other reference or comparison group used?** yes__/no__
**If yes, please explain further:**______________________________________________

**21. Age range of study children** ______________________________________________

**22. Any special characteristics of the study population?** (e.g., *in terms of sex, socio-economic status, characteristics of the children's mothers*)

**23. Other relevant characteristics of study population** (*e.g., ethnic, cultural, occupational, etc.*)

**24. Were men in the community involved in any way (e.g., fathers, community leaders, traditional healers)?** yes__/no__ /not specified___

Explain further:

## ***Project/Program/Study Context***

**25. What additional information about the context in which the intervention was implemented (aside from the study population characteristics mentioned in Questions 22-23) is significant?** (*e.g., epidemiological situation, status of the health services, long-term presence of research organization, etc.*)

**26. What "system" inputs were provided by the project/program/study for implementers at the community level?** (*e.g., training in health care or promotion skills, managerial support, logistics, referral, supervision, and monitoring*)

___

___

___

___

***Study Outcomes*** (*refer back to Questions 7-9*)

**27. a. Outcome variables** (*one per line*)

**1. Major outcome** ___

**2. Second outcome** ___

**3. Third outcome** ___

**4. Other outcomes** ___

**b. Table of outcome variables and significance level**

| **Outcome** | **Result (check one)** | | **By ___%** | **What was the level of statistical significance?** |
|---|---|---|---|---|
| e.g., bednet coverage | increased _X_ | decreased __ | 27% | <0.001 |
| 1. | increased __ | decreased __ | | |
| 2. | increased __ | decreased __ | | |
| 3. | increased __ | decreased __ | | |
| 4. | increased __ | decreased __ | | |

**28. Comment on outcomes**

___

___

___

___

___

___

**29. Other key findings not mentioned in your responses to Questions 27 and 28, including unanticipated findings: include details of changes in outcomes, statistical details, and key conclusions.** (*This question is intended to emphasize findings not clearly intended from the interventions mentioned in Questions 7-9.*)

a. **Evidence of specific positive health effect(s)** [ ]
b. **No useful evidence of any effect** [ ]
c. **Evidence of specific negative health effect(s)** [ ]
d. **Evidence of effects worth noting other than the intended effect(s)** [ ]

**Additional comments** (*Make it explicit if you are highlighting findings that the authors themselves have not emphasized. List each finding separately.*)

___

___

___

___

___

**30. Which of the following activities/processes/outcomes were included as part of the study?**

**Activities**

| | | |
|---|---|---|
| **a.** | **Micro-credit** | yes__/no__ |
| **b.** | **Income generation** | yes__/no__ |
| **c.** | **Conditional cash transfer** | yes__/no__ |
| **d.** | **Participatory rural appraisal** | yes__/no__ |
| **e.** | **Training of community health workers** | yes__/no__ |
| **f.** | **Formation and/or support of community groups** | yes__/no__ |

**Processes**

| | | |
|---|---|---|
| **a.** | **Promotion of equity** | yes__/no__ |
| **b.** | **Promotion of leadership within the community** | yes__/no__ |
| **c.** | **Promotion of partnerships between the community and the health program** | yes__/no__ |
| **d.** | **Promotion of systems for adaptive learning** | yes__/no__ |
| **e.** | **Promotion of the use of local resources for program support** | yes__/no__ |
| **f,** | **Promotion of community empowerment** | yes__/no__ |
| **g.** | **Promotion of women's empowerment** | yes__/no__ |

**Outcomes**

| | | |
|---|---|---|
| **a.** | **Assessment of sustainability** | yes__/no__ |
| **b.** | **Assessment of community empowerment** | yes__/no__ |
| **c1.** | **Assessment of women's empowerment** | yes__/no__ |
| **c2.** | **Assessment of equity** | yes__/no__ |
| **d.** | **Assessment of cost-effectiveness** | yes__/no__ |
| **e.** | **Assessment of scalability (ease of scaling up to a larger population)** | yes__/no__ |

**31. Provide any additional details about the interventions that may be relevant** (*e.g., coverage, equity, quality, and quantity that were shown to be relevant to the outcomes observed*)

______________________________________________________________________
______________________________________________________________________
______________________________________________________________________
______________________________________________________________________

**32. Describe any evidence about cost effectiveness:**

______________________________________________________________________
______________________________________________________________________
______________________________________________________________________
______________________________________________________________________

**33. Describe any social empowerment processes underway in the context of the project/program/study that might explain some of the findings or contribute to the findings:** (*Include here a brief outline of specific empowerment processes that were planned whose presence you have noted in your summary answers in Question 30-Outcomes b and c. If necessary, go back and review or modify your responses. The processes we are trying to identify here include empowerment of individual local community members, of the community as a whole, or of health staff. Forms of empowerment include, but are not limited to, education and training as well as transfer of roles, skills and responsibilities. Were these processes deliberately encouraged by the project/program/study or incidental to it?*)

______________________________________________________________________________
______________________________________________________________________________
______________________________________________________________________________
______________________________________________________________________________
______________________________________________________________________________
______________________________________________________________________________
______________________________________________________________________________

**34. a. Did the project/program/study gather and use data for monitoring and evaluation?** yes__/no__
**b. If "yes", describe how:**

______________________________________________________________________________
______________________________________________________________________________
______________________________________________________________________________
______________________________________________________________________________

## *Factors Other than Technical Design of Project/Program/Study Influencing Outcomes Studied*

**35. Level of community involvement (put an "X" in the appropriate box for planning, for implementation, and for evaluation)**

| | Level of community involvement | | | | |
|---|---|---|---|---|---|
| **Role of the community in:** | **Highly involved** | **Moderately involved** | **Minimally involved** | **No involvement** | **Information inadequate to make judgment** |
| Planning | | | | | |
| Implementation | | | | | |
| Evaluation | | | | | |

**36. Describe the role of the community in the project/program/study. Was this intended by the project/study? What did the project/study do to realize this role for the community?** (*Here, give specific details regarding the involvement summarized in Question 35*)

______________________________________________________________________________
______________________________________________________________________________
______________________________________________________________________________
______________________________________________________________________________

**37. Do you have any additional observations regarding the level of community involvement?** (*e.g., source of evidence, any uncertainties regarding level of community involvement*)

______________________________________________________________________________
______________________________________________________________________________
______________________________________________________________________________
______________________________________________________________________________

**38. a. Are there any other influences which might have affected the success or failure of the project/program/study?** (*Mark "yes" if you assess that this attribute was present and influenced the outcome of the study*)

| | | Yes | No |
|---|---|---|---|
| **1.** | **Commitments to equity and collaboration** | [ ] | [ ] |
| **2.** | **Presence of strong local leadership** | [ ] | [ ] |
| **3.** | **Partnerships between community, policy makers, technical experts** | [ ] | [ ] |
| **4.** | **Systems for local adaptive learning** | [ ] | [ ] |
| **5.** | **Local resources** | [ ] | [ ] |
| **6.** | **Micro-credit** | [ ] | [ ] |
| **7.** | **Income generation** | [ ] | [ ] |
| **8.** | **Sustainability** | [ ] | [ ] |
| **9.** | **Interdependence** | [ ] | [ ] |
| **10.** | **Holistic and iterative action** | [ ] | [ ] |
| **11.** | **Political or social factors, such as a recent change in government** | [ ] | [ ] |
| **12.** | **Recent or current popular mobilizations** | [ ] | [ ] |
| **13.** | **Other (specify)**________________________________________________ | | |

**b. Further description if necessary:**

____________________________________________________________________________
____________________________________________________________________________
____________________________________________________________________________
____________________________________________________________________________

**39. a. Are there any substantial biases or other important limitations which are apparent to you?**
yes__/no__

**b. If "yes", please describe:**

____________________________________________________________________________
____________________________________________________________________________
____________________________________________________________________________
____________________________________________________________________________
____________________________________________________________________________

**40. What are the conditions and contextual factors which have contributed to the effectiveness (or ineffectiveness) of the interventions?** *(We have asked previously about which conditions or contextual factors were present. Here, we are asking you to indicate which of these had an important influence on the study's outcomes. Consider here information about such things as appropriate and timely referrals to the health system, identification of high-risk children -- including recognition of severe illness and danger signs, equity, gender, cost, coverage, and sustainability.).*

____________________________________________________________________________
____________________________________________________________________________
____________________________________________________________________________
____________________________________________________________________________
____________________________________________________________________________

**41. a. Did this study show that community participation had an effect on health outcome?** (*This question asks you to make a judgment on the presence of findings on community participation that you included previously in Questions 36 and 37*)

yes__/no__

**b. If "yes", was it positive or negative? Positive__ / negative___**

**c. Describe the specific role community participation played (or did not play) in reaching the observed health outcome?** (*If information not provided, indicate this.*)

______________________________________________________________________
______________________________________________________________________
______________________________________________________________________
______________________________________________________________________
______________________________________________________________________

**42. a. Did this study show that linkages between the health system and the community had an effect on health outcome?** yes__/no__

**b. If "yes", was it positive or negative? Positive__ / negative ___**

**c. Describe the specific linkages and the role they played (or did not play) in reaching the observed health outcome?** (*If information not provided, indicate this.*)

______________________________________________________________________
______________________________________________________________________
______________________________________________________________________
______________________________________________________________________
______________________________________________________________________

## *Reviewer's Assessment of Study Quality*

**43. Please provide your assessment of the quality of the study based on the following** *(If this question does not seem applicable to your study type, check NA):*

**<u>43a. Methodology</u>**

**a. Was there an appropriate control group?** yes__/no__/NA__
**b. Was the sample size adequate to detect a meaningful difference in outcome?** yes__/no__/NA__
**c. Was the data collection appropriate?** yes__/no__/NA__
**d. Are there any substantial biases present?** yes__/no__/NA__
**e. Does the analysis of the data appear appropriate?** yes__/no__/NA__
**f. Overall, does the methodology appear sound?** yes__/no__/NA__

**43b. Please indicate which of the following characteristics the study had. Each characteristic is worth 1 point. On the right-hand column please put the total number of points (8 total).**

| Cohort Study | Control or comparison group | Pre/post intervention data | Random assignment of participants to the intervention | Random selection of participants for assessment | Follow-up rate of 80% or more | Comparison groups equivalent on socio-demographics | Comparison groups equivalent at baseline on outcome measure | Final score (out of 8) |
|---|---|---|---|---|---|---|---|---|
| | | | | | | | | |

**43c. Please add any additional comments that seem appropriate.**

____________________________________________________________________________

**44. Please summarize here the deficiencies and the strengths related to the quality of the study.**

Strengths:________________________________________________________________

Weaknesses:______________________________________________________________

**45. What is your overall assessment of the quality of the study?**

____________________________________________________________________________

**46. Please rate the overall quality of the study**

| | | |
|---|---|---|
| Exceptional quality | 5 | [ ] |
| High quality | 4 | [ ] |
| Good quality | 3 | [ ] |
| Poor quality | 2 | [ ] |
| Unacceptable quality | 1 | [ ] |
| Not applicable | 9 | [ ] |

**47. Approximately how many people were included in the study?**

| | | |
|---|---|---|
| Number of children in study > 100,000 | 4 | [ ] |
| Number of children in study 25,000 - 99,999 | 3 | [ ] |
| Number of children in study 5,000 - 24,999 | 2 | [ ] |
| Number of children in study < 5,000 | 1 | [ ] |
| Not applicable | 9 | [ ] |

## *Reviewer Identification*

**48. Reviewer** (*type your first and last name*):________________________________________

**49. Date of review (DD/MM/YYYY)** _______/________/________

**50. How much time did you spend reading the document and filling out the questionnaire?**

______________

Please email this form to Dr. Henry Perry (hperry2@jhu.edu).
Thank you for your assistance!



**Table S7. Reviewers of assessments**

| Last name | First Name | Degrees (at the time of their participation in the review) | Other Information |
|---|---|---|---|
| Adhikari | Binita | MSPH candidate | Department of International Health, Bloomberg School of Public Health, Johns Hopkins University, Baltimore, MD |
| Aftab | Asma | MD, MPH | Assistant Scientist, Department of Family Medicine and Community Health, University of Miami School of Medicine, Miami, FL |
| Ahmadi | Azal | PhD candidate | Department of International Health, Bloomberg School of Public Health, Johns Hopkins University, Baltimore, MD |
| Aitken | Iain | MD, MPH | Principal Program Associate, Center for Health Outcomes, Management Sciences for Health, Cambridge, MA |
| Altobelli | Laura | RN, DrPH | Country Director, Future Generations/Peru, Lima, Peru |
| Anakwenze | Chidinma | MPH candidate | Bloomberg School of Public Health, Johns Hopkins University, Baltimore, MD |
| Asgary | Ramin | MD, MPH, MSc, CTM&H | Assistant Professor, Departments of Internal Medicine and Family & Social Medicine, Montefiore Medical Center, Bronx, NY |
| Hill | Ann Ashworth | PhD | Professor Emeritus of Community Nutrition, London School of Hygiene and Tropical Medicine, London, England |
| Berggren | Gretchen | MD, MPH | International Public Health Consultant, Golden, CO |
| Berggren | Warren | MD, DrPH | International Public Health Consultant, Boulder, CO |
| Boswell | Claire | MPH | International Public Health Consultant, Alexandria, VA |
| Bowen | Lisa | MPH | International Health Consultant, Boone, NC |
| Brown | Amberle | MPH candidate | Bloomberg School of Public Health, Johns Hopkins University, Baltimore, MD |
| Bryant | Jack | MD | Adjunct Professor, Department of International Health, Bloomberg School of Public Health, Johns Hopkins University, Baltimore, MD |
| Cesar | Juraci | MD, PhD | Adjunct Professor, Division of Population & Health, Faculty of Medicine, Universidade Federal do Rio Grande, Rio Grande, RS, Brazil |
| Chan | Elizabeth | MPH candidate | Bloomberg School of Public Health, Johns Hopkins University, Baltimore, MD |
| Chang | Stephanie | MD, MPH | Clinical Research Fellow, Division of General Internal Medicine, Department of Medicine, Johns Hopkins University, Baltimore, MD |
| Cheatham | Elizabeth | MA, MPH | Operations Manager, Konbit Sante, Portland, ME |
| Chitnis | Ketan | PhD | Programme Communication Officer, UNICEF, New York, NY |
| Christie | Len | MD, MPH | Cardiology consultant, Eugene, OR |

| Last name | First Name | Degrees (at the time of their participation in the review) | Other Information |
|---|---|---|---|
| Crouse | Deanna | MPH | Senior Program Manager, Social & Scientific Systems, Inc., Washington, DC |
| Davachi | Christine | MSc | Development Economics, School of Oriental and African Studies, University of London, England |
| Dortonne | Jean Richard | MD | International Public Health Consultant, Montreal, Canada |
| Dowell | Duane | MD | International Pediatric Specialist (retired), Ann Arbor, MI |
| Emami | Ashkan | JD candidate | American University, Washington, DC |
| Enoh | Sheila | MPH candidate | Bloomberg School of Public Health, Johns Hopkins University, Baltimore, MD |
| Fan | Qi | MSPH candidate | Department of International Health, Bloomberg School of Public Health, Johns Hopkins University, Baltimore, MD |
| Fort | Meredith | PhD candidate | University of Washington School of Public Health, Department of International Health, Seattle, WA |
| Freeman | Paul | MD, DrPH, MPH | International Public Health Consultant, Seattle, WA |
| George | Asha | D Phil, MPH | Health Specialist, Community-based Approaches, Policy and Evidence Unit, Health Programme Division, UNICEF, New York, NY |
| Grant | Juliana | MPH | Epidemiologic Intelligence Service, Centers for Disease Control, Atlanta, GA |
| Grau | Stacy | MPH | Child Survival Technical Advisor, World Relief, Maputu, Mozambique |
| Gupta | Sundeep | MD, MPH | CDC Global AIDS Program, Honduras Country Director, Tegucigalpa, Honduras |
| Habarta | Nancy | MPH | Research Fellow, Centers for Disease Control, Atlanta, GA |
| Haq | Nowreen | MD, MPH | Research Program Coordinator, Division of General Internal Medicine, Johns Hopkins Medical Institutions, Baltimore, MD |
| Haq | Runa | MD, MPH | Health consultant and advocate for adolescents and youth, Baltimore, MD |
| Hashemi | Paymon | MPH | Research Assistant, Environmental Epidemiology Service, Department of Veterans Affairs, Washington, DC |
| Hershberger | Ann | RN, PhD | Professor of Nursing, Eastern Mennonite University, Harrisonburg, VA |
| Hill | Zelee | PhD, MSc | Research Fellow, London School of Hygiene and Tropical Medicine, London, England |

| Last name | First Name | Degrees (at the time of their participation in the review) | Other Information |
|---|---|---|---|
| Hoar | Sandy | MPAS, PA-C | Assistant Clinical Professor, Health Sciences Programs and Global Health, George Washington University, Washington, DC |
| Jani | Asim | MD, MPH | Medical Epidemiologist, Coordinating Office for Global Health, Centers for Disease Control and Prevention, Atlanta, GA |
| Kim | Dennis | MHS candidate | Reproductive and Cancer Biology, Johns Hopkins School of Public Health, Baltimore, MD |
| Kim | Woon Cho | MPH candidate | Department of Epidemiology, Rollins School of Public Health, Emory University, Atlanta, GA |
| Kumar | Ajoy | MSc | Vaccine Quality Control, Biological E Pvt Ltd, Hyderabad, India |
| Laswell | Stacy | MPH | ORISE Fellow (Reproductive Health for Refugees), Division of Reproductive Health, Centers for Disease Control and Prevention, Atlanta, GA |
| Llanque | Ramiro | MD, MPH | Program Advisor, Consejo de Salud Rural Andino, La Paz, Bolivia |
| Long | Amanda | MPH candidate | Bloomberg School of Public Health, Johns Hopkins University, Baltimore, MD |
| Mataya | Ron | MD | Chairman and Assistant Professor, Department of Global Health, Loma Linda University School of Public Health, Loma Linda, CA |
| McCord | Colin | MD | International Public Health Specialist, New York, NY |
| McMorrow | Meredith | MD, MPH | Malaria Branch, Division of Parasitic Diseases, Centers for Disease Control and Prevention, Atlanta, GA |
| Menager | Henri | MD, MPH | Epidemiologist, Cancer Prevention and Control Program, Office of Health Promotion, Kansas Department of Health and Environment, Topeka, KS |
| Mendoza-Sassi | Raul | MD, PhD | Adjunct Professor, Division of Population & Health, Faculty of Medicine, Universidade Federal do Rio Grande, Rio Grande, RS, Brazil |
| Menson | William | MD | MPH student, Bloomberg School of Public Health, Johns Hopkins University, Baltimore, MD |
| Metangmo | Pierre-Marie | MD, MA, MPH | Senior Technical Advisor, Africa<br>Leadership, Management & Sustainability Program<br>Management Sciences for Health<br>Cambridge, MA |
| Mirchandani | Gita | PhD, MPH | EIS Fellow, Epidemiology and Disease Control Programs, Maryland Department of Health and Mental Hygiene, Baltimore, MD |
| Morgan | Mary | MPH candidate | Bloomberg School of Public Health, Johns Hopkins University, Baltimore, MD |

| Last name | First Name | Degrees (at the time of their participation in the review) | Other Information |
|---|---|---|---|
| Neat | Lenna | MPH candidate | Bloomberg School of Public Health, Johns Hopkins University, Baltimore, MD |
| Ogundalu | Oluwatosin | MPH candidate | Rollins School of Public Health, Emory University, Atlanta, GA |
| Paredes | Pat | MD, MSc, DrPH | Global Health Consultant, Washington, DC |
| Parekh | Vikash | MHS | Port St. Lucie, FL |
| Passeri | Carlo | MHS candidate | Department of Biochemistry, Bloomberg School of Public Health, Johns Hopkins University, Baltimore, MD |
| Patel | Zohra | PhD candidate | Department of International Health, Bloomberg School of Public Health, Johns Hopkins University, Baltimore, MD |
| Perez | Erika | PhD candidate | Department of International Health, Bloomberg School of Public Health, Johns Hopkins University, Baltimore, MD |
| Perry | Henry | MD, PhD, MPH | Carl Taylor Professor for Equity and Empowerment, Future Generations, Franklin, WV |
| Podewils | Laura | PhD | Epidemiologist, Division of Tuberculosis Elimination, National Center for HIV/AIDS Viral Hepatitis, STD, and TB Prevention, Centers for Disease Control and Prevention, Atlanta, GA |
| Poehlman | Jon | PhD | Health Communication Researcher, RTI International, Research Triangle, NC |
| Ragunanthan | Braveen | MD, MPH candidate | Bloomberg School of Public Health, Johns Hopkins University, Baltimore, MD |
| Premkumar | Ramaswamy | PhD | Schieffelin Leprosy Research & Training Center, Karigiri, Tamil Nadu, India |
| Rassekh | Bahie Mary | MHS, PhD | Public Health Specialist, The World Bank, Washington, DC |
| Ricca | James | MD, MPH | Capacity Development Advisor, Child Survival Technical Support Project, ORC Macro, Calverton, MD |
| Rima | Jeeva | MBBS | Resident in Family Medicine, Apollo Hospitals, Hyderabad, India |
| Rohde | Jon | MD | International Public Health Specialist, Cape Town, South Africa |
| Russell | Evan | medical student | Johns Hopkins University School of Medicine, Baltimore, MD |
| Sacks | Emma | PhD candidate | Department of International Health, Bloomberg School of Public Health, Johns Hopkins University, Baltimore, MD |
| Sakyi | Kwame | PhD candidate | Department of International Health, Bloomberg School of Public Health, Johns Hopkins University, Baltimore, MD |

| Last name | First Name | Degrees (at the time of their participation in the review) | Other Information |
|---|---|---|---|
| Sanchez | Juan | PhD candidate | Department of International Health, Bloomberg School of Public Health, Johns Hopkins University, Baltimore, MD |
| Shah | Nirali | PhD candidate | Department of International Health, Bloomberg School of Public Health, Johns Hopkins University, Baltimore, MD |
| Shankar | Manjunath | PhD candidate | Department of International Health, Bloomberg School of Public Health, Johns Hopkins University, Baltimore, MD |
| Sharan | Mona | PhD | Consultant, The World Bank, Washington, DC |
| Sillan | Donna | MPH | International Public Health Consultant, Mill Valley, CA |
| Stake | Stephen | MHS candidate | MHS Candidate in the Department of International Health (Social and Behavioral Interventions), Bloomberg School of Public Health, Johns Hopkins University, Baltimore, MD |
| Steinhardt | Laura | MPH, PhD candidate | Department of International Health, Bloomberg School of Public Health, Johns Hopkins University, Baltimore, MD |
| Suchdev | Parminder S. | MD, MPH | Epidemic Intelligence Service Officer, Pediatrician, Maternal and Child Nutrition Branch, Centers for Disease Control and Prevention, Atlanta, GA |
| Sylla | Mariame | MD | Health Programme Specialist, Health Programme Division, UNICEF, New York, NY |
| Tamarro | Meghan | MPH candidate | Bloomberg School of Public Health, Johns Hopkins University, Baltimore, MD |
| Taylor | Henry | MD, MPH | Senior Associate in Applied Public Health, Bloomberg School of Public Health, Johns Hopkins University, Baltimore, MD |
| Tegbe | Muyiwa | MD, MPH candidate | Bloomberg School of Public Health, Johns Hopkins University, Baltimore, MD |
| Ti | Angeline | MPH candidate | Bloomberg School of Public Health, Johns Hopkins University, Baltimore, MD |
| Teller | Charles | MA, PhD | Bixby Visiting Scholar, Population Reference Bureau, Washington, DC |
| Tuakli | Yetsa | MD, MPH candidate | Bloomberg School of Public Health, Johns Hopkins University, Baltimore, MD |
| Wilhelm | Jess | PhD candidate | Department of International Health, Bloomberg School of Public Health, Johns Hopkins University, Baltimore, MD |
| Wollinka | Olga | MPH candidate | Bloomberg School of Public Health, Johns Hopkins University, Baltimore, MD |

| Last name | First Name | Degrees (at the time of their participation in the review) | Other Information |
|---|---|---|---|
| Yuan | Jean | MD | Epidemiologic Intelligence Service, Centers for Disease Control (working with the Infectious Disease Branch of the California Department of Health Services, Richmond, CA) |

**Table S8. Location of maternal, neonatal and child health programs, projects and field studies with assessments that have been included in the database by country and region**

| Region/country | Child | Maternal | Total | Percent |
|---|---|---|---|---|
| **Total*** | | | **786** | **100%** |
| **Africa** | **304** | **80** | **384** | **48.9%** |
| Benin | 6 | 2 | 8 | 1.0% |
| Burkina Faso | 11 | 1 | 12 | 1.5% |
| Burundi | 3 | 1 | 4 | 0.5% |
| Cameroon | 6 | 1 | 7 | 0.9% |
| Democratic Republic of the Congo | 11 | 2 | 13 | 1.7% |
| Eritrea | 0 | 2 | 2 | 0.3% |
| Ethiopia | 24 | 4 | 28 | 3.6% |
| Gabon | 1 | 0 | 1 | 0.1% |
| Gambia | 11 | 3 | 14 | 1.8% |
| Ghana | 28 | 8 | 36 | 4.6% |
| Guinea | 5 | 3 | 8 | 1.0% |
| Guinea Bissau | 4 | 0 | 4 | 0.5% |
| Ivory Coast | 1 | 0 | 1 | 0.1% |
| Kenya | 24 | 3 | 27 | 3.4% |
| Lesotho | 1 | 0 | 1 | 0.1% |
| Liberia | 5 | 4 | 9 | 1.1% |
| Madagascar | 8 | 0 | 8 | 1.0% |
| Malawi | 15 | 4 | 19 | 2.4% |
| Mali | 6 | 1 | 7 | 0.9% |
| Mozambique | 5 | 3 | 8 | 1.0% |
| Niger | 5 | 1 | 6 | 0.8% |
| Nigeria | 10 | 7 | 17 | 2.2% |
| Rwanda | 10 | 3 | 13 | 1.7% |
| Senegal | 13 | 4 | 17 | 2.2% |
| Sierra Leone | 6 | 2 | 8 | 1.0% |
| South Africa | 13 | 1 | 14 | 1.8% |
| Sudan (South & North) | 3 | 3 | 6 | 0.8% |
| Tanzania | 24 | 6 | 30 | 3.8% |
| Togo | 1 | 0 | 1 | 0.1% |
| Uganda | 27 | 7 | 34 | 4.3% |

| Region/country | Child | Maternal | Total | Percent |
|---|---|---|---|---|
| Zambia | 10 | 2 | 12 | 1.5% |
| Zimbabwe | 6 | 2 | 8 | 1.0% |
| Multiple African countries (not otherwise stated) | 1 | 0 | 1 | 0.1% |
| **The Americas** | **62** | **14** | **76** | **9.7%** |
| Bolivia | 8 | 2 | 10 | 1.3% |
| Brazil | 11 | 2 | 13 | 1.7% |
| Chile | 0 | 2 | 2 | 0.3% |
| Colombia | 3 | 0 | 3 | 0.4% |
| Dominican Republic | 2 | 0 | 2 | 0.3% |
| Ecuador | 6 | 0 | 6 | 0.8% |
| Guatemala | 2 | 3 | 5 | 0.6% |
| Haiti | 9 | 0 | 9 | 1.1% |
| Honduras | 2 | 1 | 3 | 0.4% |
| Jamaica | 1 | 0 | 1 | 0.1% |
| Mexico | 4 | 0 | 4 | 0.5% |
| Nicaragua | 5 | 2 | 7 | 0.9% |
| Peru | 8 | 2 | 10 | 1.3% |
| Trinidad | 1 | 0 | 1 | 0.1% |
| **The Eastern Mediterranean Region** | **39** | **22** | **61** | **7.8%** |
| Afghanistan | 8 | 4 | 12 | 1.5% |
| Egypt | 6 | 1 | 7 | 0.9% |
| Iran | 2 | 1 | 3 | 0.4% |
| Pakistan | 21 | 14 | 35 | 4.5% |
| Somalia | 1 | 0 | 1 | 0.1% |
| Syria | 1 | 1 | 2 | 0.3% |
| Yemen | 0 | 1 | 1 | 0.1% |
| **Europe** | **3** | **1** | **4** | **0.5%** |
| Armenia | 1 | 0 | 1 | 0.1% |
| Kyrgyz Republic | 1 | 0 | 1 | 0.1% |
| Turkey | 1 | 1 | 2 | 0.3% |
| **South-East Asia** | **180** | **44** | **224** | **28.5%** |
| Bangladesh | 61 | 16 | 77 | 9.8% |
| Bhutan | 1 | 0 | 1 | 0.1% |

| Region/country | Child | Maternal | Total | Percent |
|---|---|---|---|---|
| India | 70 | 15 | 85 | 10.8% |
| Indonesia | 8 | 1 | 9 | 1.1% |
| Myanmar | 2 | 1 | 3 | 0.4% |
| Nepal | 37 | 10 | 47 | 6.0% |
| Sri Lanka | 0 | 1 | 1 | 0.1% |
| Thailand | 1 | 0 | 1 | 0.1% |
| **The Western Pacific Region** | **30** | **7** | **37** | **4.7%** |
| Cambodia | 5 | 2 | 7 | 0.9% |
| China | 4 | 2 | 6 | 0.8% |
| Laos | 4 | 0 | 4 | 0.5% |
| Malaysia | 1 | 0 | 1 | 0.1% |
| Mongolia | 1 | 0 | 1 | 0.1% |
| Papua New Guinea | 2 | 1 | 3 | 0.4% |
| Philippines | 2 | 2 | 4 | 0.5% |
| Vietnam | 11 | 0 | 11 | 1.4% |

*Total number of countries is larger than 698 because some assessments were carried out in more than one country.

Table S9. Maternal health indicators from assessments included in the review of the effectiveness of community-based primary health care*

| Category of outcome | Specific indicator |
|---|---|
| **Change in mortality** | Maternal mortality ratio |
| | Number of obstetric causes of death per 10,000 women |
| | Cause-specific maternal mortality ratio |
| | Number of deaths from obstructed labor |
| | Case fatality of eclampsia cases |
| | Number of abortion-related deaths per 10,000 women |
| **Change in serious morbidity** | Incidence of post-partum hemorrhage/post-partum blood loss > 500mL or 1,000 mL |
| | Mean blood loss postpartum |
| | Incidence of post-partum "heavy vaginal bleeding" as reported by the mother or the health worker |
| | Need for transfer to a higher-level facility for blood transfusion or surgical intervention |
| | Presence of vaginal bleeding during pregnancy |
| | Incidence of obstructed labor or sequelae (i.e., obstetric fistula) |
| | Incidence of pre-eclampsia/eclampsia |
| | Incidence of edema |
| | Prevalence of anemia (variously defined) |
| | Incidence of clinical malaria/malaria parasitemia |
| | Incidence of depression |
| | Post-partum infection/puerperal sepsis/postpartum fever/maternal infection |
| | Incidence of adverse effects from misoprostol administered to prevent post-partum hemorrhage |
| | Maternal illness or obstetric complication not otherwise specified |
| | Trauma as a result of gender-based violence |
| **Change in nutritional status** | Height-for-weight index |
| | Body-mass index |
| | Mid-upper arm circumference |
| **Change in population (or target group) coverage of key child survival interventions** | |
| **Key household behaviors** | Safe delivery at home |
| | Delivery at home by a skilled attendant |
| | Participation in women's organizations |
| | Birth preparedness |
| | Pregnancy occurring during the first 24 months postpartum |
| | Use of safe delivery practices |
| | Response to obstetric emergencies |
| | Receipt of antenatal care early in pregnancy (<20 weeks) |

| Category of outcome | Specific indicator |
|---|---|
| **Utilization of key facility-based services** | Utilization of antenatal care |
| | Delivery at a facility/birthing center by a skilled provider |
| | Access to emergency Obstetric facilities |
| | Access to curative care |
| **Improvement in health care utilization or in quality of health care provided** | Contact with community health worker (CHW) for prenatal or postnatal care |
| | Receipt of intermittent preventative therapy (IPT) during pregnancy |
| | Tetanus toxoid vaccination |
| | HIV testing during pregnancy |
| | Syphilis screening during pregnancy |
| | Antiretroviral therapy for pregnant women |
| | Utilization of post-partum family planning |
| | Receipt of iron supplements during pregnancy |
| | Receipt of folate supplements during pregnancy |
| | Receipt of vitamin A post-partum |
| | Receipt of uterotonic (misoprostol) post-partum |
| | Recognition of at-risk pregnancies |
| **Change in health-related knowledge among parents/caretakers** | Knowledge about family planning methods |
| | Village-level planning for obstetric emergencies |
| | Functioning village emergency transport committees |
| | Knowledge of pregnancy danger signs |
| | Knowledge of safe birthing procedures |
| **Health system capacity** | Registration of pregnancies |
| | Knowledge of health workers in MNCH |
| | Referrals of pregnant women by CHWs to health facilities |

*Not all of the outcome measures are necessarily appropriate as outcome measures for the review but, if not, they were included in studies along with other outcome measures that were appropriate.

**Table S10. Neonatal and child health indicators from assessments included in the review of the effectiveness of community-based primary health care***

| Category of outcome | Specific indicator |
|---|---|
| **Change in mortality** | Under-5 mortality rate |
| | 1-4-year mortality rate |
| | Infant mortality rate |
| | 6-35-month mortality rate |
| | Early infant (0-3-month or 0-5-month) mortality rate |
| | Neonatal mortality rate |
| | Late (7-28-day) neonatal mortality rate |
| | Post-neonatal (1-11-month) mortality rate |
| | Perinatal mortality rate |
| | Stillbirth rate |
| | Maternal mortality ratio |
| | Asphyxia mortality rate |
| | Rate of miscarriage/spontaneous abortion |
| | DALYs (disability-adjusted life years) averted |
| | Years of expected life saved |
| | Pneumonia case fatality rate |
| | Diarrhea case fatality rate |
| | Malaria case fatality rate |
| | Neonatal tetanus case fatality rate |
| | 18-month survival probability for HIV-exposed infants |
| **Change in serious morbidity** | Pneumonia incidence rate (including prevalence of ARI, prevalence of respiratory illness, percentage of weeks with ARI symptoms) |
| | Diarrhea incidence or prevalence rate (including number of days with diarrhea, percentage of days with diarrhea, proportion of children with diarrhea during a 90-day period, average duration of diarrheal episodes, and likelihood of recovery from diarrhea at 7 and 14 days) |
| | Incidence of severe diarrhea |
| | Incidence of dysentery/ number of days with dysentery |
| | Secondary infection rate with salmonella |
| | Malaria incidence/prevalence rate (including fever incidence rates in a malaria-endemic area, prevalence of malaria parasitemia, clinical relapse following an episode of malaria, progression to severe malaria, treatment failure, or acquired splenomegaly) |
| | Prevalence of pre-term birth |
| | Prevalence of small-for-gestational birth |
| | Presence of impetigo in low-birthweight infants |
| | Prevalence of anemia |
| | Incidence of maternal/congenital syphilis |
| | Prevalence of trachoma |
| | Number of measles cases |
| | Incidence of conjunctivitis |

| Category of outcome | Specific indicator |
|---|---|
| | Incidence of acute flaccid paralysis |
| | Incidence of polio |
| | Incidence of blindness |
| | Prevalence of night blindness or xerophthalmia |
| | Incidence/prevalence of HIV infection |
| | CD4 count |
| | Mother-to-child transmission of HIV infection |
| **Change in nutritional status** | Weight for age |
| | Height for age |
| | Height for weight |
| | Body-mass index for age |
| | Mid-upper-arm circumference |
| | Sub-scapular skin fold thickness |
| | Triceps skin fold thickness |
| | Weight gain/growth velocity |
| | Weight gain after entering nutritional intervention |
| | Child weight at 12 months of age |
| | Birthweight/prevalence of low birthweight |
| | Percentage of children with severe malnutrition |
| | Nutritional status of siblings |
| | Recovery from severe acute malnutrition |
| | Prevalence of Bitot's spots |
| | Prevalence of anemia |
| | Clinical signs of iodine deficiency |
| **Progress in psychomotor development** | Achievement of developmental milestones |
| | Psychomotor development score |
| | Mental development score |
| | Presence of behavioral problems |
| **Change in population(or target group) coverage of key child survival interventions** | |
| **Key household behaviors** | Healthy water, sanitation and hygiene practices (including handwashing, presence of soap for handwashing, presence of improved water in the village or house, quality of stored water in the household, safe water storage, use of latrines, use of refuse pits, appropriate disposal of children's feces, use of face cloth to wash children's faces, and food hygiene and cleanliness scores) |

| **Category of outcome** | **Specific indicator** |
|---|---|
| | Exclusive breastfeeding in first 1-2 hours of life and in the first 6 months of life, including median length of EBF |
| | Immediate breastfeeding (within the first hour after birth) |
| | Provision of colostrum to the newborn |
| | Prevalence or duration of breastfeeding (not necessarily exclusive) |
| | Increased breastfeeding during and after a child illness |
| | Use of bottle feeding |
| | Percentage of mothers who introduce complementary feeding at 6 months of age |
| | Use of (or presence of) insecticide-treated bed nets and/or curtains, or presence of spraying in the household |
| | Adherence to newborn care practices |
| | Drying and warming the newborn immediately after birth |
| | Proper care of the umbilical cord |
| | Percentage of mothers who took no action or who gave a home remedy for newborn danger signs |
| | Presence of household gardens or improved household food production |
| | Percentage of children consuming iron-rich or vitamin-A rich foods |
| | Duration of food insecure period for a household |
| | Percentage of pregnant women eating more than usual during pregnancy |
| | Percentage of pregnant women doing heavy work |
| **Key services** | Percentage of cases of children with symptoms of severe or moderate ARI contacting a primary health care worker/care seeking for cough and difficult breathing |
| | Percentage of cases of pneumonia receiving antibiotics |
| | Percentage of cases of pneumonia receiving early and appropriate treatment |
| | Percentage of cases of diarrhea receiving ORS or recommended home fluids |
| | Percentage of cases of diarrhea receiving continued feeding |
| | Percentage of cases of diarrhea receiving zinc supplementation |
| | Percentage of cases of malaria receiving early, appropriate treatment/percentage of children with fever receiving treatment within 24 hours |
| | Percentage of malaria cases treated by a CHW who need referral on the 3rd day of treatment |
| | Percentage of pregnant women achieving compliance with intermittent preventive treatment of malaria |
| | Percentage of infants achieving compliance with intermitted preventive treatment of malaria |

| Category of outcome | Specific indicator |
|---|---|
| | Percentage of children/mothers with immunizations (the complete series or specific antigens such as measles, polio, tetanus toxoid, or DPT; immunization drop-out rate) |
| | Percentage of 6-59-month-old children receiving supplemental vitamin A during the previous 6 months |
| | Receipt of nutritional supplement |
| | Percentage of children with severe acute malnutrition receiving treatment |
| | Percentage of children weighed monthly |
| | Antenatal care/4 antenatal care visits |
| | Percentage of pregnant women receiving iron and folate |
| | Delivery by trained/skilled birth attendant |
| | Use of safe/clean delivery kit |
| | Health facility delivery by women with a pregnancy/birth complication |
| | Percentage of women who took misoprostol following delivery |
| | Percentage of women obtaining a post-partum visit within 7 days of birth |
| | Percentage of newborns receiving a check-up within 24 hours following delivery |
| | Utilization of family planning/contraceptive prevalence rate/adoption of family planning within 3 months of birth |
| | Uptake of voluntary counseling and testing (for HIV infection) |
| | Equity in change of population coverage (the degree to which changes in coverage are similar in different sub-populations defined by household wealth) |
| **Improvement in health care utilization or in quality of health care provided** | Utilization of health services |
| | Care seeking from unqualified providers |
| | Referral rate |
| | Percentage of ill children who are taken to a health facility or health worker for care |
| | Correct referral of pneumonia cases |
| | Percentage of ill children who obtained care at a health facility |
| | Number of child care visits to a health facility |
| | Percentage of children with diarrhea treated at a health facility who were kept in a rehydration unit and given ORS |
| | Prescription rates for ORS |
| | Percentage of providers who check children with diarrhea for at least 2 signs of dehydration |
| | Percentage of children with a Road-to-Health card |

| Category of outcome | Specific indicator |
|---|---|
| | Hospital inpatient admission rate |
| | Hospital inpatient admission rate for life-threatening malaria |
| | Percentage of cases of childhood pneumonia for which mother sought a trained health care provider |
| | Percentage of children with pneumonia who received treatment |
| | Percentage of cases of malaria treated in health facilities |
| | Percentage of pregnant women with fever for more than 3 days who sought treatment |
| | Percentage of cases of fever treated promptly |
| | Percentage of cases of malaria treated by CHWs |
| | Sales of antimalarial drugs/sales of antipyretic drugs |
| | Sales of insecticide-treated bed nets |
| | Percentage of pregnant women receiving prenatal home visits |
| | Number of antenatal care visits |
| | Laboratory exam performed at time of antenatal care visit |
| | Percentage of births taking place at a health care facility |
| | Percentage of mothers obtaining care during the post-partum period |
| | Referral of sick newborns for care |
| | Adherence to drug therapy for HIV infection |
| | Participation in women's groups |
| | Encounter with Community Health Workers/percentage of households visited by a CHW |
| | Percentage of women receiving advice from a CHW |
| | Compliance with recommendation for referral given by a CHW |
| **Change in health-related knowledge among parents/ caretakers** | Skills and knowledge about important child health issues |
| | Knowledge of serious childhood illness |
| | Knowledge about breastfeeding and its benefits |
| | Knowledge of signs of childhood pneumonia and need for treatment by a trained health care provider |
| | Knowledge about diarrhea prevention |
| | Knowledge of how to prepare oral rehydration solution |
| | Knowledge of danger signs of malaria |
| | Knowledge of the role of insecticide-treated bed nets in preventing malaria |
| | Knowledge of danger signs during pregnancy |
| | Knowledge of danger signs among newborns |
| | Knowledge about immunizations and vaccine-preventable diseases |
| | Knowledge of HIV prevention |

| Category of outcome | Specific indicator |
|---|---|
| | Knowledge about prevention of maternal-to-child transmission of HIV infection |
| | Knowledge of safe water storage |
| | Knowledge about prevention of night blindness with vitamin A supplementation |
| **Appropriateness of community case management by Community Health Workers** | Ability to detect cases of childhood pneumonia (sensitivity) |
| | Correctness of diagnosis of pneumonia (specificity) |
| | Use of timers (to measure respiratory rate) |
| | Correctness of advice for medication (including dosage) for treatment of pneumonia, diarrhea (with zinc) and malaria |
| | Error-free case management |
| | Accuracy of classification of sick children |
| | Use of rapid diagnostic tests for malaria diagnosis |
| **Health system capacity** | Presence of trained providers at the health facility |
| | Percentage of health facilities with essential drugs and supplies available |
| | Percentage of facilities implementing community-based IMCI |
| | Percentage of mothers who have access to an IMCI-trained provider |
| | Workload at the health facility |
| | District capacity to support community health services |
| | Adequacy of supervision |
| | Provision of supervision and supplies to CHWs |
| | Satisfaction of CHWs with training |
| | Formation of community committees to help with emergency transport |
| | Health centers with emergency transport in place |

*Not all of the outcome measures are necessarily appropriate as outcome measures for the review but, if not, they were included in studies along with other outcome measures that were appropriate.

Note:

ARI: acute respiratory infection

DPT: diphtheria, pertussis and tetanus

EBF: exclusive breastfeeding

HIV: human immunodeficiency virus

IMCI: Integrated Management of Childhood Illness
